# Synthesis and optical properties of new 5'-aryl-substituted 2,5-bis(3-decyl-2,2'-bithiophen-5-yl)-1,3,4-oxadiazoles

**DOI:** 10.3762/bjoc.13.34

**Published:** 2017-02-17

**Authors:** Anastasia Sergeevna Kostyuchenko, Tatyana Yu Zheleznova, Anton Jaroslavovich Stasyuk, Aleksandra Kurowska, Wojciech Domagala, Adam Pron, Alexander S Fisyuk

**Affiliations:** 1 Laboratory of New Organic Materials, Omsk State Technical University, Mira Ave, 11, 644050 Omsk, Russian Federation; 2 Department of Organic Chemistry, Faculty of Science, RUDN University, 6 Miklukho-Maklaya st., 117198 Moscow, Russian Federation; 3 Department of Chemistry and RECETOX, Masaryk University, Kamenice 5, 625 00 Brno, Czech Republic; 4 Department of Physical Chemistry and Technology of Polymers, Silesian University of Technology, Marcina Strzody 9, 44-100 Gliwice, Poland; 5 Faculty of Chemistry , Warsaw University of Technology, Noakowskiego 3, 00-664 Warszawa, Poland; 6 Department of Organic Chemistry, Omsk F.M. Dostoevsky State University, 55a Mira pr., 644077 Omsk, Russian Federation

**Keywords:** bithiophene, donor–acceptor, luminescence, 1,3,4-oxadiazole, palladium-catalyzed coupling

## Abstract

New photoluminescent donor–acceptor–donor (DAD) molecules, namely 5'-aryl-substituted 2,5-bis(3-decyl-2,2'-bithiophen-5-yl)-1,3,4-oxadiazoles were prepared by palladium-catalyzed coupling from readily available compounds such as ethyl 3-decyl-2,2'-bithiophene-5-carboxylate and aryl halides. The obtained compounds feature increasing bathochromic shifts in their emission spectra with increasing aryl-substituent size yielding blue to bluish-green emissions. At the same time, their absorption spectra are almost independent from the identity of the terminal substituent with λ_max_ values ranging from 395 to 405 nm. The observed trends are perfectly predicted by quantum chemical DFT/TDDFT calculations carried out for these new molecules.

## Introduction

**Figure 1:**
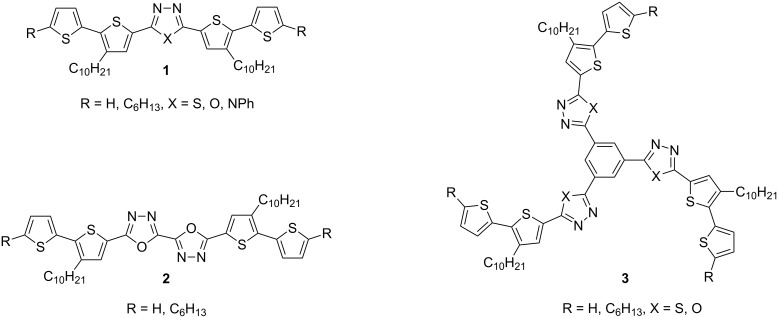
D–A compounds **1**–**3**.

π-Conjugated donor–acceptor (D-A) compounds are of significant scientific interest because they frequently combine solution processability with unique electronic, luminescent and electrochemical properties [1–5]. Molecules in which a central electron-accepting ring separates two bithiophene units are of particular interest [6–15]. The continuing progress in this field calls for elaboration of new synthetic procedures affording tailor-made molecules with tunable physical properties. In this respect, we have recently developed an efficient and flexible approach to the synthesis of π-conjugated donor–acceptor compounds [16–17]. The proposed synthetic pathway enables the preparation of linear D–A–D compounds with one or two 1,3,4-oxadiazole [16–19], 1,3,4-thiadiazole [16,18–22] or 1,2,4-triazole [16,18] central rings symmetrically disubstituted with alkylbithiophene **1**, **2** as well as star-shaped molecules with D–A arms **3** (Figure 1) [23].

We have also shown that these molecules, if the α-position in the terminal polymer ring is not blocked, can electrochemically polymerize yielding either linear polymers [17,19–20,24] or polymeric networks [23]. It has been demonstrated that the optical and electrochemical properties of these new, solution-processable and conjugated compounds can be modified in a controllable way by the nature of the electron-accepting heterocyclic ring, the position of the solubilizing alkyl substituent and the molecule topology (linear vs three-arms star) [16,19,21,23]. In particular, the synthesized molecules turned out efficient electroluminophores in organic light emitting diodes of “guest–host”-type [18,22–23].

**Scheme 1:**
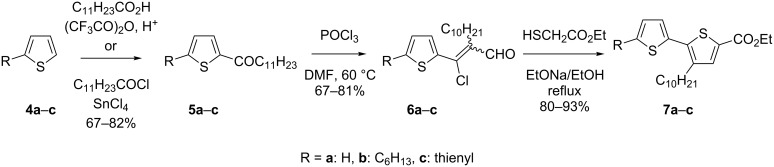
Synthesis of 3-decyl-2,2'-bithiophene-5-carboxylic acid ethyl esters **7a–c**.

In one of our previous papers [25] we proposed an original method for the preparation of ester derivatives of alkylbithiophenes **7**, some of which were then used as precursors of **1–3** (see Scheme 1)

The ethyl esters of 2,2'-bithiophenes **7** having no (**7a**), an alkyl (**7b**), or a thienyl (**7c**) substituent in the C-5' position can be easily obtained by this method. On the other hand, the substitution of the thiophene ring with phenyl or fused aromatic rings is much more challenging and requires the elaboration of a new synthetic procedure. In this communication we present a detailed study of cross-coupling reactions between еthyl 3-decyl-2,2'-bithiophene-5-carboxylate and different aryl halides. The resulting esters are subsequently used as substrates in the preparation of new D–A–D compounds with an oxadiazole central ring symmetrically functionalized with bithiophene end-capped with various aryl groups. Our preliminary studies show that these compounds can be used as electroluminophores in LEDs, which show superior performance as compared to the devices fabricated from **1** or **2** [18].

## Results and Discussion

### Synthetic toolbox

**Scheme 2:**
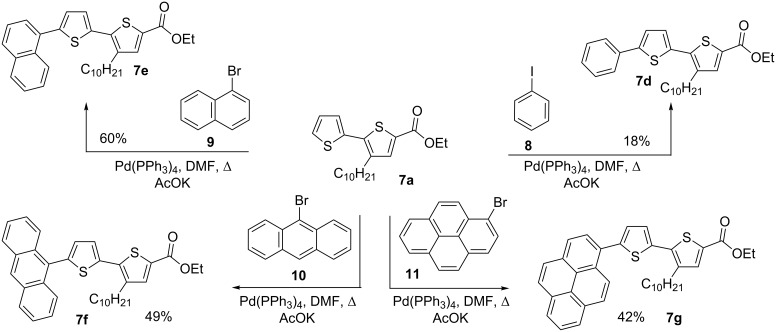
Synthesis of ethyl esters of 5'-aryl-3-decyl-2,2'-bithiophene-5-carboxylic acids **7d**–**g**.

It is well known that thiophenes containing acceptor groups such as CHO, CN or CO_2_Me at the C-2 position readily react with aryl halides in the presence of a Pd(II) catalyst through a Heck-type coupling [26]. We have synthesized the ethyl ester of 2,2'-bithiophene-5-carboxylic acid **7a** [25] to study its reactivity towards various aryl halides, namely: iodobenzene (**8**), 1-bromonaphthalene (**9**) and the 9-bromanthracene (**10**) and 1-bromopyrene (**11**) (see Scheme 2).

Initial attempts to carry out the reaction under the same conditions as described in [26], i.e., heating a mixture of **7a** with **8** or **9** in dimethylacetamide in the presence of Pd(OAc)_2_, Bu_4_N^+^Br^−^ and K_2_CO_3_, led to inseparable mixtures. Further, compounds **7d**–**g** could not be isolated when the base K_2_CO_3_ was replaced with Cs_2_CO_3_ and PPh_3_ was used as a ligand [27]. Gratifyingly, the 5'-arylated products **7d–g** were obtained by heating a mixture of ethyl 3-decyl-2,2'-bithiophene-5-carboxylate (**7a**) with aryl halides in the presence of tetrakis(triphenylphosphine)palladium(0) and AcOK in DMF for 20–22 h. Unfortunately, the yield of the phenyl-substituted product **7d** remained low (18%), even when the reaction was performed for 55 h after which time all **7a** had been consumed as judged by TLC. The other products **7e–g** could be isolated in 42–60% yield after purification by column chromatography (Table 1).

**Table 1:** Reaction conditions and yields for the synthesis of ethyl 5'-aryl-3-decyl-2,2'-bithiophene-5-carboxylates **7d**–**g**.

Entry	Compound	Reaction conditions	Yield, %

1	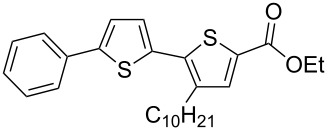 **7d**	DMF, AcOK, 130 °C, 55 h, Pd(PPh_3_)_4_	18
2	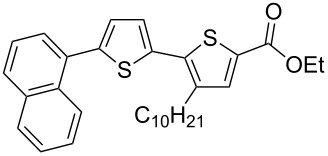 **7e**	DMF, AcOK, 130 °C, 28 h, Pd(PPh_3_)_4_	60
3	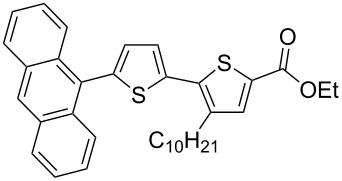 **7f**	DMF, AcOK, 130 °C, 20 h, Pd(PPh_3_)_4_	49
4	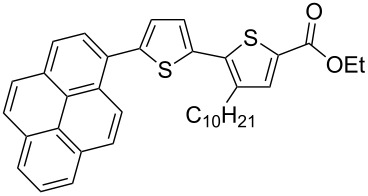 **7g**	DMF, AcOK, 130 °C, 23 h, Pd(PPh_3_)_4_	42

**Scheme 3:**
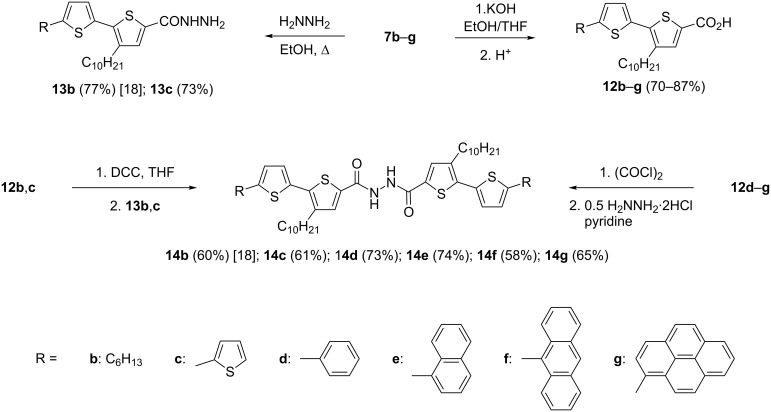
Synthesis of diacyl hydrazines **14b**–**g**.

With the building blocks **7b–g** at hand, we next turned towards the synthesis of 1,3,4-oxadiazole derivatives **15b–g** with anticipated semiconducting and photoluminescent properties. First, alkaline hydrolysis of the esters **7b–g** led to the corresponding carboxylic acids **12b–g** in good yields (70–87%, Scheme 3). The corresponding hydrazide derivatives **13b**,**c** were obtained in 67–73% yields by refluxing the esters **7b**,**c** with hydrazine monohydrate in alcohol. The diacyl hydrazines **14b**,**c** were obtained through the coupling of the hydrazide derivatives **13b**,**c** with carboxylic acids **12b**,**c** in the presence of *N*,*N*'-dicyclohexylcarbodiimide (DCC) as reported earlier [18]. A modified procedure, which avoids the preparation of hydrazides **13**, was used for the synthesis of **14d**–**g**. These intermediates were synthesized by the reaction of the acid chlorides with hydrazine dihydrochloride in the presence of pyridine. The required acid chlorides were prepared in situ through the reaction of the corresponding carboxylic acids **12b**–**g** with oxalyl chloride. The yields of the obtained diacylated hydrazines **14b**–**g** were in the range of 58–74% (Scheme 3).

**Scheme 4:**
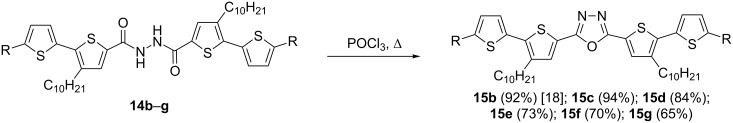
Synthesis of 5'-aryl-substituted 2,5-bis(3-decyl-2,2'-bithiophen-5-yl)-1,3,4-oxadiazoles **15b**–**g**.

Finally, heating the diacyl hydrazines **14b**–**g** in phosphorus oxychloride led to the formation of the desired 1,3,4-oxadiazoles **15b**–**g**. The product yields were between 65–94% after purification by column chromatography (Scheme 4).

All intermediates and final products were identified by elemental analysis, NMR and IR spectra, which are presented in Supporting Information File 1.

### Spectroscopic and luminescent properties of the synthesized compounds

**Figure 2:**
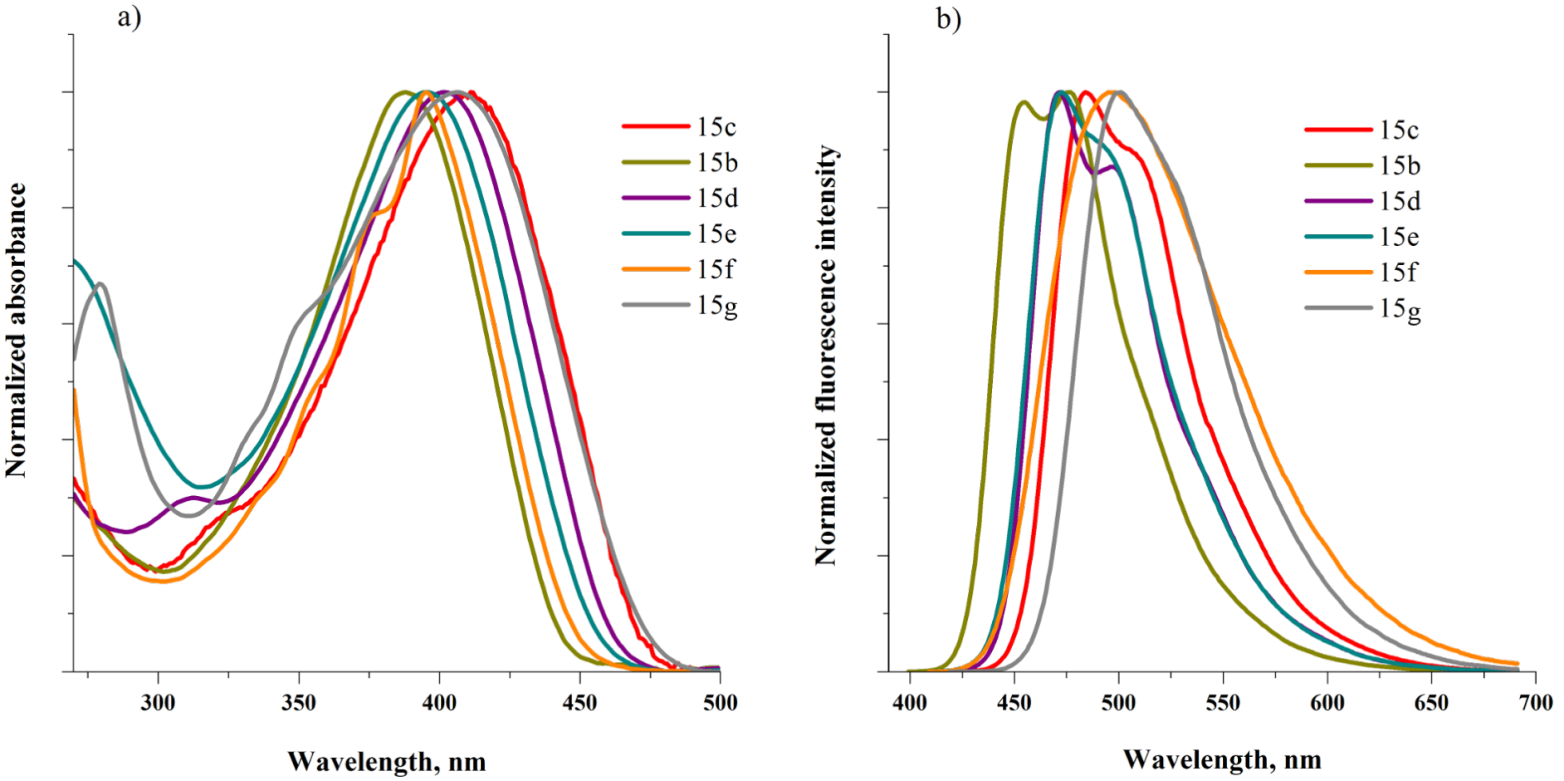
5'-Aryl-substituted 2,5-bis(3-decyl-2,2'-bithiophen-5-yl)-1,3,4-oxadiazoles **15b**–**g**: a) absorption spectra, b) photoluminescence spectra in DCM.

The 1,3,4-oxadiazole derivatives **15b**–**g** are powders of yellow to orange color and their solutions exhibit fluorescent properties. The absorption and fluorescence spectra registered for dichloromethane (DCM) solutions are presented in Figure 2 and the obtained spectral parameters are collected in Table 2.

**Table 2:** Spectroscopic and luminescent properties of 5'-aryl-substituted 2,5-bis(3-decyl-2,2'-bithiophen-5-yl)-1,3,4-oxadiazoles **15b**–**g** in DCM.

Compound	UV–vis		photoluminescence				

	λ_max,abs_^a^	*E*_g_^opt b^	λ_ex_^c^	λ_max,em_^d^	Stokes shift^e^ Δ		Ф_F_^f^
	[nm]	[eV]	[nm]	[nm]	[nm]	[eV]	

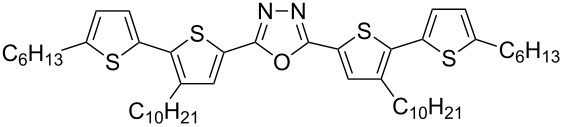 **15b**	389	2.81	377	448; 472	59	0.52	0.84
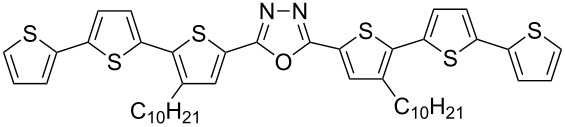 **15c**	409	2.62	396	484; 507	75	0.57	0.20
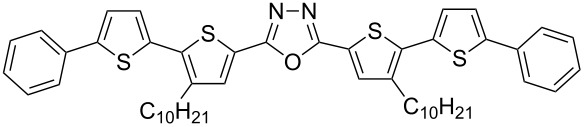 **15d**	403	2.69	390	468; 496	65	0.53	0.49
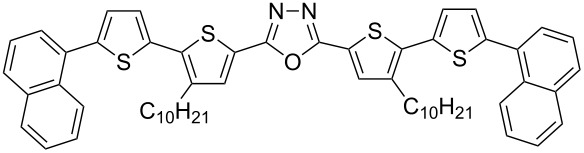 **15e**	397	2.74	381	473; 496	76	0.63	0.25
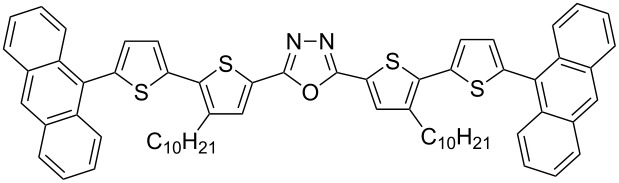 **15f**	395	2.78	372	499	104	0.85	0.18
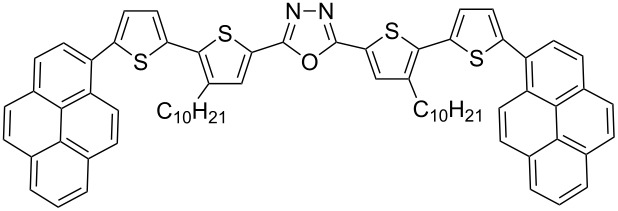 **15g**	406	2.63	380	501	95	0.79	0.29

^a^Absorption, ^b^pertaining to onset of the π–π* absorption peak, ^c^excitation, ^d^emission, ^e^minimum value, ^f^quantum yield determined relative to 9,10-diphenylanthracene as the standard.

All compounds display an intense absorption band with a maximum between 389 and 409 nm, ascribed to the π–π* transition. A comparison of the absorption spectra of **15b** and **15c–g** indicates that the presence of a thiophene ring or carboaromatic substituents at the C-5' position in the 2,5-bis(2,2'-bithiophen-5-yl)-1,3,4-oxadiazole leads to a slight bathochromic shift of the absorption band as compared to the corresponding alkyl derivative (389 nm). The largest shift of λ_max_ is observed in the spectra of the 5’-thienyl- (409 nm), 5’-phenyl- (403 nm) and 5'-pyrenyl (406 nm) derivatives **15c**,**d**,**g** (Table 2).

As evidenced by the data presented in Table 2, the optical band gaps (Δ*E*_g_^opt^) for the synthesized oxadiazole derivatives are almost independent of the carboaromatic substituents’ size. However, in all cases they are lower than the band gap of the unsubstituted 2,5-bis(3-decyl-2,2'-bithiophen-5-yl)-1,3,4-oxadiazole (2.87 eV) [16] and of the 5'-alkyl-substituted derivative **15b** (2.81 eV). This rather weak dependence of the band gap on the aromatic substituent size observed for compounds **15d–g**, can be explained by two opposite effects which partly compensate each other: larger substituents increase the number of π-bonds being in conjugation but at the same time they induce non-planarity due to steric hindrance, which lowers the effective conjugation. This problem will be discussed in detail in the section devoted to quantum chemical calculations.

When excited by UV light, all studied compounds emit blue (468, 473 nm) or bluish-green (499, 501) light (Table 2). Moderate to large values of Stokes shifts are observed for compounds **15b**–**g** originating from bond order switching (benzenoid to quinoid) in the excited state. Moderate values of Stokes shifts (0.52–0.63 eV) are observed for **15b–e** and the Stokes shift increases with an increase of the aryl substituent size. As expected, the largest Stokes shift values of 0.85 eV and 0.79 eV are observed for **15f** and **15g**, i.e., the derivatives with the largest substituents anthracen-9-yl (0.85 eV) and pyren-1-yl (0.79 nm), respectively. Note the clear vibrational structure in the spectra of **15b–e** which is nonexistent in the case of **15f**,**g** (Figure 2). The measured photoluminescence quantum yields decrease in the following order: **15b** (0.84) > **15d** (0.49) > **15g** (0.29) > **15e** (0.25) > **15c** (0.20) ≈ **15f** (0.18), indicating that for larger substituents the non-radiative rate constant increases. Such phenomena are frequently observed in D–A–D-type conjugated molecules [28].

### DFT calculations

**Figure 3:**
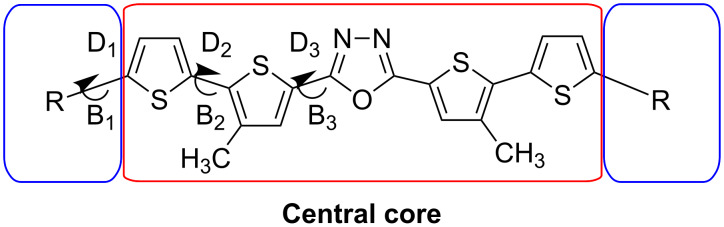
General structure of 5'-aryl-substituted 2,5-bis(3-decyl-2,2'-bithiophen-5-yl)-1,3,4-oxadiazoles.

To gain a deeper understanding of the electronic and photophysical properties of the synthesized 5'-aryl-substituted 2,5-bis(3-decyl-2,2'-bithiophen-5-yl)-1,3,4-oxadiazoles we have performed quantum-chemical calculations for four derivatives having a thienyl (**15c**), phenyl (**15d**), naphthalen-1-yl (**15e**) and anthracen-9-yl (**15f)** aromatic substituent. The DFT/TDDFT approach at the B3LYP/Def2-SVPD level of theory coupled with polarizable continuum model of solvent effects and using the integral equation formalism variant (IEFPCM) was applied. Figure 3 shows a general representation of the compounds studied in this work.

At the initial stage, a systematic conformational analysis of the series of substituted 1,3,4-oxadiazoles was conducted with the goal to determine the lowest-energy conformer(s). It has been found that **15c** and **15d** can exist only as a single conformer of planar structure. An increasing conformational variety has been observed for derivatives with substituents of increasing size. Thus, **15e** can exist in two conformations with different orientation of the terminal substituents with respect to the central core. At the same time, unsymmetrical substituents lead to additional conformational variety, e.g., the naphtalen-1-yl-substituted derivative **15e** can be represented by five different conformers. Table 3 collects selected geometrical parameters for the studied conformers, whereas their graphical representations are shown in Figure 3 (detailed geometrical structures of the studied compounds **15c**–**f** are shown in Supporting Information File 1, Figure S2).

**Table 3:** Selected geometrical parameters (bond lengths, B and dihedral angles, D) for all found conformers of the studied compounds^a^, as well as relative energies, *E*_rel_, for each conformer in the ground state obtained for DCM solution (IEFPCM) at the B3LYP/Def2-SVPD level of theory.

№	Dihedral Angle (D), degree			Bong length (B), Å			Relative energy (E_rel_)
	|D_1_|	|D_2_|	|D_3_|	B_1_	B_2_	B_3_	kcal/mol

**15c**	0.01	0.00	0.01	1.4495	1.4477	1.4362	E_rel_ = 0
**15d**	0.01	0.01	0.00	1.4691	1.4484	1.4363	E_rel_ = 0
**15e**	Conformer SS1						
	55.84	6.71	0.34	1.4770	1.4504	1.4353	E_rel_ = 1.07
	Conformer SS2						
	57.18	3.30	0.08	1.4774	1.4505	1.4366	E_rel_ = 1.05
	Conformer AS						
	56.31	0.16	0.59	1.4771	1.4503	1.4366	E_rel_ = 0.57
	Conformer AA1						
	47.24	9.80	0.31	1.4749	1.4499	1.4365	E_rel_ = 0.11
	Conformer AA2 (most stable)						
	47.54	13.33	1.35	1.4749	1.4501	1.4566	E_rel_ = 0
**15f**	Conformer A						
	88.62	22.34	0.20	1.4842	1.4531	1.4374	E_rel_ = 0.13
	Conformer S (most stable)						
	88.64	8.05	0.85	1.4842	1.4515	1.4367	E_rel_ = 0

^a^*n*-Decyl groups in the compounds were replaced with CH_3_ throughout to simplify the calculations.

For derivatives **15c** and **15d** in which the substituent consists of one aromatic ring, the whole 1,3,4-oxadiazole molecule is flat, since the dihedral angles D_1_, D_2_ and D_3_ are very close to zero. However, in case of **15e** and **15f** a substantial non-planarity is induced by the larger substituents as evidenced by an increase of their dihedral angles values. Note, that the anthracenyl substituent is almost perpendicular to the adjacent thienylene ring (D_1_ is approximately 88°) [29]. This dihedral angle is “a compromise” between the conjugation of the adjacent segments which favors the molecule’s planarity and steric hindrance effects which induce torsion. In the case of larger sized substituents, the steric effect predominates leading to large values of dihedral angles and limiting the conjugation to the substituent itself. It is worth mentioning, that the size of the aryl substituents also affects the whole molecule’s geometry since D_2_ and D_3_ angles also increase for larger substituents.

DFT and TDDFT approaches have proven to be very efficient and versatile tools for the investigation of both ground and excited-state properties of various organic and inorganic compounds [30–31]. However, it has been shown that the description of π–π* transitions for bithiophene derivatives of 1,3,4-oxadiazoles still remains challenging. Grimme and Dierksen [32] have demonstrated that calculations performed by the hybrid B3LYP functional with a triple-ξ quality basis set almost perfectly describe singlet–singlet π–π* transitions for 2,2':5',2'':5'',2''':5''',2''''-quinquethiophene and 2,5-diphenylfuran. They have further demonstrated that the basis set of double-ξ quality shows a small decrease of the displacement of the obtained spectrum compared to triple-ξ quality, while the larger basis sets yield almost identical results. The hybrid B3LYP functional coupled with an integral equation formalism of the polarizable continuum model (IEFPCM) provides a reasonable and qualitatively adequate description of vertical excitation processes for bithiophene derivatives of 1,3,4-oxadiazoles [21].

The absorption spectra of the investigated compounds measured in DCM demonstrate a featureless band with maxima allocated in the range from 395 to 409 nm. The maximum of absorption ascribed to the π–π* transition for the studied thiophene, phenyl, naphthalene and anthracene-substituted derivatives, shows a small hypsochromic shift with increasing spatial volume of the substituent R. This observation is in a full accordance with the aforementioned relationship of the molecule planarity and aromatic conjugation.

**Figure 4:**
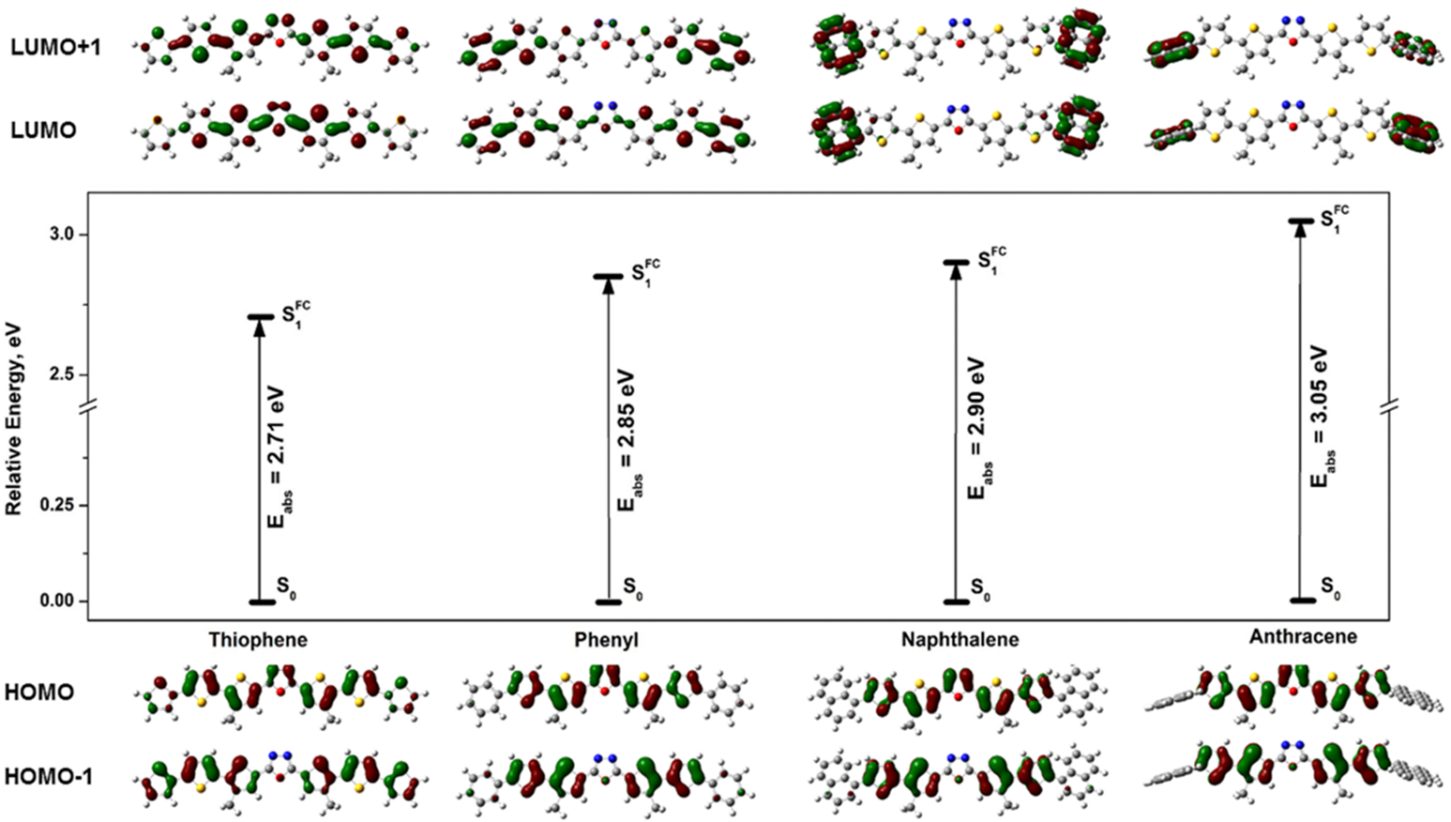
Frontier molecular orbitals (HOMO−1, HOMO, LUMO and LUMO+1) and values for HOMO–LUMO band gaps of the investigated molecules calculated at the IEFPCM(DCM)-CAM-B3LYP/Def2-SVPD level of theory. The isovalue of each depiction is equal to 0.03 e/au^3^.

Initially, for optical transition simulations, we have used the same functional and basis set as has been used for the ground state geometry optimization, i.e., B3LYP/Def2-SVPD/IEFPCM(DCM). However, the obtained excitation energies revealed an extremely poor agreement between the quantum-chemical predictions and the experiment. According to the theoretical results the observed transition is solely connected with the HOMO–LUMO transition. A careful analysis of frontier molecular orbitals disclosed a notable charge transfer from the central part to the terminal substituents (Figure 4).

Considering the fact that the non-coulombic part of hybrid functionals typically dies off too quickly resulting in lack of precision at large distances and lack of suitability for some tasks associated with electron-excitation modeling, we repeated our calculations with the range-separated hybrid CAM-B3LYP functional. Indeed, the CAM-B3LYP functional provides values of the transition energies which are well correlated with the experimentally measured optical band gap (determined from the absorption spectrum). The analysis of vertical excitation shows that several molecular orbitals are involved in this process, but the main contribution (estimated by coefficients of the wave function for each excitation) comes from the HOMO–LUMO (predominantly) and HOMO−1–LUMO+1 transitions. For thiophene-substituted derivatives, the HOMO is delocalized over the whole molecule, while the LUMO is mostly presented in the central part of molecule. In turn, for the other examined molecules, the situation is more or less reverse. The HOMO is localized mainly in the central part of molecules (central core), while the LUMO is concentrated on the substituents R with a small electron density on the 1,3,4-oxadiazole fragment (Figure 4). The peculiarity of the HOMO–LUMO localization for thiophene and phenyl substituents can be rationalized taking into account the fact that the thiophene core is electron rich and therefore can donate electron density to the core. It has to be mentioned that together with increasing size of the substituent and, as a consequence, dihedral angles between the substituent and the central part of the molecule, the difference in electron-density localization becomes more pronounced. In case of the anthracene substituent an almost complete localization of the HOMO on the central core and the LUMO on the anthracene fragment is observed.

## Conclusion

To summarize, we have shown that ethyl esters of 5'-aryl 3-decyl-2,2'-bithiophene-5-carboxylic acids can be prepared by the palladium-catalyzed coupling of readily available compounds, namely ethyl 3-decyl-2,2'-bithiophene-5-carboxylate and aryl halides. Using these building blocks the synthesis of new fluorescent conjugated 5'-aryl-substituted 2,5-bis(3-decyl-2,2'-bithiophen-5-yl)-1,3,4-oxadiazoles was developed. DFT calculations of the 5'-aryl-substituted 2,5-bis(3-decyl-2,2'-bithiophen-5-yl)-1,3,4-oxadiazoles in methylene chloride indicated that large-sized terminal substituents such as naphth-1-yl or anthracen-9-yl induce twisting of the molecules’ segments due to increasing steric hindrance. The quantum-computational results perfectly predicted the observed experimental trends.

## Supporting Information

File 1 Experimental, computational and analytical data

## References

[1] Balan A, Gunbas G, Durmus A, Toppare L (2008). Chem Mater.

[2] Mishra A, Ma C-Q, Bäuerle P (2009). Chem Rev.

[3] Wang C, Dong H, Hu W, Liu Y, Zhu D (2012). Chem Rev.

[4] Pron A, Gawrys P, Zagorska M, Djurado D, Demadrille R (2010). Chem Soc Rev.

[5] Bujak P, Kulszewicz-Bajer I, Zagorska M, Maurel V, Wielgus I, Pron A (2013). Chem Soc Rev.

[6] McCairn M C, Kreouzis T, Turner M L (2010). J Mater Chem.

[7] Mitschke U, Mena Osteritz E, Debaerdemaeker T, Sokolowski M, Bäuerle P (1998). Chem – Eur J.

[8] Mitschke U, Debaerdemaeker T, Bäuerle P (2000). Eur J Org Chem.

[9] Lee T, Landis C A, Dhar B M, Jung B J, Sun J, Sarjeant A, Lee H-J, Katz H E (2009). J Am Chem Soc.

[10] Clavier G, Audebert P (2010). Chem Rev.

[11] Gong Y-H, Miomandre F, Méallet-Renault R, Badré S, Galmiche L, Tang J, Audebert P, Clavier G (2009). Eur J Org Chem.

[12] Ellinger S, Graham K R, Shi P, Farley R T, Steckler T T, Brookins R N, Taranekar P, Mei J, Padilha L A, Ensley T R (2011). Chem Mater.

[13] Crouch D J, Skabara P J, Lohr J E, McDouall J J W, Heeney M, McCulloch I, Sparrowe D, Shkunov M, Coles S J, Horton P N (2005). Chem Mater.

[14] Crouch D J, Skabara P J, Heeney M, McCulloch I, Coles S J, Hursthouse M B (2005). Chem Commun.

[15] Sonar P, Santamaria S G, Lin T T, Sellinger A, Bolink H (2012). Aust J Chem.

[16] Kostyuchenko A S, Yurpalov V L, Kurowska A, Domagala W, Pron A, Fisyuk A S (2014). Beilstein J Org Chem.

[17] Fisyuk A S, Demadrille R, Querner C, Zagorska M, Bleuse J, Pron A (2005). New J Chem.

[18] Kostyuchenko A S, Wiosna-Salyga G, Kurowska A, Zagorska M, Luszczynska B, Grykien R, Glowacki I, Fisyuk A S, Domagala W, Pron A (2016). J Mater Sci.

[19] Kurowska A, Kostyuchenko A S, Zassowski P, Skorka L, Yurpalov V L, Fisyuk A S, Pron A, Domagala W (2014). J Phys Chem C.

[20] Kotwica K, Kurach E, Louarn G, Kostyuchenko A S, Fisyuk A S, Zagorska M, Pron A (2013). Electrochim Acta.

[21] Zapala J, Knor M, Jaroch T, Maranda-Niedbala A, Kurach E, Kotwica K, Nowakowski R, Djurado D, Pecaut J, Zagorska M (2013). Langmuir.

[22] Grykien R, Luszczynska B, Glowacki I, Kurach E, Rybakiewicz R, Kotwica K, Zagorska M, Pron A, Tassini P, Grazia Maglione M (2014). Opt Mater.

[23] Kotwica K, Kostyuchenko A S, Data P, Marszalek T, Skorka L, Jaroch T, Kacka S, Zagorska M, Nowakowski R, Monkman A P (2016). Chem – Eur J.

[24] Levi M D, Fisyuk A S, Demadrille R, Markevich E, Gofer Y, Aurbach D, Pron A (2006). Chem Commun.

[25] Kostyuchenko A S, Averkov A M, Fisyuk A S (2014). Org Lett.

[26] Gozzi C, Lavenot L, Ilg K, Penalva V, Lemaire M (1997). Tetrahedron Lett.

[27] Yokooji A, Satoh T, Miura M, Nomura M (2004). Tetrahedron.

[28] Wiosna-Salyga G, Gora M, Zagorska M, Toman P, Luszczynska B, Pfleger J, Glowacki I, Ulanski J, Mieczkowski J, Pron A (2015). RSC Adv.

[29] Fraind A M, Sini G, Risko C, Ryzhkov L R, Bredas J-L, Tovar J D (2013). J Phys Chem B.

[30] Adamo C, Jacquemin D (2013). Chem Soc Rev.

[31] Jacquemin D, Mennucci B, Adamo C (2011). Phys Chem Chem Phys.

[32] Dierksen M, Grimme S (2004). J Phys Chem A.

