# Supplementary material for: Synthesis and optical properties of new 5'-aryl-substituted 2,5-bis(3-decyl-2,2'-bithiophen-5-yl)-1,3,4-oxadiazoles
Source: Beilstein J Org Chem. 2017 Feb 17;13:313–22. doi: 10.3762/bjoc.13.34 (PMC5331275; doi:10.3762/bjoc.13.34)

Supporting Information

for

Synthesis and optical properties of new 5'-aryl-substituted 2,5-bis(3-decyl-2,2'-bithiophen-5-yl)-1,3,4-oxadiazoles

Anastasia S. Kostyuchenko[1,2], Tatyana Yu. Zheleznova[1], Anton J. Stasyuk[3], Aleksandra Kurowska[4], Wojciech Domagala[4], Adam Pron[5]* and Alexander S. Fisyuk*[1,6]

Address:[1] Laboratory of New Organic Materials, Omsk State Technical University, Mira Ave, 11, 644050 Omsk, Russian Federation, [2]Department of Organic Chemistry, Faculty of Science, RUDN University, 6 Miklukho-Maklaya st.,117198 Moscow, Russian Federation, [3]Department of Chemistry and RECETOX, Masaryk University, Kamenice 5, 625 00 Brno, Czech Republic, [4]Department of Physical Chemistry and Technology of Polymers, Silesian University of Technology, Marcina Strzody 9, 44-100 Gliwice, Poland, [5]Faculty of Chemistry , Warsaw University of Technology, Noakowskiego 3, 00-664 Warszawa, Poland and [6]Department of Organic Chemistry, Omsk F.M. Dostoevsky State University, 55a Mira pr., 644077 Omsk, Russian Federation

Email: Alexander S. Fisyuk - fisyuk@chemomsu.ru; Adam Pron - apron@ch.pw.edu.pl

*Corresponding author

Experimental details and copies of spectra

Table of Contents:

General information....................S2

Experimental procedures and analytical data....................S3

Absorption and fluorescence spectra....................S19

References....................S31

NMR spectra of compounds....................S33

General information

The IR spectra were recorded on an Infralum FT-801 spectrometer as KBr pellets for solids, or as thin films for liquid compounds. UV–vis spectra were taken in dichloromethane solutions using a Lambda750 diode-array spectrometer, while fluorescence spectra were recorded on a Cary Eclipse fluorescence spectrometer. The frontier molecular orbital (HOMO–LUMO) gaps of investigated compounds were estimated from the onset of their π–π^* absorption band. Fluorescence excitation spectra were recorded to determine the excitation wavelength at which the maximum fluorescent response is observed. Subsequent fluorescence emission spectra were recorded at the excitation wavelengths determined in this manner. The quantum yields of the examined compounds were determined relative to 9,10-diphenylanthracene - a known quantum yield standard, using comparative method [1,2]. Using this method, the quantum yield is calculated according to the following equation:

$$\Phi = \Phi_R \cdot \left(\frac{m}{m_R}\right) \cdot \left(\frac{n^2}{n_R^2}\right)$$

where: Φ is the quantum yield, *m* is the slope of origin approaching linear segment of the plot of the integrated fluorescence intensity vs. absorbance of a fluorophore solution, *n* is the refractive index of the solvent and subscript *R* refers to data for the solution of the reference fluorophore with known quantum yield – here: 9,10-

diphenylanthracene in ethanol (Φ_R = 0.950 for excitation at 330–380 nm) and in cyclohexane (Φ_R = 0.955 for excitation at 366 nm) [3]. The ^{1}H and ^{13}C NMR spectra were obtained in $CDCl_3$ with TMS as an internal standard, using a Bruker DRX 400 spectrometer (400 and 100 MHz, respectively). The ^{13}C NMR spectra were obtained in the *J*-modulation mode. The elemental analyses were carried out on a Carlo Erba 1106 CHN analyzer. The melting points were determined on a Kofler bench. The reaction course and purity of the products were checked by thin layer chromatography on Sorbfil UV-254 plates which were visualized with UV light. All chemicals were of analytical grade and purchased from Sigma–Aldrich Chemical Co.

Computational details:

Geometry optimization was carried out to explore minimum energy structures for ground state S_0 using density functional theory (DFT) and time-dependent DFT (TDDFT) with B3LYP hybrid functional [6]. Vertical excitation energy prediction was performed with range-separated hybrid CAM-B3LYP functional [4]. Ahlrichs Def2-SVPD basis set [5,6] with polarization and diffused functions was employed in both cases. In each case, to confirm the presence of the local minimum, normal mode vibrational frequencies were also calculated at the same level of theory. To simulate the effect of the solvent, geometries were optimized using a self-consistent reaction field (SCRF) approach coupled with integral equation formalism of the polarizable continuum model (IEFPCM) [8-11]. All calculations were carried out using Gaussian 09 [12].

Experimental and analytical data

The synthesis of **7a**–**c**, **12b**,**c**, **13c**, **14b**, and **15b** are described in detail in references [13,14].

Synthesis of ethyl esters of 5'-aryl 3-decyl-2,2'-bithiophene-5-carboxylic acid 7d–g

A mixture of ethyl 3-decyl-2,2'-bithiophene-5-carboxylate **7a** (2.0 g, 5.3 mmol), $Pd(PPh_3)_4$ (0.61 g, 0.53 mmol), KOAc (1.0 g, 10.6 mmol), anhydrous DMF (10 mL) and aryl halide **8–11** (5.5 mmol) was heated for 20–55 h (the completion of the reaction was monitored by TLC) at 130 °C under inert atmosphere. Then, the resulting solution was cooled to rt and poured in water (30 mL). The product was extracted into $CHCl_3$ (3 × 20 mL), dried ($MgSO_4$) and concentrated. The crude product was purified by column chromatography using a mixture of hexane/EtOAc 20:1 as the eluent.

Ethyl 3-decyl-5'-phenyl-2,2'-bithiophene-5-carboxylate (7d)

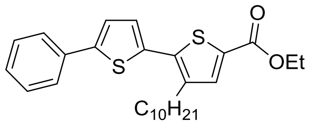

Reaction time 55 h. Yield: 0.43 g (18%); yellow-green solid; Rf = 0.56 (silica gel, hexane/EtOAc (20/1)) m.p.= 40-43^{0}C

IR (KBr). ν cm^{-1}: 1698 (C=O).

^{1}H NMR (400 MHz, $CDCl_3$, δ, ppm, J, Hz): 0.87 (t., ^{3}J=6.8, 3H, CH_3); 1.26-1.34 (m., 14H, 7CH_2,); 1.38 (t., ^{3}J=7.1, 3H, $OCH_2C\underline{H}_3$); 1.63-1.70 (m., 2H, $CH_2C\underline{H}_2C_8H_{17}$); 2.78 (t., ^{3}J=7.7, 2H, $C\underline{H}_2C_9H_{19}$); 4.34 (q., ^{3}J=7.2, 2H, $CO_2C\underline{H}_2CH_3$); 7.16 (d., ^{3}J=3.9, 1H,Th-3'-H); 7.26 (d., ^{3}J=3.9, 1H, Th-4'-H); 7.29-7.31(m., 1H, Ph-4-H); 7.36-7.40(m., 2H, Ph-3,5-H); 7.59-7.62(m., 2H, Ph-2,6-H); 7.62(s., 1H, Th-4-H).

^{13}C NMR (100 MHz, δ, ppm): 14.08 ($\underline{C}H_3$); 14.39 ($COCH_2\underline{C}H_3$); 22.69, 29.35, 29.39, 29.44, 29.48, 29.59, 29.63, 30.43, 31.93 (9CH_2); 61.13 ($CO\underline{C}H_2CH_3$); 123.58 (4′-Th); 125.85 (2,6-Ph); 127.86 (3′-Th); 127.93 (4-Ph); 129.03 (3,5-Ph); 130.78 (2-Th); 133.92 (1-Ph); 134.63 (5-Th); 135.93 (4-Th); 137.92 (2'-Th); 140.11 (3-Th); 145.49 (5'-Th); 162.20 (CO).

Elemental analysis. Calculated for $C_{27}H_{34}O_2S_2$: %C =71.32, %H =7.54; found: C =71.43%, H =7.58%.

Ethyl 3-decyl-5'-(naphthalen-1-yl)-2,2'-bithiophene-5-carboxylate (7e)

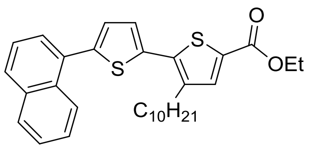

Reaction time 28 h. Yield: 1.6 g (60%); yellow-green oil; Rf = 0.62 (silica gel, hexane/EtOAc (20/1))

IR (KBr). v cm^{-1}: 1705 (C=O).

^{1}H NMR (400 MHz, $CDCl_3$, δ, ppm, J, Hz): 0.84-0.87 (m., 3H, CH_3); 1.24-1.36 (m., 14H, 7CH_2,); 1.38 (t., ^{3}J=7.1, 3H, $OCH_2C\underline{H}_3$); 1.65-1.72 (m., 2H, $CH_2C\underline{H}_2C_8H_{17}$); 2.81 (t., ^{3}J=7.8, 2H, $C\underline{H}_2C_9H_{19}$); 4.35 (q., ^{3}J=7.1, 2H, $CO_2C\underline{H}_2CH_3$); 7.21 (d., ^{3}J=3.6, 1H, Th-3'-H); 7.27 (d., ^{3}J=3.6, 1H, Th-4'-H); 7.47-7.54 (m., 3H, naphtyl-2,3,7-H); 7.58 (d.d., ^{3}J=7.0, ^{4}J=1.2, 1H, naphtyl-4-H); 7.64 (s., 1H, Th-4-H); 7.85-7.91(m., 2H, naphtyl -5,8-H); 8.25-8.29 (m., 1H, naphtyl -6-H).

^{13}C NMR (100 MHz, δ, ppm): 14.07 ($\underline{C}H_3$); 14.40 ($CO_2CH_2\underline{C}H_3$); 22.67, 29.33, 29.45, 29.50, 29.59, 29.62, 30.48, 31.91 (9CH_2), 61.14 ($CO_2\underline{C}H_2CH_3$); 125.28 (4'-Th); 125.62 (3'-Th); 126.18, 127.91, 128.82, 128.48, 126.68, 127.13, 128.18 (2,3,4,5,6,7,8-naphtyl); 130.80 (2-Th); 131.76 (5-Th); 131.80 (1-naphtyl); 134.03 (4a-naphtyl); 135.57 (2'-Th); 135.97 (4-Th); 137.90 (5'-Th); 140.08 (8a-naphtyl); 143.15 (3-Th); 162.23 (CO).

Elemental analysis. Calculated for $C_{31}H_{36}O_2S_2$: %C = 73.77, %H = 7.19; found: C = 73.82%, H = 7.23%.

Ethyl 5'-(anthracen-9-yl)-3-decyl-2,2'-bithiophene-5-carboxylate (7f)

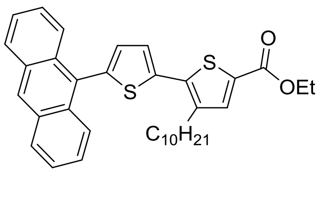

Reaction time 20 h. Yield: 1.43 g (49%); yellow-green oil; Rf = 0.31 (silica gel, hexane/EtOAc (20/1))

IR (KBr). v cm^{-1}: 1705 (C=O).

^{1}H NMR (400 MHz, $CDCl_3$, δ, ppm, J, Hz): 0.85 (t., ^{3}J=6.9, 3H, CH_3); 1.22-1.40 (m., 14H, 7CH_2); 1.40 (t., ^{3}J=7.1, 3H, $OCH_2C\underline{H}_3$); 1.66-1.74 (m., 2H, $CH_2C\underline{H}_2C_8H_{17}$); 2.84 (t., ^{3}J=7.9, 2H, $C\underline{H}_2C_9H_{19}$); 4.38 (q., ^{3}J=7.2, 2H, $CO_2C\underline{H}_2CH_3$); 7.15 (d., ^{3}J=3.5, 1H, Th-3'-H); 7.41 (d., ^{3}J=3.5, 1H, Th-4'-H); 7.44-7.51 (m., 4H, antryl-7,6,2,3-H); 7.67 (s., 1H,

Th-4-H); 7.95 (m., 2H, antryl-1,8-H); 8.04 (m., 2H, antryl-4,5-H); 8.54 (s., 1H, antryl-10-H).

^{13}C NMR (100 MHz, δ, ppm): 14.07 ($\underline{C}H_3$); 14.37 ($CO_2CH_2\underline{C}H_3$); 22.62, 29.28, 29.40, 29.45, 29.53, 29.55, 30.41, 31.84 ($9CH_2$), 61.15 ($CO_2\underline{C}H_2CH_3$); 125.30 (4'-Th); 126.12, 126.35, 127.67, 128.35, 131.18 (1,2,3,4,5,6,7,8,10-antryl); 126.86 (3'-Th); 128.30 (2-Th); 129.96 (5-Th); 130.57 (9-antryl); 131.70 (8a,9a-antryl); 136.02 (2'-Th); 136.64 (4-Th); 137.83 (5'-Th); 139.96 (4a, 10a-antryl); 140.28 (3-Th); 162.24 (CO).

Elemental analysis. Calculated for $C_{35}H_{38}O_2S_2$: %C = 75.77, %H = 6.90; found: C = 75.84%, H = 7.03%.

Ethyl 3-decyl-5'-(pyren-1-yl)-2,2'-bithiophene-5-carboxylate (7g)

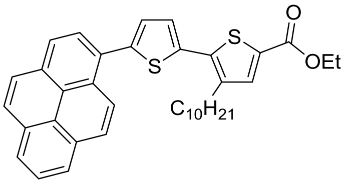

Reaction time 23 h. Yield: 1.28 g (42%); yellow-green solid; Rf = 0.23 (silica gel, hexane/EtOAc (20/1)), m.p.= 53-55^{0}C

IR (KBr). ν cm^{-1}: 1709 (C=O).

^{1}H NMR (400 MHz, $CDCl_3$, δ, ppm, J, Hz): 0.84 (t., ^3J=6.9, 3H, CH_3); 1.23-1.36 (m., 14H, $7CH_2$,); 1.40 (t., ^3J=7.1, 3H, $OCH_2C\underline{H}_3$); 1.66-1.74 (m., 2H, $CH_2C\underline{H}_2C_8H_{17}$); 2.83 (t., ^3J=7.8, 2H, $C\underline{H}_2C_9H_{19}$); 4.37 (q., ^3J=7.2, 2H, $CO_2C\underline{H}_2CH_3$); 7.34 (m., 2H, Th-3',4'-H); 7.66 (s., 1H, Th-4-H); 7.99-8.20 (m., 8H, pyrenyl-2,3,4,5,6,7,8,10-H); 8.53 (d., ^3J=9.2, 1H, pyrenyl-9-H).

^{13}C NMR (100 MHz, δ, ppm): 14.09 ($\underline{C}H_3$); 14.40 ($CO_2CH_2\underline{C}H_3$); 22.66, 29.33, 29.45, 29.50, 29.59, 29.62, 30.46, 31.89 ($9CH_2$), 61.18 ($CO_2\underline{C}H_2CH_3$); 124.63, 125.50, 126.22, 127.25, 127.30, 127.93, 128.16, 128.19, 128.41 (2,3,4,5,6,7,8,9,10-pyrenyl); 124.73 (3'-Th); 125.07, 128.92, 128.96, 130.62, 131.21, 136.05 (3a,5a,8a,10a,b,c-pyrenyl); 125.18 (4'-Th); 130.90 (2-Th); 131.43 (5-Th); 136.02 (4-Th); 137.87 (2'-Th); 140.04 (5'-Th); 143.77 (3-Th, 1-pyrenyl); 162.26 (CO).

Elemental analysis. Calculated for $C_{37}H_{38}O_2S_2$: %C = 76.78, %H = 6.62; found: C = 76.86 %, H = 6.69%.

Synthesis of 3-decyl-5'-aryl-2,2'-bithiophene-5-carboxylic acids 12d–g

To a solution of the ester **7d–g** (1.5 mmol) in THF (10 mL) was added a solution of potassium hydroxide (0.35 g, 6 mmol) in 3 mL ethanol. The reaction mixture was stirred at room temperature overnight. The solvent was removed under reduced pressure, 20 ml of water added to the residue and the mixture acidified with dilute hydrochloric acid (2 N). The formed precipitate was collected and crystallized from EtOAc.

3-Decyl-5'-phenyl-2,2'-bithiophene-5-carboxylic acid (12d)

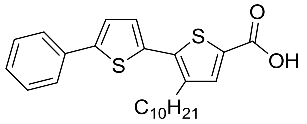

Yield: 0.48g (70%); yellow solid; m.p.=126-128 ^{0}C (EtOAc)

IR (KBr). v cm^{-1}: 1661 (C=O).

^{1}H NMR (400 MHz, $CDCl_3$): 0.87 (t., ^{3}J=6.8, 3H, CH_3); 1.26-1.44 (m., 14H, 7CH_2,); 1.64-1.72 (m., 2H, $CH_2C\underline{H}_2C_8H_{17}$); 2.80 (t., ^{3}J=7.9, 2H, $C\underline{H}_2C_9H_{19}$); 7.20 (d., ^{3}J=3.7, 1H, Th-3'-H); 7.28 (d., ^{3}J=3.7, 1H, Th-4'-H); 7.30-7.32 (m., 1H, Ph-4-H); 7.37-7.41 (m., 2H, Ph-3,5-H); 7.60-7.63 (m., 2H, Ph-2,6-H); 7.72(s., 1H, Th-4-H).

^{13}C NMR (100 MHz, δ, ppm): 14.08 ($\underline{C}H_3$); 22.69, 29.35, 29.40, 29.43, 29.47, 29.59, 29.63, 30.38, 31.93 (9CH_2); 123.65 (4′-Th); 125.89 (2,6-Ph); 128.03 (3′-Th); 128.19 (4-Ph); 129.05 (3,5-Ph); 129.29 (2-Th); 133.84 (1-Ph); 134.32 (5-Th); 137.61 (4-Th); 139.95 (2'-Th); 140.41 (3-Th), 145.92 (5'-Th); 167.28 (CO_2H).

Elemental analysis. Calculated for $C_{25}H_{30}O_2S_2$: C, 70.38%; H, 7.09%. found: C, 70.41%; H, 7.13%.

3-Decyl-5'-(naphthalen-1-yl)-2,2'-bithiophene-5-carboxylic acid (12e)

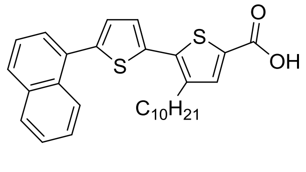

Yield: 0.59 g (83%); yellow solid; m.p.=163-165 ^{0}C (EtOAc)

IR (KBr). ν cm^{-1}: 1676 (C=O).

^{1}H NMR (400 MHz, $CDCl_3$): 0.71-0.74 (m., 3H, CH_3); 1.04-1.25 (m., 14H, 7CH_2,); 1.49 (br. s., 2H, $CH_2C\underline{H}_2C_8H_{17}$); 2.55 (br. s., 2H, 2H, $C\underline{H}_2C_9H_{19}$); 6.89 (br. s., 1H, Th-3'-H); 7.03 (br. s., 1H, Th-4'-H); 7.21-7.30 (m., 4H, naphtyl-2,3,6,7-H); 7.53(br. s., 1H, Th-4-H); 7.66-7.75 (m., 2H, naphtyl -5,8-H); 8.11 (d., ^{3}J=8.4, 1H, naphtyl -4-H).

^{13}C NMR (100 MHz, δ, ppm): 14.01 ($\underline{C}H_3$); 22.61, 29.38, 29.45, 29.84, 29.89, 30.46, 31.87 (9CH_2); 125.11 (4'-Th); 125.63 (3'-Th); 125.83, 126.39, 126.55, 127.59, 127.92, 128.18 (2,3,4,5,6,7,8-naphtyl); 131.50 (2-Th); 131.98 (5-Th); 133.92 (1-naphtyl); 134.84 (4-Th); 136.32 (2'-Th, 4a-naphtyl); 139.84 (5'-Th, 8a-naphtyl); 141.89 (3-Th); 165.97 (CO).

Elemental analysis. Calculated for $C_{29}H_{32}O_2S_2$: C, 73.07%; H, 6.77%. found: C, 73.10%; H, 6.82%.

5'-(Anthracen-9-yl)-3-decyl-2,2'-bithiophene-5-carboxylic acid (12f)

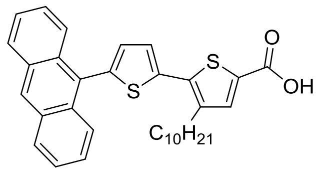

Yield: 0.68 g (86%); yellow solid; m.p.= 115-118 ^{0}C (EtOAc)

IR (KBr). ν cm^{-1}: 1670 (C=O).

^{1}H NMR (400 MHz, $CDCl_3$): 0.79 (t., ^{3}J=6.9, 3H, CH_3); 1.15-1.35 (m., 14H, 7CH_2,); 1.62-1.70 (m., 2H, $CH_2C\underline{H}_2C_8H_{17}$); 2.78 (t., ^{3}J=7.8, 2H, $C\underline{H}_2C_9H_{19}$); 7.05 (d., ^{3}J=3.5, 1H, Th-3'-H); 7.30-7.41 (m., 5H, antryl-2,3,6,7-H, Th-4'-H); 7.70 (s., 1H, Th-4-H); 7.89 (d., ^{3}J=8.8, 2H, antryl-1,8-H); 7.96 (d., ^{3}J=8.2, 2H, antryl-4,5-H); 8.46 (s., 1H, antryl-10-H).

^{13}C NMR (100 MHz, δ, ppm): 14.03 ($\underline{C}H_3$); 22.62, 29.29, 29.45, 29.58, 29.53, 29.62, 29.64, 30.45, 31.86 (9CH_2); 125.26 (4'-Th); 126.07(3'-Th); 126.41, 126.97, 128.23, 128.30, 129.97 (1,2,3,4,5,6,7,8,10-antryl); 127.76 (2-Th); 131.25 (4a,10a-antryl, 5-Th); 131.74 (8a,9a-antryl); 136.67 (4-Th); 136.84 (9-antryl); 138.51 (2'-Th); 140.20 (5'-Th); 140.28 (3-Th); 167.30 (CO_2H).

Elemental analysis. Calculated for $C_{33}H_{34}O_2S_2$: C, 75.25%; H, 6.51%. found: C, 75.34%; H, 6.57%.

3-Decyl-5'-(pyren-1-yl)-2,2'-bithiophene-5-carboxylic acid (12g)

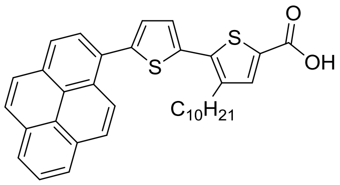

Yield: 0.72 g (87%); orange solid; m.p.= 173-175 ^{0}C (EtOAc)

IR (KBr). ν cm^{-1}: 1667 (C=O).

^{1}H NMR (400 MHz, DMSO-d6): 0.63 (t., ^{3}J=6.9, 3H, CH_3); 0.97-1.23 (m., 14H, 7CH_2,); 1.48-1.56 (m., 2H, $CH_2C\underline{H}_2C_8H_{17}$); 2.71 (t., ^{3}J=7.6, 2H, $C\underline{H}_2C_9H_{19}$); 7.40 (d., 1H, ^{3}J=3.7, Th-3'-H); 7.42 (d., 1H, ^{3}J=3.7, Th-4'-H); 7.58 (s., 1H, Th-4-H); 8.02-8.28 (m., 8H, pyrenyl-2,3,4,5,6,7,8,10-H); 8.44(d., ^{3}J=9.4, 1H, pyrenyl-9-H).

^{13}C NMR (100 MHz, δ, ppm): 13.76 ($\underline{C}H_3$); 21.96, 28.60, 28.62, 28.65, 28.88, 28.91, 29.69, 31.18 (9CH_2); 123.83, 124.24, 128.08, 130.28, 130.73, 132.56 (3a,5a,8a,10a,b,c-pyrenyl); 123.98, 125.71, 126.54, 127.19, 127.70, 127.96, 128.02, 128.34, 128.95 (2,3,4,5,6,7,8,9,10-pyrenyl); 124.90 (4'-Th); 125.27 (3'-Th); 127.94 (2-Th); 130.91 (5-Th); 135.35 (2'-Th); 135.52 (4-Th); 135.99 (5'-Th); 139.89 (3-Th); 142.50 (1-pyrenyl); 162.76 (CO_2H).

Elemental analysis. Calculated for $C_{35}H_{34}O_2S_2$: C, 76.33%; H, 6.22%. found: C, 76.40%; H, 6.28%.

Synthesis of 3-decyl-2,2':5',2''-terthiophene-5-carbohydrazide (13c)

A solution of hydrazine hydrate (97%, 0.78 mL) and **7c** (1.6 mmol) in 1.5 mL ethanol was heated under reflux for 15 h. The resulting solution was cooled to rt and water (5 mL) was added. The precipitate was collected and crystallized from methanol.

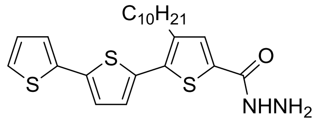

Yield: 0.52 g (73%); M.p. = 109-111 ^{0}C (methanol); yellow solid. Rf=0.32 (EtOAc/$CHCl_3$, 4/1).

IR (KBr). ν cm^{-1}: 1622 (C=O), 3160-3260 (NH).

^{1}H NMR (400 MHz, $CDCl_3$): δ =0.87 (t, ^3J=6.8, 3H, CH_3), 1.25-1.39 (m, 14H, 7CH_2), 1.60-1.67 (m, 2H, $ThCH_2C\underline{H}_2C_8H_{17}$), 2.75 (t, ^3J=7.8, 2H, $ThC\underline{H}_2C_9H_{19}$), 4.10 (s., 2H, $CONHN\underline{H}_2$), 7.02 (d.d, ^3J=3.6, ^3J=5.1, 1H, Th-4″-H), 7.06 (d., ^3J=3.9, 1H, Th-4′-H), 7.11 (d., ^3J=3.9, 1H, Th-3′-H), 7.19 (d.d, ^3J=3.6, ^4J=1.2, 1H, Th-3″-H), 7.23 (d.d, ^3J=5.1, ^4J=1.2, 1H, Th-5″-H), 7.40 (s, 1H, Th-4-H), 7.41 (s., 1H, $CON\underline{H}NH_2$).

^{13}C NMR (100 MHz, δ, ppm): 14.13 (CH_3), 22.69, 29.33, 29.36, 29.44, 29.46, 29.51, 29.58, 29.63, 30.51, 30.73, 31.91 (9CH_2), 124.05 (4′-Th), 124.07 (3′-Th), 124.90 (5’’-Th), 127.52 (3’’-Th), 127.95 (4″-Th), 131.58 (4-Th), 132.74 (2-Th), 133.72(5’-Th), 135.55 (5-Th), 136.70 (2’’-Th), 138.42 (3-Th), 140.17 (2’-Th), 163.24 (C=O).

Elemental analysis. Calculated for $C_{23}H_{30}N_2OS_3$: C, 61.85%; H, 6.77%; N, 6.27%. found: C, 61.89%; H, 6.77%; N, 6.31%.

Synthesis of *N*,*N*’-bis-(5’-aryl)-[3-decyl-2,2'-bithiophen-5-carbonyl]hydrazines 14d–g

In a similar way as described in [14], oxalyl chloride (0.62 mL, 7.2 mmol) was added to a suspension of **12d–g** (1.8 mmol) in 3 mL of dry dichloromethane under cooling (ice bath). After the addition of one drop DMF to the resulting mixture, it was stirred for 30 min under cooling, then at rt for additional 4 h. The solvent and the excess of oxalyl chloride were removed under reduced pressure and the final product (acid chloride) was used in subsequent reaction without further purification. The crude acid chloride was dissolved in 5 mL of dichloromethane and cooled (ice bath). Then, a mixture of 90 mg (0.90 mmol) of hydrazine dihydrochloride and 0.72 mL (9 mmol) of dry pyridine were added. The reaction mixture was stirred for 30 min under cooling and left overnight at rt. After removal of the solvent in vacuum, the residue was treated with 10 mL of ice water and stirred for 15 min. The precipitate was filtered, washed with water and dried. The

products were obtained after recrystallization from ethyl acetate (**14d–f**) or from a mixture of $CHCl_3$/acetone 3:1 (**14g**).

***N*,*N*'-Bis(5'-phenyl)-[3-decyl-2,2'-bithiophen-5-carbonyl]hydrazine (14d)**

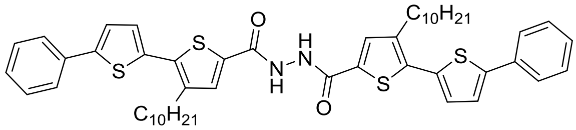

Yield: 0.56 g (73%); yellow solid; m.p.= 158-161^{0}C (EtOAc), Rf=0.28 ($CHCl_3$).

IR (KBr). ν cm^{-1}: 1627 (C=O), 3200 (NH).

^{1}H NMR (400 MHz, $CDCl_3$): 0.84 (t., ^{3}J=6.8, 6H, $2CH_3$); 1.21-1.37 (m., 28H, $14CH_2$); 1.58-1.65 (m., 4H, $2CH_2C\underline{H}_2C_8H_{17}$); 2.72 (t., ^{3}J=7.9, 4H,$2C\underline{H}_2C_9H_{19}$); 7.08 (d., ^{3}J=3.7, 2H, 2Th-3'-H); 7.18 (d., ^{3}J=3.7, 2H, 2Th-4'-H); 7.25-7.38 (m., 6H, 2Ph-3,5-H, 2Ph-4-H); 7.53-7.55 (m., 4H, 2Ph-2,6-H); 7.61 (s., 2H, 2Th-4-H); 9.52 (s., 2H, 2NH).

^{13}C NMR (100 MHz, δ, ppm): 14.08 ($\underline{C}H_3$); 22.69, 29.37, 29.44, 29.46, 29.53, 29.61, 29.67, 30.37, 31.95 (CH_2); 123.53 (4′-Th); 125.77 (2,6-Ph); 127.84 (3′-Th); 127.89 (4-Ph); 128.97 (3,5-Ph); 131.84 (2-Th); 132.68 (4-Th); 133.90 (1-Ph); 134.44 (5-Th); 137.33 (2'-Th); 140.35 (3-Th), 145.43 (5'-Th); 161.01 (CO).

Elemental analysis. Calculated for $C_{50}H_{60}N_2O_2S_4$: C, 70.71%; H, 7.12%; N, 3.30%. found: C, 70.83%; H, 7.15%; N, 3.36%.

***N*,*N*'-Bis(5'-(naphthalen-1-yl)[3-decyl-2,2'-bithiophen-5-carbonyl]hydrazine (14e)**

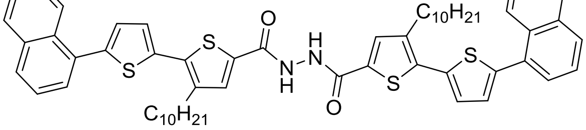

Yield: 0.63 g (74%); yellow solid; m.p.= 143-145 ^{0}C (EtOAc), Rf= 0.31(CH_2Cl_2).

IR (KBr). ν cm^{-1}: 1628 (C=O), 3206 (NH).

^{1}H NMR (400 MHz, $CDCl_3$): 0.82 (t, ^{3}J=6.8, 6H, $2CH_3$); 1.19-1.38 (m., 28H, $14CH_2$); 1.61-1.68 (m, 4H, $2CH_2C\underline{H}_2C_8H_{17}$); 2.76 (t., ^{3}J=7.8, 4H, $2C\underline{H}_2C_9H_{19}$); 7.13 (d., ^{3}J=3.7, 2H, 2Th-3'-H); 7.21 (d., ^{3}J=3.7, 2H, Th-4'-H); 7.38-7.52 (m., 8H, 2naphtyl-2,3,6,7-H);

7.66(s., 2H, 2Th-4-H); 7.80-7.87 (m., 4H, 2naphtyl-5,8-H); 7.22 (d., ^3J=8.2, 2H, 2naphtyl -4-H); 9.80 (s., 2H, 2NH).

^{13}C NMR (100 MHz, δ, ppm): 14.07 ($\underline{C}H_3$); 22.66, 29.35, 29.47, 29.59, 29.65, 30.45, 31.92 ($9CH_2$); 125.23 (4'-Th); 125.60 (3'-Th); 126.10, 126.61, 127.22, 127.85, 128.12, 128.41, 128.70 (2,3,4,5,6,7,8-naphtyl); 131.66 (2-Th); 131.77 (5-Th); 131.96 (1-naphtyl); 132.79 (4-Th); 134.00 (2'-Th); 135.40 (5'-Th); 137.31 (4a-naphtyl); 140.36 (8a-naphtyl); 143.10 (3-Th); 161.21 (CO).

Elemental analysis. Calculated for $C_{58}H_{64}N_2O_2S_4$: C, 73.38%; H, 6.79%; N, 2.95%. found: C, 73.33%; H, 6.78%; N, 2.98%.

***N*,*N'*-Bis(5'-(anthracen-1-yl)-[3-decyl-2,2'-bithiophen-5-carbonyl]hydrazine (14f)**

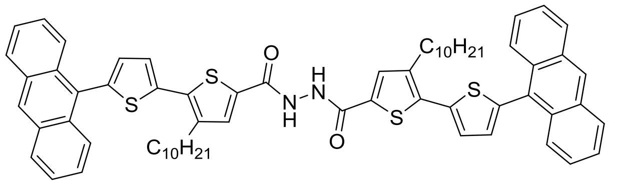

Yield: 0.55 g (58 %); yellow solid; m.p.= 170-173 ^{0}C (EtOAc), Rf= 0.58 (CH_2Cl_2). **IR (KBr). ν cm^{-1}:** 1591 (C=O), 3172 (NH).

^{1}H NMR (400 MHz, DMSO-d$_6$): 0.77 (t., ^3J=6.8, 6H, $2CH_3$); 1.16-1.37 (m., 28H, $14CH_2$); 1.64-1.72 (m., 4H, $2CH_2C\underline{H}_2C_8H_{17}$); 2.78 (t., ^3J=7.6, 4H, $2C\underline{H}_2C_9H_{19}$); 7.27 (d., ^3J=3.5, 2H, 2Th-3'-H); 7.49-7.56 (m., 10H, 2antryl-2,3,6,7-H, 2Th-4-H); 7.84 (d., ^3J=3.5, 2H, 2Th-4'-H); 7.85 (d., ^3J=8.0, 4H, 2antryl-1,8-H); 8.15 (m., ^3J=8.8, 4H, 2antryl-4,5-H); 8.73 (s., 2H, 2antryl-10-H); 10.44 (s., 2H, 2NH).

^{13}C NMR (100 MHz, δ, ppm): 13.33 ($\underline{C}H_3$); 21.52, 28.14, 28.26, 28.29, 28.44, 28.47, 28.50, 29.32, 30.77(CH_2); 125.19 (4'-Th); 126.46 (2-Th); 127.08 (3'-Th); 125.08, 126.19, 128.03, 128.12, 130.21 (1,2,3,4,5,6,7,8,10-antryl); 130.51 (4a,10a-antryl); 130.73 (8a,9a-antryl); 131.74 (4-Th); 134.63 (5-Th); 134.64 (9-antryl); 135.64 (2'-Th); 138.88 (5'-Th); 139.71 (3-Th); 160.34 (CO).

Elemental analysis. Calculated for $C_{66}H_{68}N_2O_2S_4$: C, 75.53%; H, 6.53%; N, 2.67%. found: C, 75.61%; H, 6.59%; N, 2.63%.

_N,N'_-Bis(5'-(pyren-1-yl)-[3-decyl-2,2'-bithiophen-5-carbonyl]hydrazine (14g)

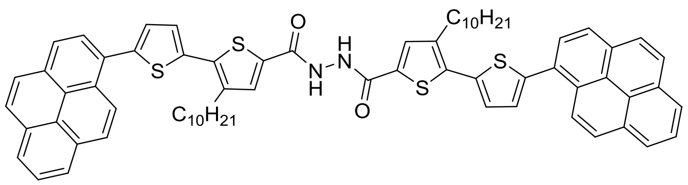

Yield: 0.64 g (65 %); yellow solid; m.p.= 173-175^{0}C (EtOAc), Rf= 0.31($CHCl_3$).

IR (KBr). ν cm^{-1}: 1591 (C=O), 3172 (NH).

^{1}H NMR (400 MHz, DMSO-d$_6$): 0.73-0.76 (m., 6H, 2CH_3); 1.15-1.44 (m., 28H, 14CH_2,); 1.70-1.75 (m., 4H, 2$CH_2C\underline{H}_2C_8H_{17}$); 2.87 (t., ^{3}J=7.8, 4H, 2$C\underline{H}_2C_9H_{19}$); 7.50 (d., ^{3}J=3.6, 2H, 2Th-3'-H); 7.51 (d., ^{3}J=3.6, 2H, 2Th-4'-H); 7.85 (s., 2H, 2Th-4-H); 8.20-8.35 (m., 16H, 2 pyrenyl-2,3,4,5,6,7,8,10-H); 8.52(d., ^{3}J=9.2, 2H, 2 pyrenyl-9-H); 10.44 (s., 2H, 2NH).

^{13}C NMR (100 MHz, δ, ppm): 13.33 ($\underline{C}H_3$); 21.54, 28.21, 28.32, 28.51, 28.55, 29.38, 30.82 ($\underline{C}H_2$); 123.65, 124.05, 127.93, 130.10, 130.58, 133.00, 142.28 (1,3a,5a,8a,10a,b,c-pyrenyl); 123.76 (4'-Th); 124.60 (3'-Th); 125.00, 125.42, 126.25, 126.92, 127.46, 127.69, 127.79, 128.06, 128.71 (2,3,4,5,6,7,8,9,10-pyrenyl); 127.88 (2-Th); 129.95 (4-Th); 130.72 (5-Th); 134.77 (2'-Th); 135.13 (5'-Th); 137.10 (3-Th); 158.88 (CO).

Elemental analysis. Calculated for $C_{70}H_{68}N_2O_2S_4$: C, 76.60%; H, 6.25%; N, 2.55%. found: C, 76.51%; H, 6.32%; N, 2.50%.

Synthesis of _N,N'_-bis(3-decyl-2,2':5',2''-terthiophen-5-carbonyl)hydrazine (14c)

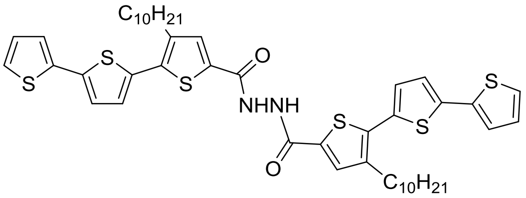

Carbohydrazide **13c** (0.9 mmol) was added to a solution of carboxylic acid **12c** (0.9 mmol) and DCC (*N*,*N*'-dicyclohexylcarbodiimide, 0.18 g, 0.9 mmol) in anhydrous THF (5 mL) and the mixture was stirred at rt for 3–6 h. Afterwards, acetic acid (100 μL, 10%) was added and the precipitate of *N*,*N*'-

dicyclohexylurea was filtered off and washed with THF (15 mL). The solvent was then removed under reduced pressure and the resulting crude product was purified by crystallization from methanol or ethyl acetate.

Yield: 0.47 g (61%); M.p.=183-185^{0}C (methanol), yellow solid. Rf=0.26 ($CHCl_3$).

IR (KBr). ν cm^{-1}: 1629 (C=O), 3206-3441 (NH).

^{1}H NMR (400 MHz, DMSO-d6): δ =0.81-0.84 (m, 6H, 2CH_3), 1.22-1.35 (m, 28H, 14CH_2), 1.63-1.66 (m, 4H, 2Th$CH_2C\underline{H}_2C_8H_{17}$), 2.76 (t, ^3J=7.4, 4H, 2Th$C\underline{H}_2C_9H_{19}$), 7.10 (d.d, ^3J=3.7, ^3J=4.9, 2H, 2Th-4″-H), 7.23 (d., ^3J=3.2, 2H, 2Th-3′-H), 7.30 (d., ^3J=3.7, 2H, 2Th-3″-H), 7.34 (d., ^3J=3.2, 2H, 2Th-4′-H), 7.52 (d., ^3J=4.9, 2H, 2Th-5″-H), 7.79 (s, 2H, 2Th-4-H), 10.44 (s., 2H, 2N$\underline{H}$).

^{13}C NMR (100 MHz, δ, ppm): 13.49 (CH_3), 21.70, 28.31, 28.36, 28.39, 28.46, 28.58, 28.64, 29.34, 30.94 (9CH_2), 124.28 (4′-Th), 124.38 (3′-Th), 125.67 (5’’-Th), 127.73 (3’’-Th), 128.07 (4″-Th), 131.66 (4-Th), 132.88 (2-Th), 134.13 (5’-Th), 134.52 (2’-Th), 135.41 (5-Th), 136.74 (2’’-Th), 137.30 (3-Th), 160.24 (C=O).

Elemental analysis. Calculated for $C_{46}H_{56}N_2O_2S_6$: C, 64.15%; H, 6.55 %; N, 3.25%. found: C, 64.28%; H, 6.60%; N, 3.31%.

Synthesis of 5'-aryl-substituted 2,5-bis(3-decyl-2,2'-bithiophen-5-yl)-1,3,4-oxadiazoles (15d–g) and 2,5-bis(3-decyl-2,2':5',2''-terthiophen-5-yl)-1,3,4-oxadiazole (15c)

A mixture of **14c**–**g** (0.5 mmol) and 3 mL phosphoryl chloride was heated to 80–90 °C and stirred at this temperature under inert atmosphere for 6 h, then cooled to rt. The excess of phosphoryl chloride was removed under reduced pressure and 30 mL of ice water were added. The product was extracted into $CHCl_3$ (3 × 20 mL), washed with a saturated solution of sodium chloride (30 mL) and$NaHCO_3$ and dried over Na_2SO_4.

Then the solvent was evaporated under reduced pressure and the crude product was purified by column chromatography or crystallized.

2,5-Bis(3-decyl-5'-phenyl-2,2'-bithiophen-5-yl)-1,3,4-oxadiazole (15d)

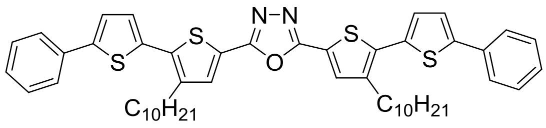

Yield: 0.35 g (84 %); yellow solid; m.p.= 114-116 ^{0}C (benzene), Rf= 0.36 (silica gel, benzene).

IR (KBr). ν cm^{-1}: 1578 (C=N).

^{1}H NMR (400 MHz, CDCl$_3$): 0.85-0.89 (m., 6H, 2CH$_3$); 1.25-1.46 (m., 28H, 14CH$_2$,); 1.68-1.76 (m., 4H, 2CH$_2$C<u>H</u>$_2$C$_8$H$_{17}$); 2.84 (t., ^{3}J=7.8, 4H, 2C<u>H</u>$_2$C$_9$H$_{19}$); 7.19 (d., ^{3}J=3.9, 2H, 2Th-3'-H); 7.29 (d., ^{3}J=3.9, 2H, 2Th-4'-H); 7.31-7.41 (m., 6H, 2Ph-3,4,5-H); 7.61-7.63 (m., 4H, 2Ph-2,6-H); 7.63 (s., 2H, 2Th-4-H).

^{13}C NMR (100 MHz, δ, ppm): 14.11 (<u>C</u>H$_3$); 22.70, 29.35, 29.43, 29.45, 29.53, 29.61, 29.64, 30.43, 31.93 (CH$_2$); 121.83 (2-Th); 123.62 (4′-Th); 125.79 (2,6-Ph); 127.87 (3′-Th); 127.96 (4-Ph); 129.03 (3,5-Ph); 132.47 (4-Th); 133.78 (5-Th); 134.11 (1-Ph); 136.05(3-Th); 140.46 (5'-Th); 145.50 (2'-Th); 160.00 (2,5-Oxadiazole).

Elemental analysis. Calculated for $C_{50}H_{58}N_2OS_4$: C, 72.24 %; H, 7.03 %; N, 3.37%. found: C, 72.48%; H, 7.28 %; N, 3.41%.

2,5-Bis(3-decyl-5'-(naphthalen-1-yl)-2,2'-bithiophen-5-yl)-1,3,4-oxadiazole (15e)

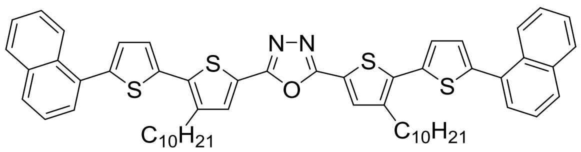

Yield: 0.34 g (73 %); yellow solid; m.p.= 94-95 ^{0}C (benzene), Rf= 0.25 (silica gel, benzene).

IR (KBr). ν cm^{-1}: 1579 (C=N).

^{1}H NMR (400 MHz, CDCl$_3$): 0.86 (t., ^{3}J=6.7, 6H, 2CH$_3$); 1.25-1.43 (m., 28H, 14CH$_2$,); 1.71-1.78 (m, 4H, 2CH$_2$C<u>H</u>$_2$C$_8$H$_{17}$); 2.88 (t., ^{3}J=7.8, 4H, 2C<u>H</u>$_2$C$_9$H$_{19}$); 7.24 (d., ^{3}J=3.7,

2H, 2Th-3'-H); 7.31 (d., ^3J=3.7, 2H, Th-4'-H); 7.48-7.62 (m., 8H, 2naphtyl-2,3,6,7-H); 7.67(s., 2H, 2Th-4-H); 7.86-7.92 (m., 4H, 2naphtyl -5,8-H); 8.28-8.30 (m., 2H, 2naphtyl -4-H).

^{13}C NMR (100 MHz, δ, ppm): 14.07 (<u>C</u>H$_3$); 22.67, 29.33, 29.47, 29.49, 29.56, 29.61, 29.63, 30.52, 31.92 (CH$_2$); 122.04 (2-Th); 125.29 (4'-Th); 125.61 (3'-Th); 126.20, 126.71, 127.22, 127.99, 128.20, 128.49, 128.87 (2,3,4,5,6,7,8-naphtyl); 131.74 (5-Th); 131.75 (4a-naphtyl); 132.52 (4-Th); 134.05 (3-Th); 135.12 (5'-Th); 136.07 (8a-naphtyl); 140.55 (1-naphtyl); 143.29 (2'-Th); 160.10 (2,5-Oxadiazole).

Elemental analysis. Calculated for $C_{58}H_{62}N_2OS_4$: C, 74.80%; H, 6.71%; N, 3.01%. found: C, 74.77%; H, 6.70%; N, 3.05%.

2,5-Bis(5'-(anthracen-9-yl)-3-decyl-2,2'-bithiophen-5-yl)-1,3,4-oxadiazole (15f)

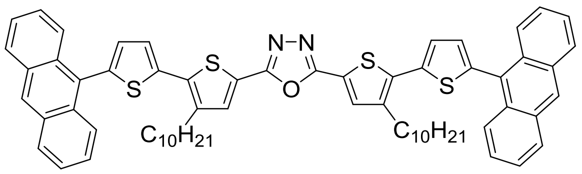

Yield: 0.36 g (70 %); yellow solid; m.p.= 93-95 ^{0}C (hexane/CH$_2$Cl$_2$, 1/2), Rf= 0.28 (silica gel, hexane/CH$_2$Cl$_2$, 1/2).

IR (KBr). ν cm^{-1}: 1570 (C=N).

^{1}H NMR (400 MHz, CDCl$_3$): 0.84 (t., ^3J=6.7, 6H, 2CH$_3$); 1.22-1.43 (m., 28H, 14CH$_2$,); 1.72-1.79 (m., 4H, 2CH$_2$C<u>H</u>$_2$C$_8$H$_{17}$); 2.90 (t., ^3J=7.6, 4H, 2C<u>H</u>$_2$C$_9$H$_{19}$); 7.17 (d., ^3J=3.7, 2H, 2Th-3'-H); 7.44-7.50 (m., 10H, 2antryl-2,3,6,7-H, 2Th-4'-H); 7.69 (s., 2H, 2Th-4-H); 7.96 (d., ^3J=8.0, 4H, 2antryl-1,8-H); 8.03 (d., ^3J=7.2, 4H, 2antryl-4,5-H); 8.54 (s., 2H, 2antryl-10-H).

^{13}C NMR (100 MHz, δ, ppm): 14.06 (<u>C</u>H$_3$); 22.65, 29.30, 29.46, 29.55, 29.59, 30.49, 31.88 (CH$_2$); 122.04 (2-Th); 125.36 (4'-Th); 126.40 (3'-Th); 126.20, 127.04, 128.41, 128.43, 130.11 (2,3,4,5,6,7,8,10-antryl); 127.68 (5-Th); 131.31, 131.82, 136.32 (4a,10a,9,8a,9a-antryl); 132.58 (4-Th); 136.07 (3-Th); 140.56 (5'-Th); 140.5 (2'-Th); 160.13 (2,5-Oxadiazole).

Elemental analysis. Calculated for $C_{66}H_{66}N_2OS_4$: C, 76.85 %; H, 6.45%; N, 2.72%. found: C, 76.81%; H, 6.50%; N, 2.70%.

2,5-Bis(3-decyl-5'-(pyren-1-yl)-2,2'-bithiophen-5-yl)-1,3,4-oxadiazole (15g)

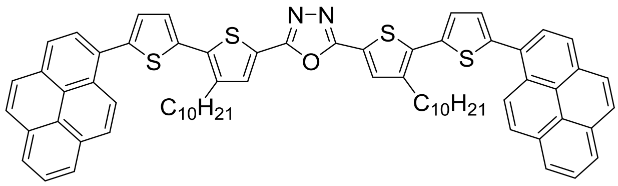

Yield: 0.35 g (65%); orange solid; m.p.= 96-97 ^{0}C (crystallized from toluene), Rf= 0.18 (silica gel, hexane/CH_2Cl_2, 1/2).

IR (KBr). ν cm^{-1}: 1580 (C=N).

^{1}H NMR (400 MHz, $CDCl_3$): 0.84 (t., ^3J=6.8, 6H, $2CH_3$); 1.24-1.48 (m., 28H, $14CH_2$,); 1.73-1.81 (m., 4H, $2CH_2C\underline{H}_2C_8H_{17}$); 2.91 (t., ^3J=7.8, 4H, $2C\underline{H}_2C_9H_{19}$); 7.36 (d., ^3J=3.5, 2H, 2Th-3'-H); 7.38 (d., ^3J=3.5, 2H, 2Th-4'-H); 7.67 (s., 2H, 2Th-4-H); 8.00-8.21 (m., 16H, 2 pyrenyl-2,3,4,5,6,7,8,10-H); 8.55 (d., ^3J=9.2, 2H, 2 pyrenyl-9-H).

^{13}C NMR (100 MHz, δ, ppm): 14.06 ($\underline{C}H_3$); 22.67, 29.35, 29.49, 29.54, 29.59, 29.63, 29.64, 30.53, 31.92 ($\underline{C}H_2$), 122.05(10c-pyrenyl); 124.68 (4'-Th); 124.77 (3'-Th); 124.83 (2-Th); 125.18 (10b-pyrenyl); 125.24, 125.55, 126.27, 127.35, 127.39, 128.05, 128.24, 128.51 (2,3,4,5,6,7,8,9,10- pyrenyl); 128.96, 129.10, 131.36, 131.54(3a,5a,8a,10a-pyrenyl); 131.01(5-Th); 132.55 (4-Th); 135.70 (3-Th); 136.08 (5'-Th); 140.60 (2'-Th); 144.01(1-pyrenyl); 160.10 (2,5-Oxadiazole).

Elemental analysis. Calculated for $C_{70}H_{66}N_2OS_4$: C, 77.88%; H, 6.16%; N, 2.59%. found: C, 77.82%; H, 6.18%; N, 2.60%.

2,5-Bis(3-decyl-2,2':5',2''-terthiophen-5-yl)-1,3,4-oxadiazole (15c)

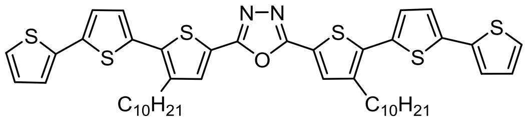

Yield: 0.40 g (94%); M.p.= 98-100 ^{0}C (benzene), orang solid. Rf=0.51 (benzene).

IR (KBr). ν cm^{-1}: 1578 (C=N).

^{1}H NMR (400 MHz,$CDCl_3$): δ =0.87 (t., ^3J=6.7 Hz, 6H, $2CH_3$), 1.27-1.45 (m, 28H, $14CH_2$), 1.67-1.75 (m, 4H, $2ThCH_2C\underline{H}_2C_8H_{17}$), 2.81 (t, ^3J=7.8 Hz, 4H, $2ThC\underline{H}_2C_9H_{19}$), 7.03 (d.d, ^3J=3.7 Hz, ^3J=4.7 Hz, 2H, 2Th-4″-H), 7.13 (d., ^3J=3.8 Hz, 2H, 2Th-3′-H), 7.14 (d., ^3J=3.8 Hz, 2H, 2Th-4′-H), 7.20 (d., ^3J=3.7 Hz, 2H, 2Th-3″-H), 7.24 (d., ^3J=4.7 Hz, 2H, 2Th-5″-H), 7.62 (s, 2H, 2Th-4-H).

^{13}C NMR (100 MHz, δ, ppm): 14.09 (CH_3), 22.70, 29.35, 29.45, 29.53, 29.61, 29.64, 30.43, 31.94 ($9CH_2$), 122.08 (2-Th), 124.18 (4′-Th), 124.20 (3′-Th), 125.00 (5’’-Th), 127.65 (3’’-Th), 127.98 (4″-Th), 132.47 (4-Th), 133.64 (5-Th), 135.78 (5’-Th), 136.77 (2’’-Th), 138.68 (3-Th), 140.66 (2’-Th), 160.02 (2,5-Oxadiazole).

Elemental analysis. Calculated for $C_{46}H_{54}N_2OS_6$: C, 65.52%; H, 6.45%; N, 1.90%. found: C, 65.42%; H, 6.40%; N, 1.88%.

Figure S1: Normalized absorption and photoluminescence spectra of dichloromethane solutions of a) **15c**, b) **15b**, c) **15d**, d) **15e**, e) **15f** and f) **15g**.

a)

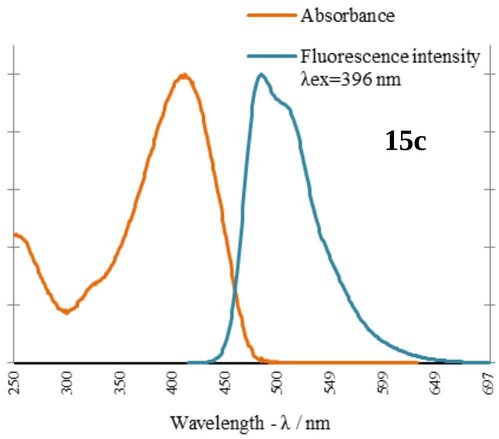

b)

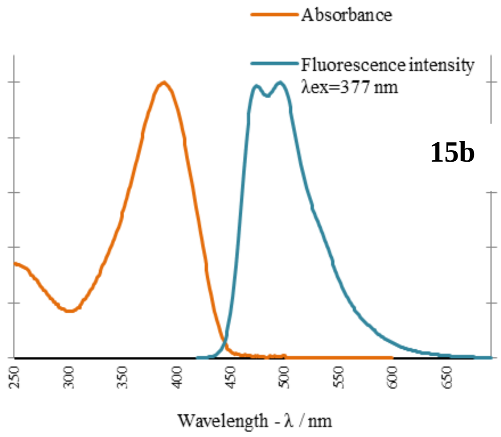

c)

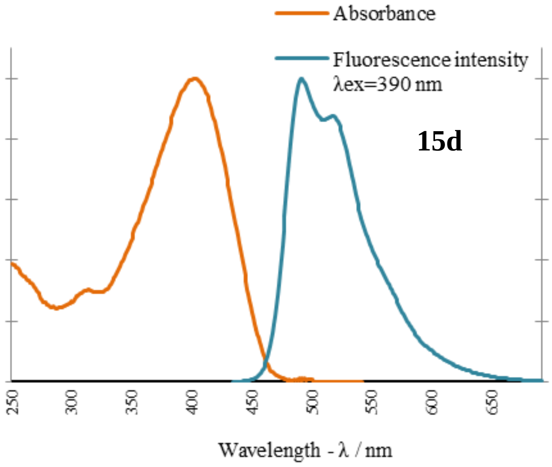

d)

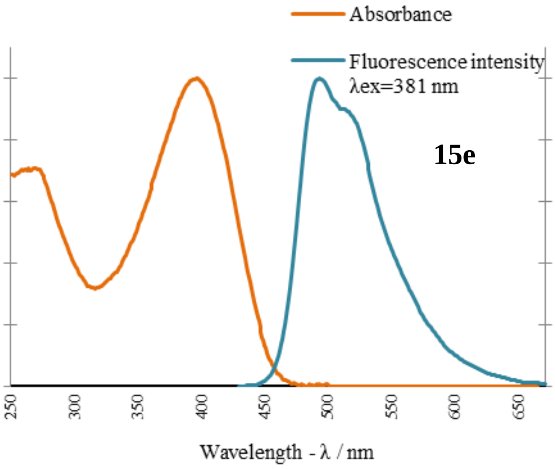

e)

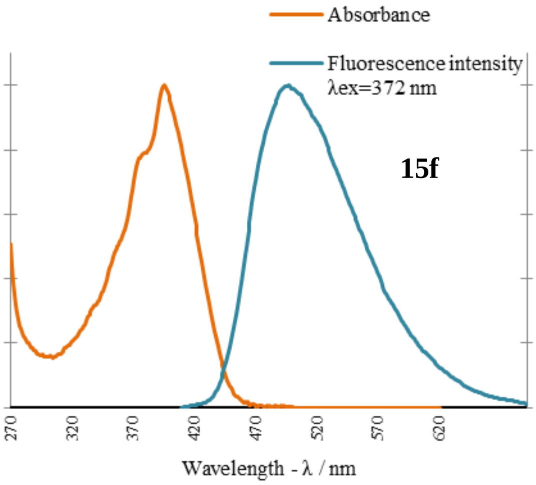

f)

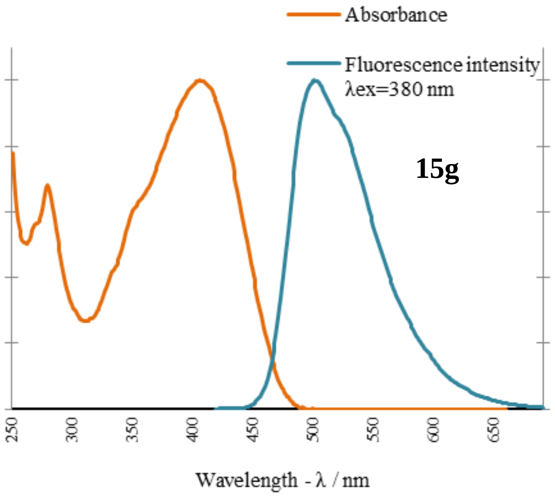

Figure S2: Geometrical structures of compounds **15c–f.**

2,5-Bis(3-methyl-2,2':5',2''-terthiophen-5-yl)-1,3,4-oxadiazole (**15c**). B3LYP/Def2-SVPD/IEFPCM(DCM)/IEFPCM(DCM)

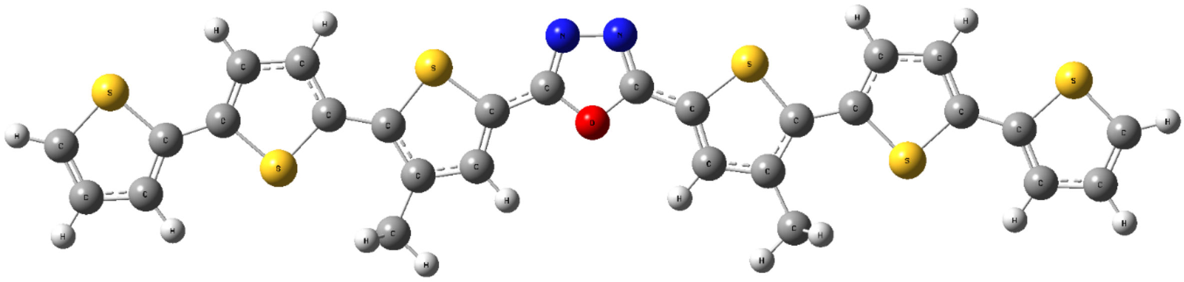

E = -3650.180107 Hartree

2,5-Bis(3-methyl-5'-phenyl-2,2'-bithiophen-5-yl)-1,3,4-oxadiazole (**15d**). B3LYP/Def2-SVPD/IEFPCM(DCM)/IEFPCM(DCM)

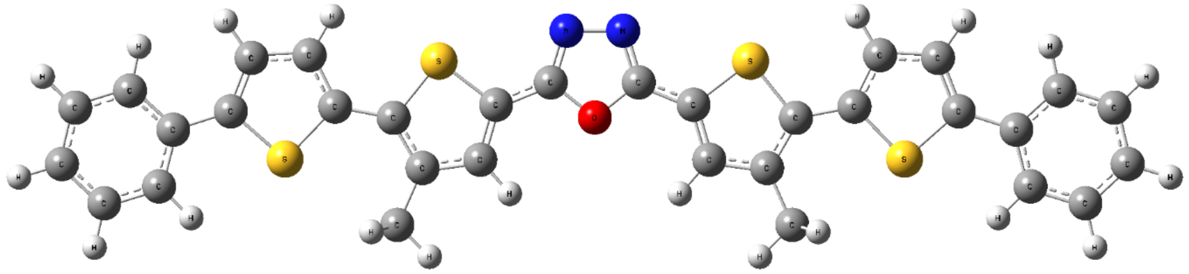

E = -3008.769462 Hartree

2,5-Bis(3-methyl-5'-(naphthalen-1-yl)-2,2'-bithiophen-5-yl)-1,3,4-oxadiazole (**15e**). B3LYP/Def2-SVPD/IEFPCM(DCM)

SS1 conformer.

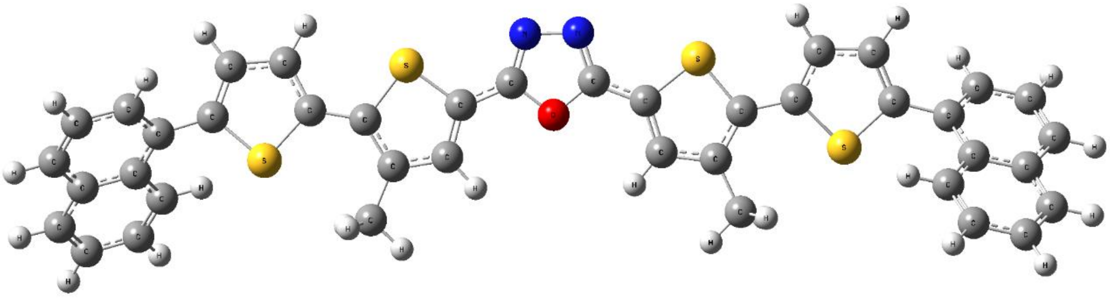

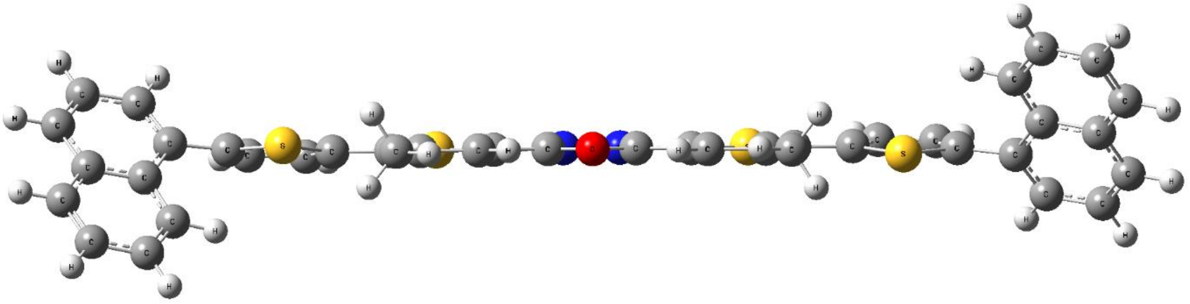

E = -3315.841542 Hartree

SS2 conformer.

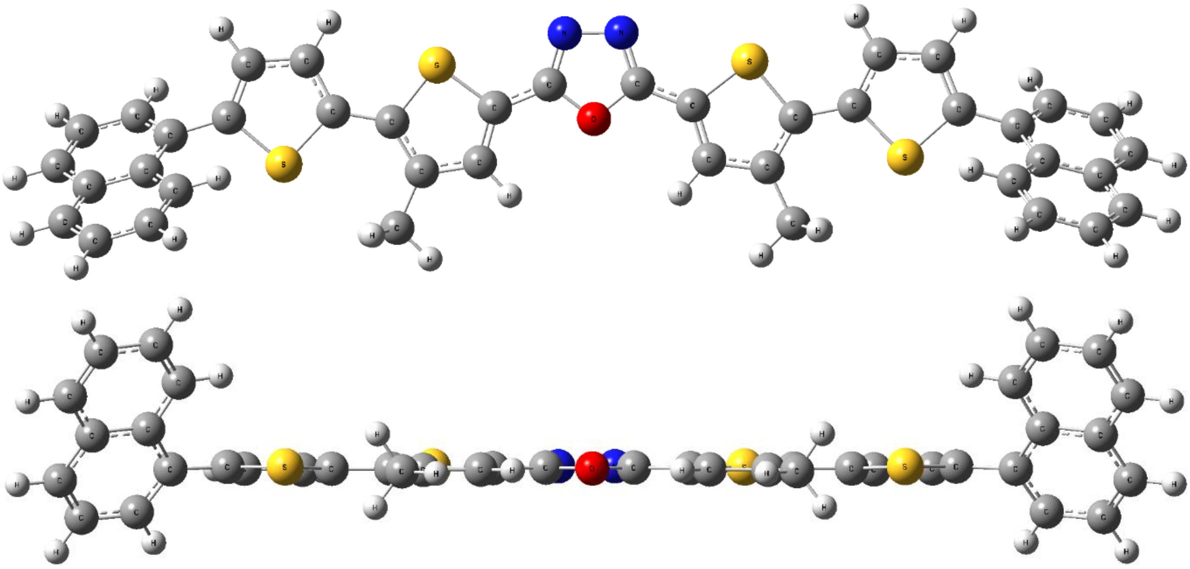

E = -3315.841577 Hartree

AS conformer.

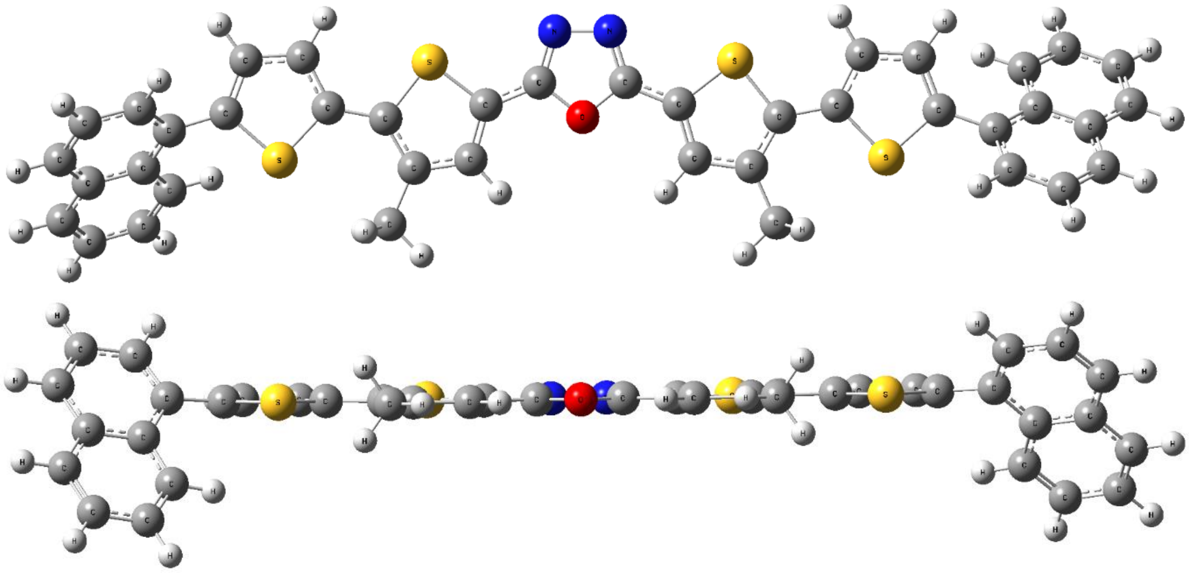

E = -3315.842346 Hartree

AA1 conformer.

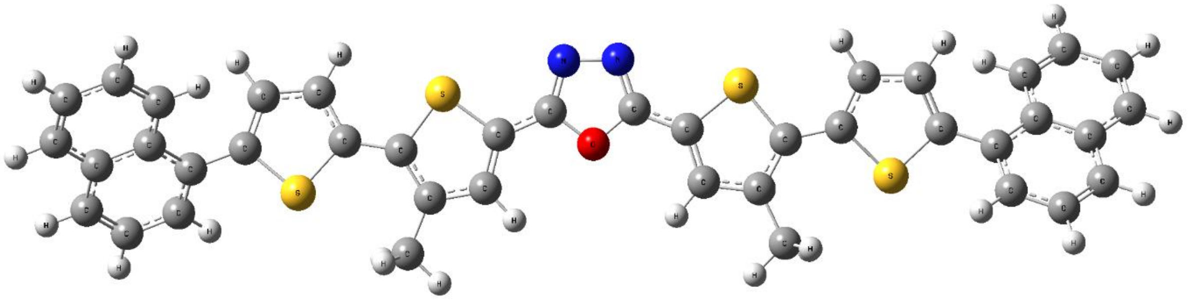

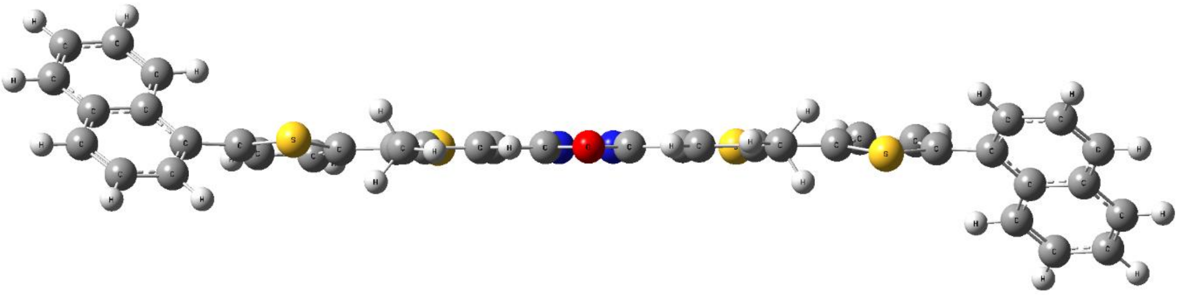

E =-3315.843073 Hartree

AA2 conformer.

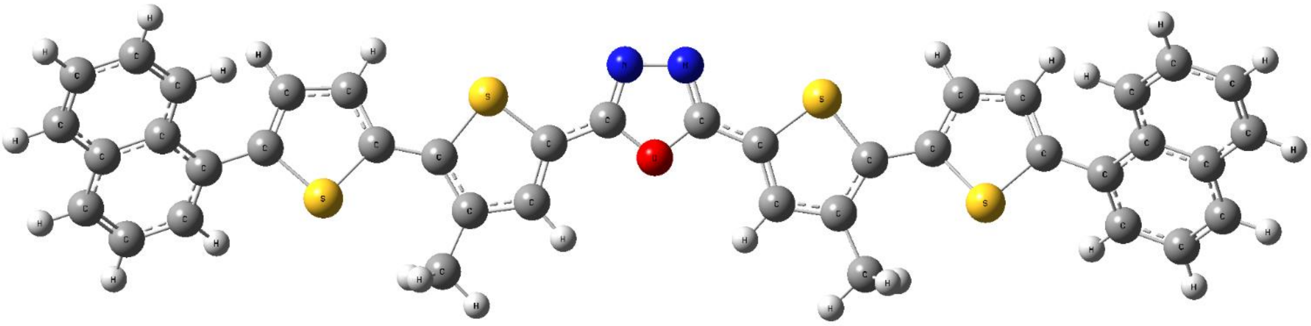

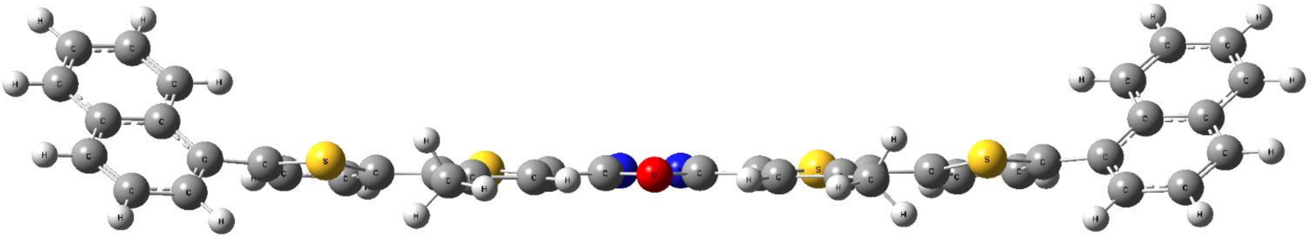

E = -3315.843255 Hartree

2,5-Bis(5'-(anthracen-9-yl)-3-methyl-2,2'-bithiophen-5-yl)-1,3,4-oxadiazole (**15f**).
B3LYP/Def2-SVPD/IEFPCM(DCM)

Conformer **A**

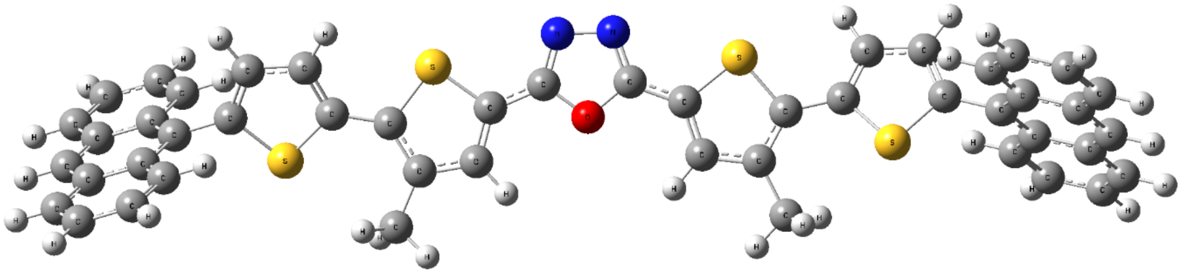

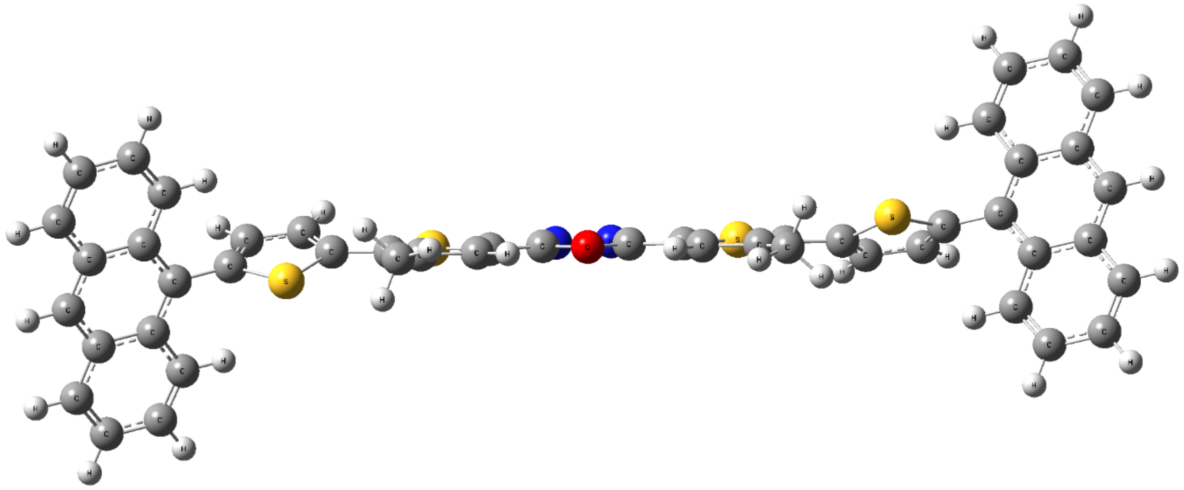

E = -3622.908454 Hartree

Conformer **S**

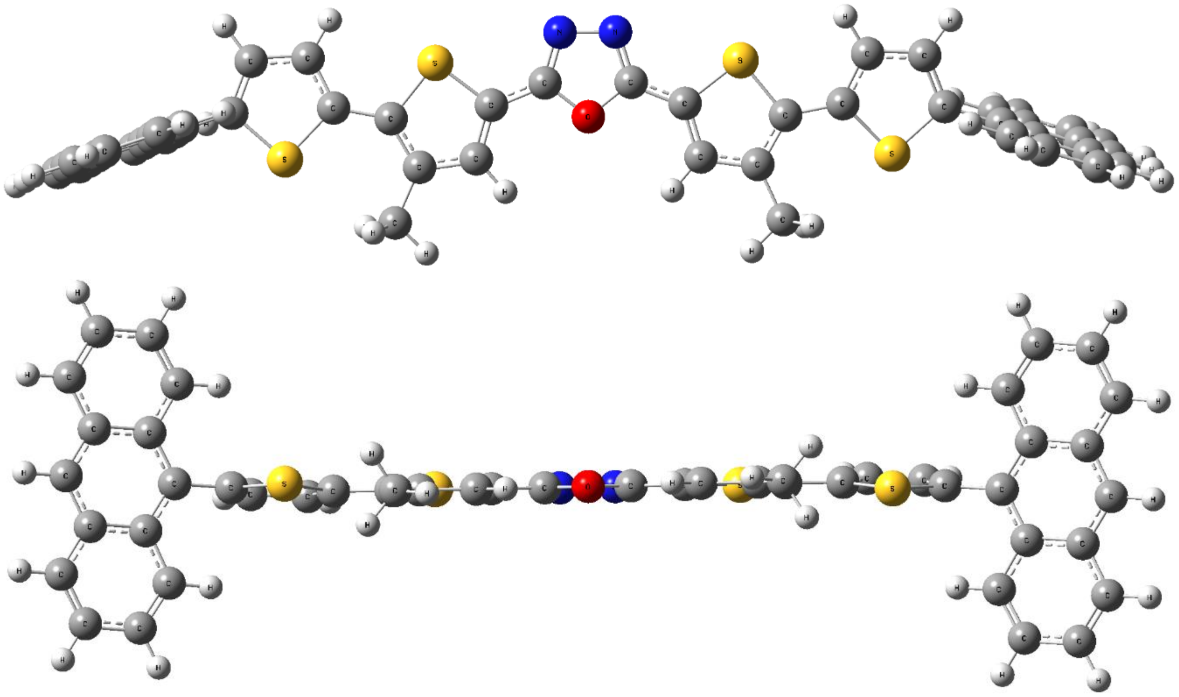

E = -3622.90866254 Hartree

Cartesian coordinate for studied molecules.

a) Cartesian coordinates for **15c** obtained with B3LYP functional for ground states.

Ground state. DCM.
2,5-Bis(3-methyl-2,2':5',2"-terthiophen-5-yl)-1,3,4-oxadiazole
IEFPCM-B3LYP/Def2-SVPD/IEFPCM(DCM)

Atom	X	Y	Z
C	1.069295	-0.857461	0.000182
C	-1.069295	-0.857461	-0.000253
O	0.000000	-0.012207	-0.000037
C	-2.400980	-0.319809	-0.000502
C	-2.785690	1.005667	-0.000825
S	-3.786816	-1.375445	-0.000400
C	-4.192282	1.203822	-0.000957

H	-2.070687	1.826563	-0.000946
C	-4.892082	-0.006598	-0.000712
C	2.400980	-0.319809	0.000441
C	2.785690	1.005667	0.000767
S	3.786816	-1.375445	0.000343
C	4.192282	1.203822	0.000919
H	2.070687	1.826563	0.000886
C	4.892082	-0.006598	0.000679
C	-6.313638	-0.280276	-0.000585
C	-6.934878	-1.521637	-0.000198
S	-7.534658	0.988368	-0.000788
C	-8.346109	-1.460468	-0.000038
H	-6.383891	-2.460985	0.000005
C	-8.848333	-0.169620	-0.000295
H	-8.980676	-2.345684	0.000297
C	6.313638	-0.280275	0.000570
C	6.934878	-1.521637	0.000131
S	7.534658	0.988368	0.000880
C	8.346109	-1.460468	-0.000006
H	6.383891	-2.460985	-0.000140
C	8.848333	-0.169620	0.000322
H	8.980676	-2.345684	-0.000390
N	0.688274	-2.106299	0.000108
N	-0.688274	-2.106299	-0.000180
C	4.806414	2.577041	0.001269
H	5.436803	2.743154	0.887907
H	5.436931	2.743550	-0.885204
H	4.022059	3.342752	0.001385
C	-4.806414	2.577041	-0.001289
H	-5.436835	2.743150	-0.887905
H	-5.436899	2.743553	0.885206
H	-4.022059	3.342752	-0.001436
C	-10.227504	0.276460	-0.000141
C	-10.728759	1.566704	-0.000483
S	-11.548027	-0.882040	0.000648
C	-12.151859	1.623633	-0.000093
H	-10.092799	2.451258	-0.000989
C	-12.735121	0.381677	0.000535
H	-12.720207	2.553148	-0.000269
H	-13.792808	0.130233	0.000936
C	10.227504	0.276460	0.000189
C	10.728759	1.566704	0.000529
S	11.548027	-0.882040	-0.000566
C	12.151859	1.623633	0.000154
H	10.092799	2.451258	0.001013
C	12.735122	0.381677	-0.000460
H	12.720207	2.553147	0.000325
H	13.792808	0.130233	-0.000852

b) Cartesian coordinates for **15d** obtained with B3LYP functional for ground states.

Ground state. DCM.
2,5-Bis(3-methyl-5'-phenyl-2,2'-bithiophen-5-yl)-1,3,4-oxadiazole
IEFPCM-B3LYP/Def2-SVPD/IEFPCM(DCM)

Atom	X	Y	Z
C	-1.069293	0.961728	-0.000048
C	1.069294	0.961726	-0.000105
O	0.000000	0.116577	-0.000115
C	2.400782	0.423150	-0.000136

C	2.783901	-0.902717	-0.000168
S	3.787860	1.476926	-0.000124
C	4.190471	-1.102470	-0.000202
H	2.068013	-1.722830	-0.000176
C	4.891337	0.106963	-0.000205
C	-2.400782	0.423153	-0.000045
C	-2.783904	-0.902713	-0.000098
S	-3.787859	1.476931	0.000000
C	-4.190474	-1.102464	-0.000075
H	-2.068017	-1.722828	-0.000142
C	-4.891338	0.106970	0.000002
C	6.313879	0.379390	-0.000258
C	6.936415	1.619856	-0.000533
S	7.533037	-0.886520	0.000054
C	8.347876	1.554678	-0.000436
H	6.387960	2.560672	-0.000800
C	8.852025	0.265377	-0.000086
H	8.976959	2.442548	-0.000620
C	-6.313880	0.379397	0.000062
C	-6.936417	1.619862	0.000263
S	-7.533037	-0.886515	-0.000224
C	-8.347878	1.554682	0.000292
H	-6.387963	2.560679	0.000438
C	-8.852025	0.265381	0.000120
H	-8.976963	2.442550	0.000490
C	10.252439	-0.178679	0.000123
C	11.302570	0.764687	0.000350
C	10.596192	-1.546960	0.000105
C	12.634795	0.354655	0.000543
H	11.078033	1.830482	0.000381
C	11.930341	-1.953943	0.000310
H	9.813021	-2.305796	-0.000087
C	12.958883	-1.006672	0.000525
H	13.426579	1.105336	0.000716
H	12.166626	-3.019178	0.000291
H	14.001976	-1.325337	0.000679
C	-10.252438	-0.178680	0.000140
C	-10.596187	-1.546962	0.000129
C	-11.302573	0.764683	0.000159
C	-11.930335	-1.953949	0.000159
H	-9.813014	-2.305795	0.000116
C	-12.634797	0.354646	0.000192
H	-11.078039	1.830479	0.000153
C	-12.958880	-1.006682	0.000185
H	-12.166616	-3.019185	0.000157
H	-13.426582	1.105326	0.000212
H	-14.001972	-1.325350	0.000204
N	-0.688370	2.210524	-0.000060
N	0.688372	2.210523	-0.000039
C	-4.802655	-2.476522	-0.000115
H	-5.433401	-2.643211	0.886123
H	-5.433325	-2.643191	-0.886411
H	-4.017263	-3.241155	-0.000092
C	4.802650	-2.476530	-0.000243
H	5.433434	-2.643148	-0.886467
H	5.433281	-2.643272	0.886067
H	4.017256	-3.241161	-0.000359

c) Cartesian coordinates for **15e** obtained with B3LYP functional for ground states.

Ground state. DCM.
2,5-Bis(3-methyl-5'-(naphthalen-1-yl)-2,2'-bithiophen-5-yl)-1,3,4-oxadiazole. **Conformer SS1.**
IEFPCM-B3LYP/Def2-SVPD/IEFPCM(DCM)

Atom	X	Y	Z
C	-1.068962	-1.331300	-0.027965
C	1.068960	-1.331302	0.028048
O	-0.000002	-0.486139	0.000091
C	2.399824	-0.791761	0.064301
C	2.780944	0.534321	0.082279
S	3.788104	-1.843588	0.094810
C	4.187290	0.735468	0.119335
H	2.064218	1.353595	0.067394
C	4.888775	-0.472632	0.124434
C	-2.399827	-0.791759	-0.064155
C	-2.780955	0.534322	-0.081967
S	-3.788102	-1.843591	-0.094784
C	-4.187301	0.735467	-0.119027
H	-2.064234	1.353599	-0.066967
C	-4.888778	-0.472637	-0.124297
C	6.313541	-0.742728	0.153607
C	6.941209	-1.974205	0.041419
S	7.523107	0.515847	0.363218
C	8.353802	-1.907712	0.121912
H	6.398018	-2.906011	-0.110176
C	8.845187	-0.628354	0.288502
H	9.004739	-2.777429	0.043310
C	-6.313541	-0.742737	-0.153539
C	-6.941212	-1.974219	-0.041424
S	-7.523100	0.515846	-0.363154
C	-8.353801	-1.907727	-0.121997
H	-6.398027	-2.906028	0.110178
C	-8.845179	-0.628364	-0.288572
H	-9.004742	-2.777446	-0.043456
N	-0.688302	-2.579948	-0.018180
N	0.688301	-2.579949	0.018125
C	-4.798517	2.109808	-0.144512
H	-5.357087	2.291447	-1.075233
H	-5.497946	2.261883	0.690989
H	-4.015938	2.874011	-0.071579
C	4.798497	2.109811	0.144979
H	5.357131	2.291321	1.075688
H	5.497868	2.262014	-0.690547
H	4.015908	2.874018	0.072209
C	10.253370	-0.233338	0.494611
C	10.904262	0.793326	-0.280260
C	10.976218	-0.901071	1.476888
C	10.269339	1.485595	-1.351460
C	12.274297	1.119297	0.016722
C	12.327719	-0.587947	1.746789
H	10.478185	-1.668790	2.069213
C	10.932078	2.463094	-2.065588
H	9.245838	1.232515	-1.618847
C	12.925249	2.137446	-0.736511
C	12.961781	0.410310	1.040471
H	12.857626	-1.131410	2.530371
C	12.270996	2.801933	-1.751203
H	10.421948	2.974486	-2.883325

Ground state. DCM.
2,5-Bis(3-methyl-5'-(naphthalen-1-yl)-2,2'-bithiophen-5-yl)-1,3,4-oxadiazole. **Conformer SS2.**
IEFPCM-B3LYP/Def2-SVPD/IEFPCM(DCM)

Atom	X	Y	Z
C	1.069348	-1.284458	-0.369040
C	-1.069330	-1.284462	-0.369082
O	-0.000001	-0.564249	0.072921
C	-2.400527	-0.824231	-0.086563
C	-2.781097	0.306850	0.606003
S	-3.789760	-1.720540	-0.635530
C	-4.187845	0.477878	0.713287
H	-2.063533	1.004874	1.033684
C	-4.890158	-0.552754	0.083789
C	2.400532	-0.824214	-0.086481
C	2.781073	0.306910	0.606031
S	3.789789	-1.720551	-0.635342
C	4.187817	0.477936	0.713373
H	2.063491	1.004961	1.033638
C	4.890155	-0.552745	0.083988
C	-6.315527	-0.785554	-0.050548
C	-6.940512	-1.866294	-0.654245
S	-7.530876	0.336168	0.547172
C	-8.355142	-1.794579	-0.637070
H	-6.393915	-2.692349	-1.107205
C	-8.850101	-0.657626	-0.030770
H	-9.003460	-2.554828	-1.070730
C	6.315529	-0.785585	-0.050233
C	6.940532	-1.866502	-0.653591
S	7.530855	0.336327	0.547169
C	8.355160	-1.794773	-0.636409
H	6.393949	-2.692703	-1.106299
C	8.850097	-0.657637	-0.030436
H	9.003494	-2.555148	-1.069818
N	0.688603	-2.348595	-1.022288
N	-0.688556	-2.348594	-1.022322
C	4.799296	1.650016	1.431243
H	5.457169	1.328607	2.252508
H	5.402552	2.273943	0.754376
H	4.014379	2.284563	1.858861
C	-4.799352	1.649919	1.431194
H	-5.402664	2.273826	0.754359
H	-5.457179	1.328468	2.252480
H	-4.014449	2.284495	1.858794
C	-10.266564	-0.326820	0.227764
C	-10.862600	0.925266	-0.165458
C	-11.051468	-1.280817	0.864903
C	-10.157512	1.936070	-0.880115
C	-12.247218	1.160560	0.148729
C	-12.415702	-1.047801	1.152267
H	-10.594509	-2.222296	1.170029
C	-10.769685	3.120302	-1.236967
H	-9.119760	1.767250	-1.159157
C	-12.844973	2.396508	-0.229396
C	-13.000009	0.152388	0.812267
H	-12.996122	-1.819605	1.659785
C	-12.124869	3.360237	-0.901594
H	-10.206363	3.875275	-1.787524

H	13.962037	2.379865	-0.497062
H	14.000156	0.668372	1.253807
H	12.784520	3.578488	-2.319865
C	-10.253351	-0.233341	-0.494740
C	-10.904268	0.793316	0.280113
C	-10.976156	-0.901049	-1.477063
C	-10.269383	1.485566	1.351347
C	-12.274287	1.119299	-0.016919
C	-12.327644	-0.587913	-1.747021
H	-10.478098	-1.668757	-2.069381
C	-10.932142	2.463059	2.065461
H	-9.245891	1.232478	1.618765
C	-12.925264	2.137437	0.736303
C	-12.961731	0.410336	-1.040711
H	-12.857518	-1.131358	-2.530638
C	-12.271047	2.801906	1.751031
H	-10.422043	2.974441	2.883225
H	-13.962042	2.379864	0.496819
H	-14.000094	0.668412	-1.254090
H	-12.784592	3.578453	2.319683

Ground state. DCM.
2,5-Bis(3-methyl-5'-(naphthalen-1-yl)-2,2'-bithiophen-5-yl)-1,3,4-oxadiazole. **Conformer AS.**
IEFPCM-B3LYP/Def2-SVPD/IEFPCM(DCM)

Atom	X	Y	Z
C	-1.092877	-1.128372	-0.064176
C	1.043883	-1.039037	-0.087013
O	-0.060732	-0.248799	-0.200641
C	2.350484	-0.450490	-0.186050
C	2.673305	0.875024	-0.392997
S	3.783786	-1.431318	-0.050216
C	4.069690	1.132279	-0.448097
H	1.920921	1.653741	-0.505197
C	4.824029	-0.031824	-0.279833
C	-2.445884	-0.650828	-0.135063
C	-2.882497	0.644723	-0.321468
S	-3.789252	-1.750068	0.013331
C	-4.296446	0.784008	-0.350919
H	-2.200604	1.485488	-0.436249
C	-4.946946	-0.440558	-0.180618
C	6.258850	-0.238996	-0.270652
C	6.936980	-1.440244	-0.117918
S	7.420665	1.066547	-0.458612
C	8.346675	-1.314474	-0.150530
H	6.430737	-2.396860	0.004846
C	8.785897	-0.017718	-0.335861
H	9.024607	-2.162758	-0.073177
C	-6.359335	-0.768668	-0.149320
C	-6.930727	-2.020016	0.026238
S	-7.627995	0.434750	-0.334690
C	-8.347027	-2.011629	0.011552
H	-6.344032	-2.926087	0.171380
C	-8.897250	-0.758014	-0.165889
H	-8.957475	-2.904357	0.140628
N	-0.658890	-2.346049	0.117642
N	0.716974	-2.288595	0.102727
C	-4.965424	2.117235	-0.545799
H	-5.594179	2.130970	-1.448860

H	-13.893861	2.565514	0.020460
H	-14.048571	0.344919	1.044083
H	-12.598111	4.301219	-1.185615
C	10.266553	-0.326749	0.228028
C	10.862601	0.925209	-0.165592
C	11.051443	-1.280537	0.865505
C	10.157530	1.935786	-0.880595
C	12.247220	1.160604	0.148550
C	12.415671	-1.047427	1.152821
H	10.594479	-2.221915	1.170940
C	10.769718	3.119896	-1.237827
H	9.119781	1.766881	-1.159602
C	12.844984	2.396427	-0.229976
C	12.999991	0.152649	0.812441
H	12.996078	-1.819064	1.660607
C	12.124895	3.359934	-0.902505
H	10.206416	3.874691	-1.788649
H	13.893868	2.565515	0.019842
H	14.048549	0.345253	1.044221
H	12.598145	4.300822	-1.186827

Ground state. DCM.
2,5-Bis(3-methyl-5'-(naphthalen-1-yl)-2,2'-bithiophen-5-yl)-1,3,4-oxadiazole. **Conformer AA1.**
IEFPCM-B3LYP/Def2-SVPD/IEFPCM(DCM)

Atom	X	Y	Z
C	-1.066919	-0.758045	0.071954
C	1.066945	-0.758048	-0.072927
O	0.000015	0.087022	-0.000479
C	2.395092	-0.217786	-0.160983
C	2.773815	1.108978	-0.190551
S	3.782603	-1.267775	-0.244062
C	4.177041	1.311867	-0.284866
H	2.056682	1.927025	-0.149630
C	4.878359	0.104515	-0.332727
C	-2.395058	-0.217777	0.160088
C	-2.773772	1.108989	0.189660
S	-3.782561	-1.267757	0.243377
C	-4.176987	1.311886	0.284118
H	-2.056640	1.927032	0.148641
C	-4.878304	0.104540	0.332095
C	6.299457	-0.162831	-0.438570
C	6.914733	-1.390381	-0.639648
S	7.525723	1.086438	-0.284706
C	8.328914	-1.328036	-0.667541
H	6.359322	-2.316842	-0.780242
C	8.833785	-0.053452	-0.495686
H	8.962051	-2.195406	-0.846499
C	-6.299384	-0.162808	0.438163
C	-6.914619	-1.390360	0.639349
S	-7.525680	1.086467	0.284601
C	-8.328793	-1.328019	0.667533
H	-6.359178	-2.316820	0.779824
C	-8.833702	-0.053433	0.495794
H	-8.961889	-2.195394	0.846620
N	-0.686978	-2.006718	0.046315
N	0.686998	-2.006720	-0.047324
C	-4.786534	2.686545	0.329257
H	-5.407754	2.887169	-0.556901

H	-5.611361	2.378769	0.305866
H	-4.213117	2.907695	-0.650065
C	4.620831	2.514918	-0.666118
H	5.245429	2.844074	0.178048
H	5.241735	2.569136	-1.573081
H	3.802922	3.236258	-0.777340
C	10.157556	0.503703	-0.484346
C	11.205018	0.153779	0.444302
C	10.453040	1.348216	-1.549347
C	10.985871	-0.638811	1.607252
C	12.534973	0.648130	0.204418
C	11.757584	1.846983	-1.762506
H	9.664444	1.608070	-2.255972
C	12.020196	-0.953736	2.464877
H	9.980843	-0.987732	1.832783
C	13.580400	0.295778	1.104990
C	12.782306	1.493639	-0.912269
H	11.949156	2.498253	-2.616416
C	13.334906	-0.493658	2.207522
H	11.821714	-1.557177	3.351922
H	14.586155	0.670536	0.907413
H	13.795564	1.861090	-1.080949
H	14.146471	-0.753407	2.888753
C	-10.328001	-0.424661	-0.319949
C	-10.985937	0.593523	0.459911
C	-11.064360	-1.143293	-1.254957
C	-10.333804	1.335397	1.486625
C	-12.379502	0.858157	0.216199
C	-12.437747	-0.889954	-1.472508
H	-10.561402	-1.904694	-1.851278
C	-11.004371	2.302040	2.208150
H	-9.290247	1.129198	1.713997
C	-13.038115	1.867173	0.975032
C	-13.080810	0.098786	-0.760968
H	-12.978254	-1.472070	-2.220259
C	-12.368313	2.580092	1.945765
H	-10.480723	2.852126	2.991505
H	-14.093059	2.063199	0.775776
H	-14.136926	0.310646	-0.934311
H	-12.887933	3.348600	2.519795

H	-5.426094	2.821434	1.214134
H	-4.000221	3.449673	0.362779
C	4.786609	2.686518	-0.329968
H	5.407685	2.887178	0.556284
H	5.426316	2.821359	-1.214746
H	4.000308	3.449652	-0.363655
C	10.229399	0.422484	-0.526045
C	11.265330	-0.223350	0.243628
C	10.558974	1.517474	-1.317906
C	11.015830	-1.299720	1.142318
C	12.616772	0.255991	0.122327
C	11.885512	1.994393	-1.411823
H	9.778750	2.002335	-1.905129
C	12.039726	-1.890802	1.854214
H	9.996156	-1.650349	1.283351
C	13.650226	-0.383760	0.864470
C	12.897719	1.368877	-0.717604
H	12.103066	2.849871	-2.052783
C	13.373994	-1.438831	1.706820
H	11.817866	-2.709116	2.540853
H	14.672131	-0.015969	0.757160
H	13.926960	1.721488	-0.798211
H	14.176988	-1.917149	2.269484
C	-10.229304	0.422515	0.526477
C	-11.265432	-0.223326	-0.242924
C	-10.558676	1.517521	1.318403
C	-11.016163	-1.299714	-1.141659
C	-12.616843	0.256015	-0.121282
C	-11.885188	1.994446	1.412647
H	-9.778303	2.002389	1.905424
C	-12.040244	-1.890811	-1.853278
H	-9.996524	-1.650340	-1.282950
C	-13.650489	-0.383754	-0.863142
C	-12.897574	1.368917	0.718698
H	-12.102578	2.849936	2.053647
C	-13.374474	-1.438842	-1.705543
H	-11.818564	-2.709138	-2.539961
H	-14.672366	-0.015965	-0.755572
H	-13.926795	1.721528	0.799561
H	-14.177615	-1.917174	-2.267985

Ground state. DCM.
2,5-Bis(3-methyl-5'-(naphthalen-1-yl)-2,2'-bithiophen-5-yl)-1,3,4-oxadiazole. **Conformer AA2.**
IEFPCM-B3LYP/Def2-SVPD/IEFPCM(DCM)

Atom	X	Y	Z
C	1.069393	0.439963	-0.800825
C	-1.069390	0.439964	-0.800822
O	0.000002	-0.326284	-0.444292
C	-2.400508	-0.046042	-0.564822
C	-2.780423	-1.255841	-0.019981
S	-3.790122	0.924949	-0.965566
C	-4.186740	-1.432348	0.082063
H	-2.062395	-2.009987	0.297593
C	-4.888993	-0.326927	-0.403595
C	2.400511	-0.046045	-0.564829
C	2.780427	-1.255844	-0.019988
S	3.790125	0.924946	-0.965577
C	4.186744	-1.432352	0.082051

H	2.062400	-2.009989	0.297589
C	4.888996	-0.326931	-0.403611
C	-6.314033	-0.076865	-0.500549
C	-6.945730	0.951383	-1.185259
S	-7.517587	-1.058483	0.320969
C	-8.355415	0.950973	-1.051089
H	-6.406369	1.682233	-1.786522
C	-8.839978	-0.078702	-0.267714
H	-9.003937	1.669575	-1.549367
C	6.314037	-0.076870	-0.500567
C	6.945732	0.951381	-1.185273
S	7.517592	-1.058500	0.320933
C	8.355416	0.950974	-1.051102
H	6.406370	1.682236	-1.786529
C	8.839981	-0.078704	-0.267729
H	9.003937	1.669581	-1.549372
N	0.688583	1.571337	-1.329041
N	-0.688580	1.571337	-1.329039
C	4.798553	-2.684792	0.648508
H	5.538812	-3.124148	-0.036190
H	5.310009	-2.493488	1.604322
H	4.022040	-3.436285	0.833736
C	-4.798548	-2.684788	0.648523
H	-5.309993	-2.493485	1.604343
H	-5.538815	-3.124139	-0.036168
H	-4.022036	-3.436284	0.833741
C	-10.231721	-0.446661	0.053044
C	-11.177424	0.528983	0.538886
C	-10.646303	-1.763162	-0.118700
C	-10.830629	1.881483	0.820440
C	-12.534985	0.115977	0.778516
C	-11.976644	-2.162612	0.139999
H	-9.934445	-2.500067	-0.491391
C	-11.770517	2.781390	1.280125
H	-9.801383	2.206819	0.688125
C	-13.480760	1.073139	1.244675
C	-12.906984	-1.240361	0.566440
H	-12.264083	-3.203004	-0.018011
C	-13.113359	2.379577	1.484359
H	-11.474366	3.809436	1.494077
H	-14.508515	0.748354	1.415809
H	-13.939857	-1.538699	0.751874
H	-13.849648	3.100270	1.843025
C	10.231724	-0.446657	0.053034
C	11.177419	0.528987	0.538894
C	10.646315	-1.763154	-0.118722
C	10.830612	1.881480	0.820468
C	12.534980	0.115986	0.778528
C	11.976656	-2.162599	0.139983
H	9.934464	-2.500059	-0.491427
C	11.770492	2.781387	1.280168
H	9.801364	2.206811	0.688154
C	13.480747	1.073149	1.244703
C	12.906989	-1.240348	0.566439
H	12.264102	-3.202988	-0.018036
C	13.113336	2.379581	1.484404
H	11.474333	3.809428	1.494133
H	14.508503	0.748368	1.415839
H	13.939862	-1.538682	0.751875

H	13.849618	3.100274	1.843083

d) Cartesian coordinates for **15f** obtained with B3LYP functional for ground states.

Ground state. DCM.
2,5-Bis(5'-(anthracen-9-yl)-3-methyl-2,2'-bithiophen-5-yl)-1,3,4-oxadiazole. **Conformer A**
IEFPCM-B3LYP/Def2-SVPD/IEFPCM(DCM)

Atom	X	Y	Z
C	-1.067128	0.068846	-0.999228
C	1.066992	-0.063613	-0.999379
O	-0.000053	0.001967	-0.154524
C	2.397950	-0.141486	-0.462274
C	2.783266	-0.158626	0.863189
S	3.780709	-0.224634	-1.517790
C	4.188703	-0.249057	1.059046
H	2.070046	-0.118130	1.684819
C	4.879851	-0.308059	-0.150982
C	-2.398066	0.145788	-0.461957
C	-2.783315	0.161281	0.863548
S	-3.780894	0.229928	-1.517306
C	-4.188783	0.250920	1.059590
H	-2.070037	0.120042	1.685089
C	-4.880033	0.310989	-0.150350
C	6.301978	-0.422657	-0.426189
C	6.901523	-0.893140	-1.582232
S	7.536254	0.122514	0.699620
C	8.320160	-0.818721	-1.563268
H	6.334363	-1.296497	-2.420167
C	8.830591	-0.292578	-0.398611
H	8.955368	-1.152454	-2.382701
C	-6.302253	0.425056	-0.425376
C	-6.902126	0.896752	-1.580765
S	-7.536172	-0.122396	0.699719
C	-8.320705	0.821143	-1.561992
H	-6.335240	1.301663	-2.418136
C	-8.830765	0.292888	-0.398134
H	-8.956135	1.155438	-2.381019
N	-0.687425	0.046270	-2.247787
N	0.687232	-0.039052	-2.247886
C	-4.811566	0.287419	2.429375
H	-5.577086	1.071905	2.508651
H	-5.295476	-0.668782	2.681390
H	-4.045804	0.479565	3.190597
C	4.811544	-0.287547	2.428747
H	5.576675	-1.072514	2.507022
H	5.295950	0.668100	2.681903
H	4.045728	-0.480240	3.189772
C	-10.837628	-3.379557	-1.371270
C	-10.198652	-2.206273	-1.055747
C	-10.880538	-1.152604	-0.362923
C	-12.267141	-1.362420	-0.005628
C	-12.895986	-2.601236	-0.355850
C	-12.204982	-3.583214	-1.018472
C	-10.249662	0.070631	-0.023767
C	-12.962715	-0.354111	0.674063
C	-12.351135	0.858434	1.018291
C	-10.965561	1.081652	0.664960
C	-10.372671	2.335124	1.029230

Ground state. DCM.
2,5-Bis(5'-(anthracen-9-yl)-3-methyl-2,2'-bithiophen-5-yl)-1,3,4-oxadiazole. **Conformer S**
IEFPCM-B3LYP/Def2-SVPD/IEFPCM(DCM)

Atom	X	Y	Z
C	-1.068924	0.022713	1.108477
C	1.068586	-0.044404	1.107918
O	-0.000168	-0.003773	0.263044
C	2.400208	-0.077459	0.569594
C	2.784783	-0.091135	-0.755404
S	3.785930	-0.099140	1.624995
C	4.192265	-0.122688	-0.952413
H	2.070110	-0.081813	-1.576576
C	4.889851	-0.134723	0.257226
C	-2.400514	0.065464	0.570718
C	-2.784921	0.101118	-0.753936
S	-3.786315	0.071574	1.626214
C	-4.192339	0.137868	-0.950500
H	-2.070079	0.104352	-1.575008
C	-4.889961	0.131497	0.259138
C	6.314973	-0.164364	0.531079
C	6.932647	-0.290560	1.765475
S	7.539110	-0.000209	-0.721944
C	8.350390	-0.253346	1.706483
H	6.379962	-0.409937	2.696428
C	8.846500	-0.098956	0.432026
H	8.995523	-0.338998	2.579787
C	-6.315083	0.159887	0.533286
C	-6.932693	0.276102	1.768668
S	-7.539196	0.006079	-0.721039
C	-8.350467	0.239702	1.709325
H	-6.379898	0.388262	2.700456
C	-8.846602	0.095863	0.433655
H	-8.995600	0.318821	2.583229
N	-0.688380	0.000376	2.356891
N	0.688028	-0.043157	2.356526
C	-4.809141	0.179630	-2.322071
H	-5.397379	-0.726643	-2.532364
H	-5.483098	1.040948	-2.441336
H	-4.028247	0.254244	-3.087756
C	4.808898	-0.140980	-2.324538
H	5.399474	0.767412	-2.518402
H	5.480530	-1.001777	-2.459695
H	4.027659	-0.199685	-3.091250
C	-11.095066	3.633135	-0.537606
C	-10.379316	2.502951	-0.229160
C	-10.971430	1.200671	-0.322790
C	-12.351102	1.111945	-0.751504
C	-13.061868	2.316791	-1.061097
C	-12.455127	3.542577	-0.959393
C	-10.260075	0.014810	-0.011724
C	-12.960327	-0.145311	-0.855765
C	-12.268980	-1.325596	-0.553180
C	-10.889664	-1.250561	-0.121335
C	-10.214061	-2.477622	0.184081

H	-9.333398	2.521818	0.764866	H	-9.178397	-2.439248	0.516839
C	-11.092489	3.294357	1.697404	C	-10.850957	-3.688137	0.067331
C	-12.457774	3.070683	2.046081	C	-12.209940	-3.759027	-0.361294
C	-13.066023	1.887454	1.713052	C	-12.895621	-2.609533	-0.660411
H	-10.296485	-4.166580	-1.898620	H	-10.621734	4.612997	-0.458936
H	-9.156132	-2.063613	-1.334292	H	-9.343603	2.586932	0.095193
H	-13.942115	-2.747094	-0.081627	H	-14.101907	2.237435	-1.381969
H	-12.696341	-4.521761	-1.278306	H	-13.008508	4.451469	-1.199268
H	-14.007800	-0.517310	0.942501	H	-14.000069	-0.206585	-1.181376
H	-10.618728	4.240296	1.964031	H	-10.314845	-4.607879	0.306069
H	-13.014298	3.844891	2.575907	H	-12.699779	-4.729791	-0.447790
H	-14.110506	1.707981	1.973306	H	-13.935918	-2.652646	-0.987112
C	11.088849	-3.298752	1.693729	C	10.844975	3.690394	0.088953
C	10.370151	-2.337993	1.026582	C	10.209912	2.478203	0.198091
C	10.964402	-1.084724	0.663880	C	10.887618	1.254030	-0.114211
C	12.350130	-0.863349	1.017730	C	12.267132	1.333782	-0.544621
C	13.063816	-1.893917	1.711405	C	12.891813	2.619326	-0.643847
C	12.454280	-3.076891	2.042939	C	12.204121	3.765957	-0.338377
C	10.249650	-0.072155	-0.023756	C	10.260016	-0.013026	-0.012352
C	12.963045	0.348949	0.675019	C	12.960641	0.156397	-0.853431
C	12.268664	1.358786	-0.003620	C	12.353476	-1.102445	-0.756837
C	10.881874	1.150866	-0.361371	C	10.973578	-1.195926	-0.329781
C	10.201230	2.206127	-1.053079	C	10.383558	-2.499790	-0.244288
H	9.158592	2.064931	-1.331941	H	9.347663	-2.587442	0.078554
C	10.841551	3.379100	-1.367126	C	11.101505	-3.626949	-0.558695
C	12.209073	3.580842	-1.013876	C	12.461795	-3.531612	-0.978637
C	12.898901	2.597339	-0.352293	C	13.066505	-2.304250	-1.072742
H	10.614061	-4.244512	1.959154	H	10.307316	4.607867	0.332901
H	9.330739	-2.523301	0.761810	H	9.174126	2.436218	0.530053
H	14.108436	-1.715837	1.972066	H	13.932255	2.666014	-0.969603
H	13.009898	-3.852290	2.571959	H	12.692505	4.737983	-0.418747
H	14.008252	0.510721	0.943840	H	14.000508	0.221251	-1.177905
H	10.301352	4.167338	-1.893633	H	10.629780	-4.608063	-0.486252
H	12.701498	4.519158	-1.272531	H	13.016939	-4.438137	-1.223276
H	13.945142	2.741743	-0.077734	H	14.106647	-2.221279	-1.392278

References

1. Williams, A. T. R.; Winfield, S. A.; Miller, J. N. *Analyst*, **1983**, *108*, 1067-1071.
2. Allen, M. W.; Measurement of Fluorescence Quantum Yields, Thermo Fisher Scientific technical note 52019, Madison, WI, USA.
3. Brouwer, A. M. *Pure and Applied Chemistry*, **2011**, *83*, 2213-2228.
4. Becke, A.D. *J.Chem.Phys*. **1993**, *98*, 5648-5652.
5. Lee, C.; Yang, W.; Parr, R.G. *Phys. Rev. B*, **1988**, *37*, 785-789.
6. Yanai, T.; Tew, D.; Handy, N. *Chem. Phys. Lett*. **2004**, *393*, 51-57.
7. Weigend, F.; Ahlrichs, R. *Phys. Chem. Chem. Phys*. 2005, 7, 3297-3305.
8. Weigend, F. *Phys. Chem. Chem. Phys*. **2006**, *8*, 1057-1065.
9. Cances, E.; Mennucci, B.; Tomasi, J.` *J. Chem. Phys.* **1997**, *107*, 3032−3041.

10. Scalmani, G.; Frisch, M. J.; Mennucci, B.; Tomasi, J.; Cammi, R.; Barone, V. *J. Chem. Phys.* **2006**, 124, 094107.
11. Improta, R.; Barone, V.; Scalmani, G.; Frisch, M. *J. Chem. Ph*ys. **2006**, 125, 054103.
12. Gaussian 09, Revision E.01, Frisch, M. J.; Trucks, G. W.; Schlegel, H. B.; Scuseria, G. E.; Robb, M. A.; Cheeseman, J. R.; Scalmani, G.; Barone, V.; Mennucci, B.; Petersson, G. A.; Nakatsuji, H.; Caricato, M.; Li, X.; Hratchian, H. P.; Izmaylov, A. F.; Bloino, J.; Zheng, G.; Sonnenberg, J. L.; Hada, M.; Ehara, M.; Toyota, K.; Fukuda, R.; Hasegawa, J.; Ishida, M.; Nakajima, T.; Honda, Y.; Kitao, O.; Nakai, H.; Vreven, T.; Montgomery, J. A., Jr.; Peralta, J. E.; Ogliaro, F.; Bearpark, M.; Heyd, J. J.; Brothers, E.; Kudin, K. N.; Staroverov, V. N.; Kobayashi, R.; Normand, J.; Raghavachari, K.; Rendell, A.; Burant, J. C.; Iyengar, S. S.; Tomasi, J.; Cossi, M.; Rega, N.; Millam, J. M.; Klene, M.; Knox, J. E.; Cross, J. B.; Bakken, V.; Adamo, C.; Jaramillo, J.; Gomperts, R.; Stratmann, R. E.; Yazyev, O.; Austin, A. J.; Cammi, R.; Pomelli, C.; Ochterski, J. W.; Martin, R. L.; Morokuma, K.; Zakrzewski, V. G.; Voth, G. A.; Salvador, P.; Dannenberg, J. J.; Dapprich, S.; Daniels, A. D.; Farkas, Ö.; Foresman, J. B.; Ortiz, J. V.; Cioslowski, J.; Fox, D. J. Gaussian 09, revision D.01; Gaussian, Inc., Wallingford CT, 2009.
13. A.S. Kostyuchenko, A.M. Averkov, A.S. Fisyuk. A Simple and Efficient Synthesis of Substituted 2,2’-Bithiophene and 2,2’:5’,2’’-Terthiophene . Org. Lett., 2014, 16, 1833−1835.
14. A.S.Kostyuchenko, V.L.Yurpalov, A. Kurowska, W.Domagala, A. Pron, A.S. Fisyuk. Synthesis of new, highly luminescent bis(2,2’-bithiophen-5-yl) substituted 1,3,4-oxadiazole,1,3,4-thiadiazole and 1,2,4-triazole. Beilstein J. Org. Chem., 2014, 10, 1596–1602.

NMR spectra

Ethyl 3-decyl-5'-phenyl-2,2'-bithiophene-5-carboxylate (7d)

^{1}H NMR (400 MHz, $CDCl_3$)

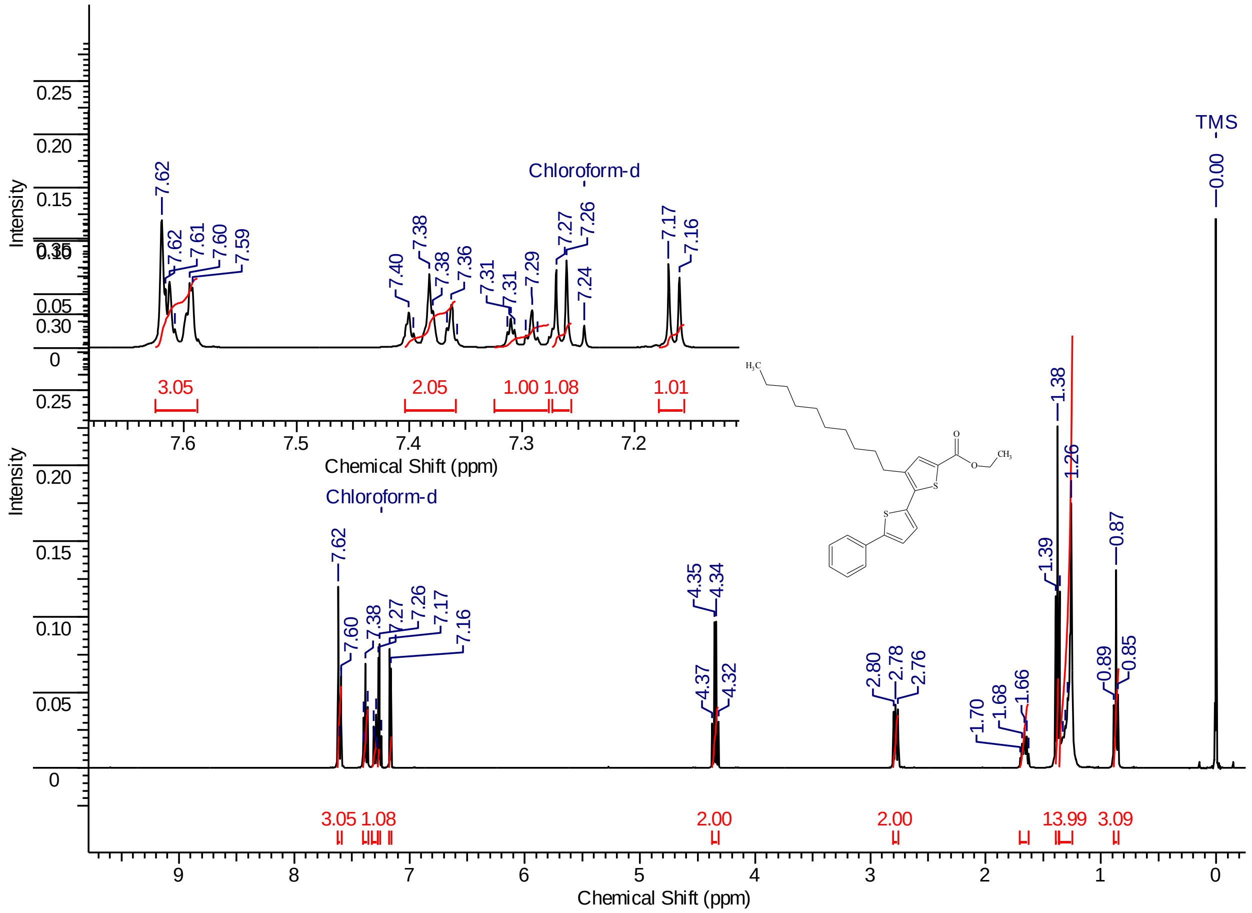

^{13}C NMR (100 MHz) **(7d)**

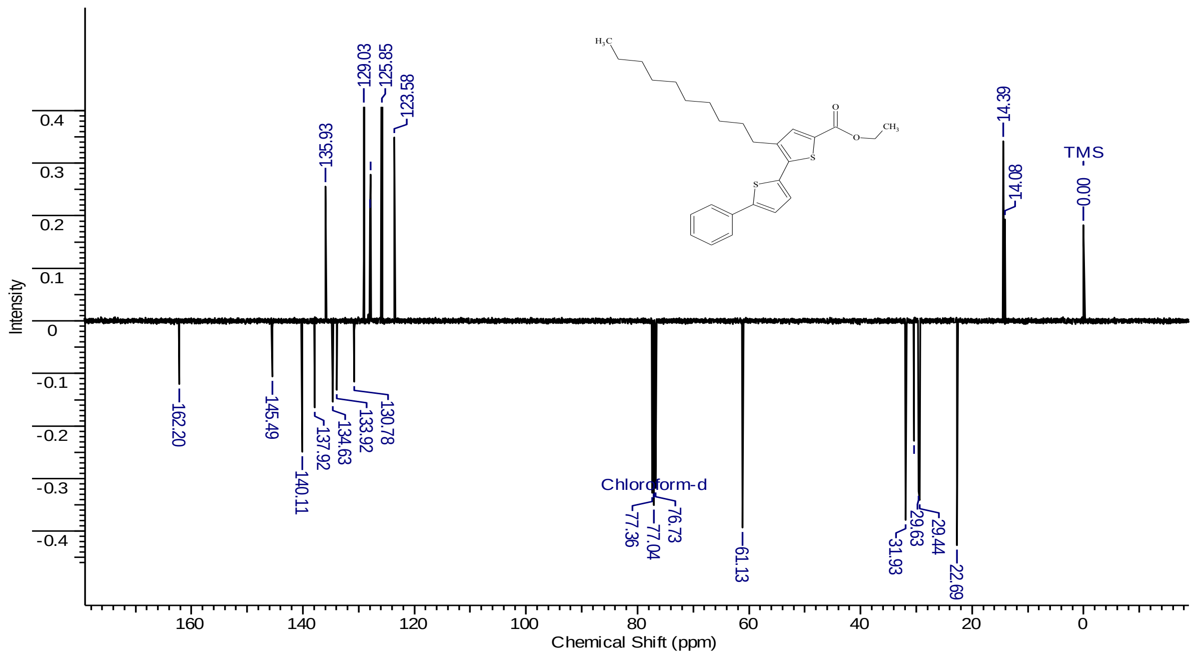

Ethyl 3-decyl-5'-(naphthalen-1-yl)-2,2'-bithiophene-5-carboxylate (7e)

^{1}H NMR (400 MHz, $CDCl_3$)

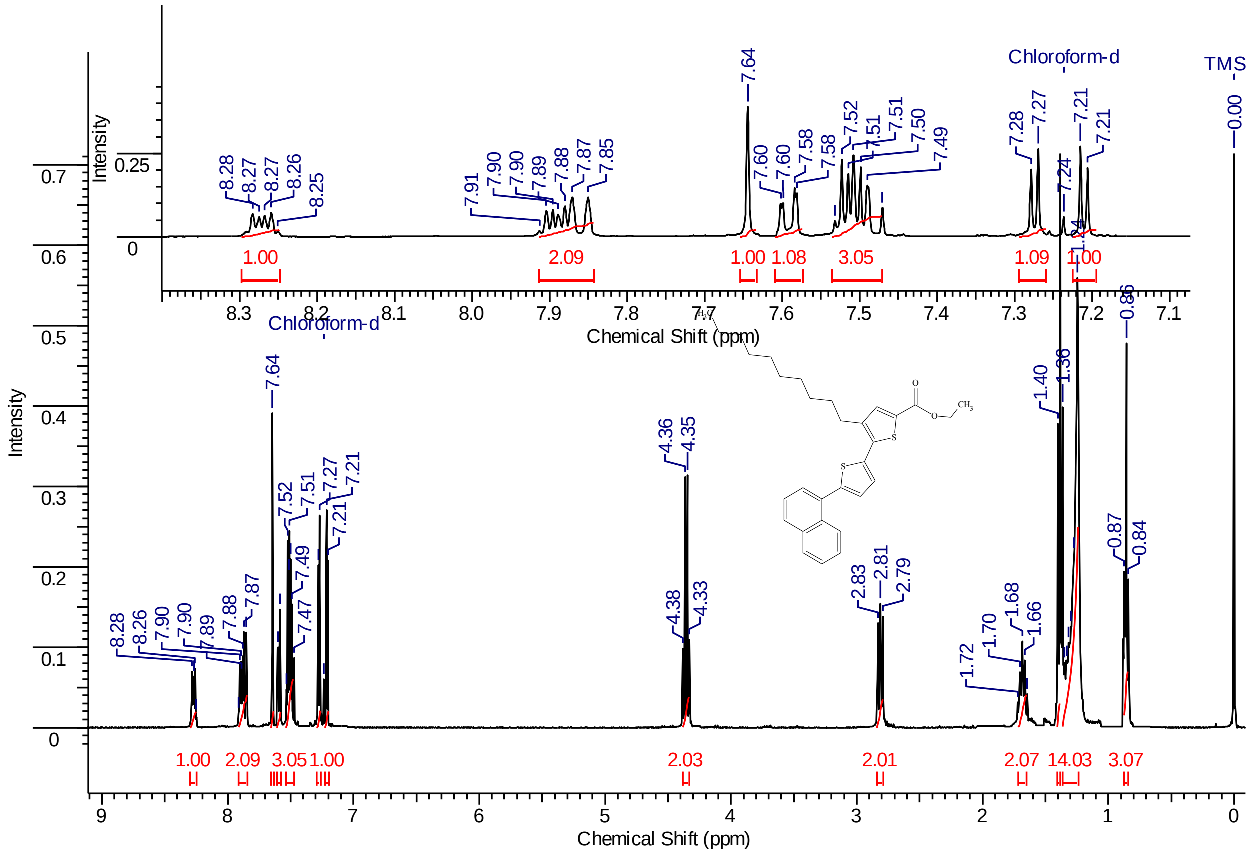

^{13}C NMR (100 MHz)

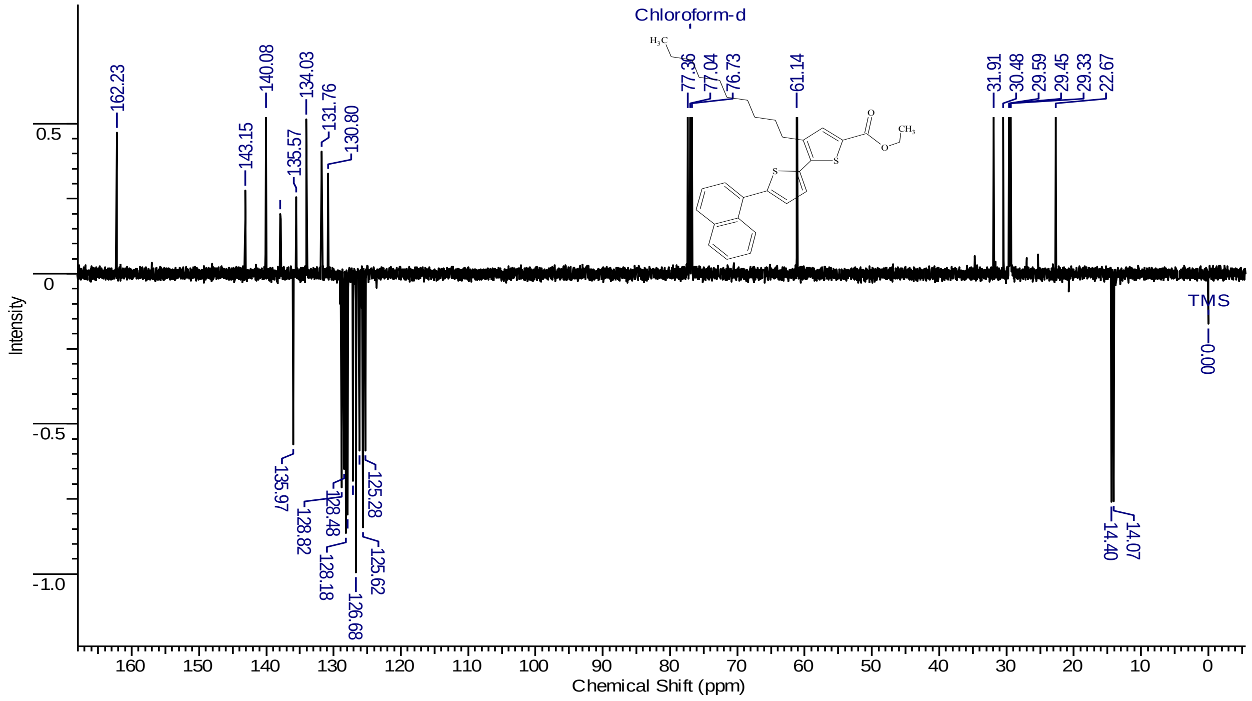

Ethyl 5'-(anthracen-9-yl)-3-decyl-2,2'-bithiophene-5-carboxylate (7f)

^{1}H NMR (400 MHz, $CDCl_3$)

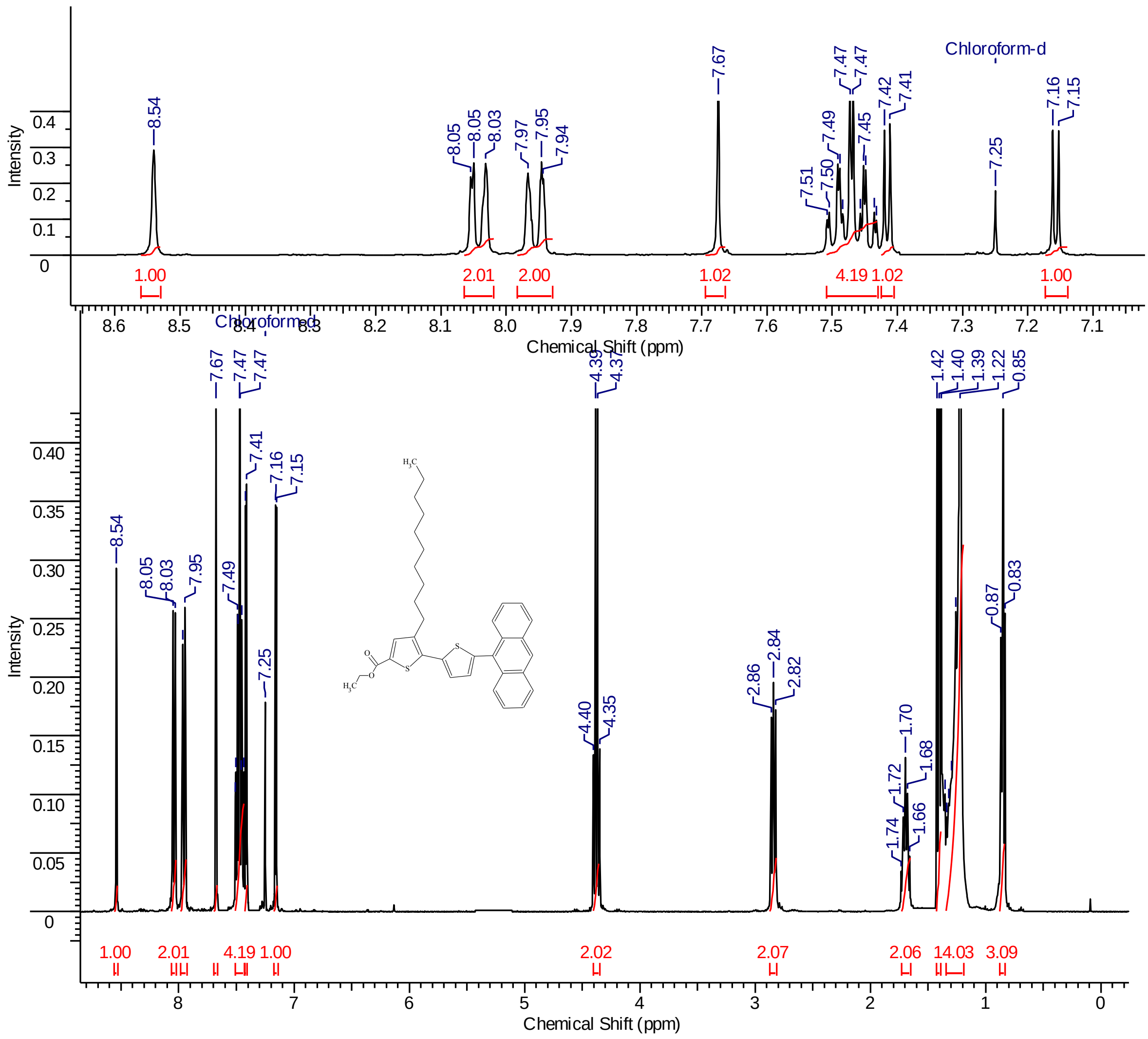

^{13}C NMR (100 MHz) **(7f)**

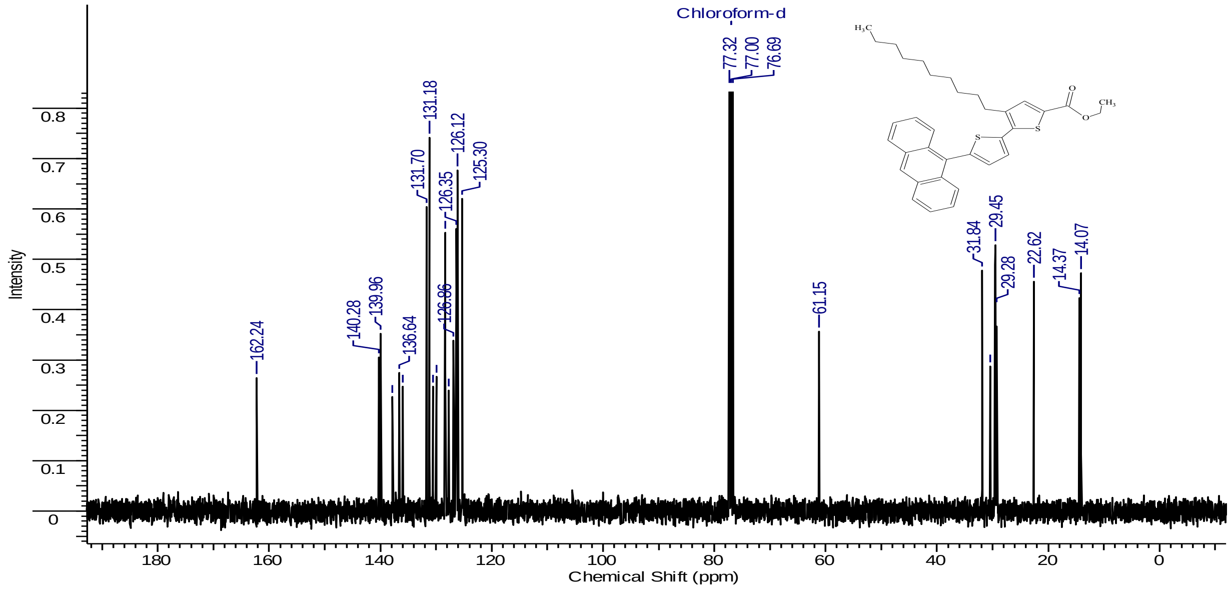

Ethyl 3-decyl-5'-(pyren-1-yl)-2,2'-bithiophene-5-carboxylate (7g)

^{1}**H NMR** (400 MHz, $CDCl_3$)

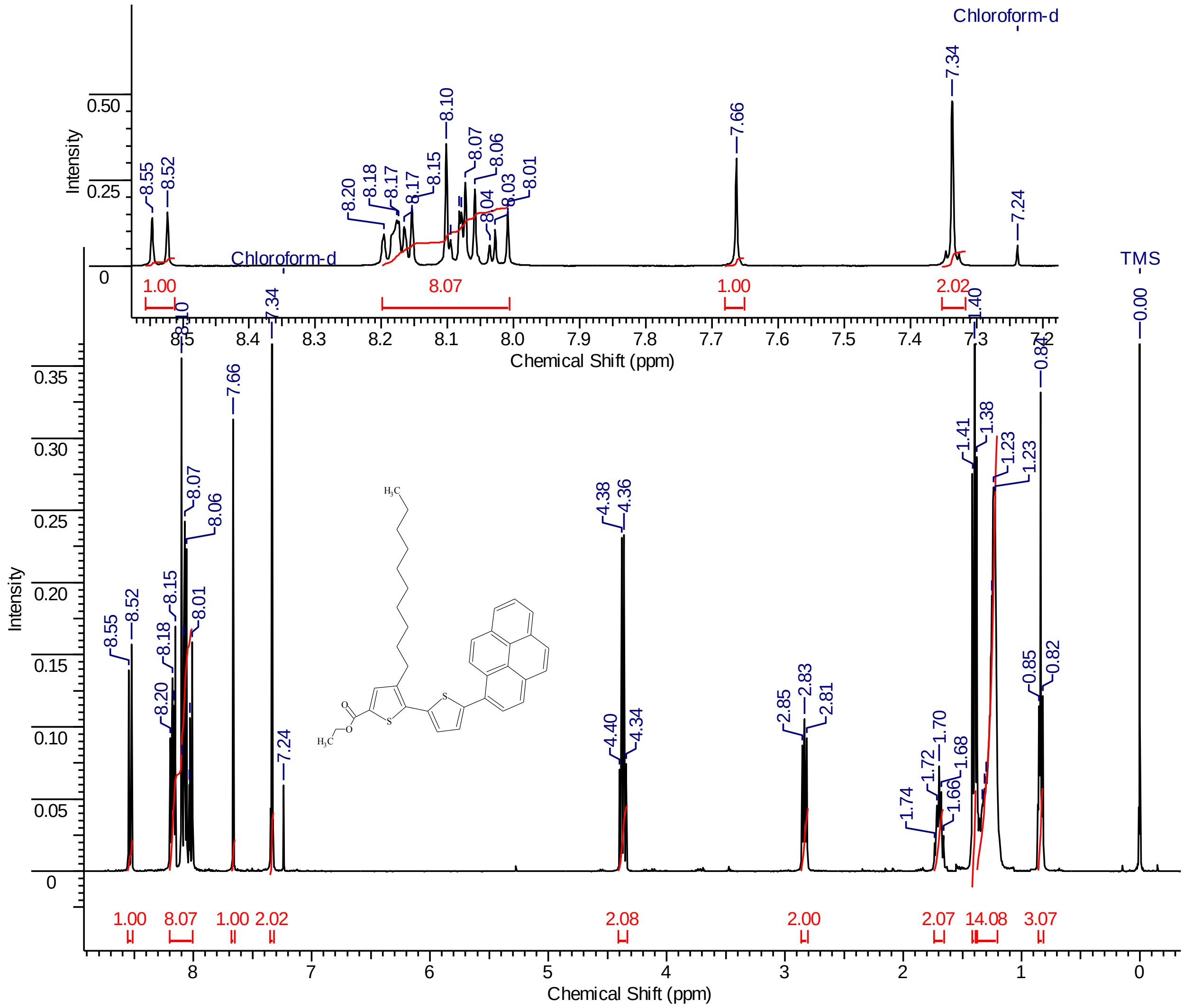

^{13}**C NMR** (100 MHz) **(7g)**

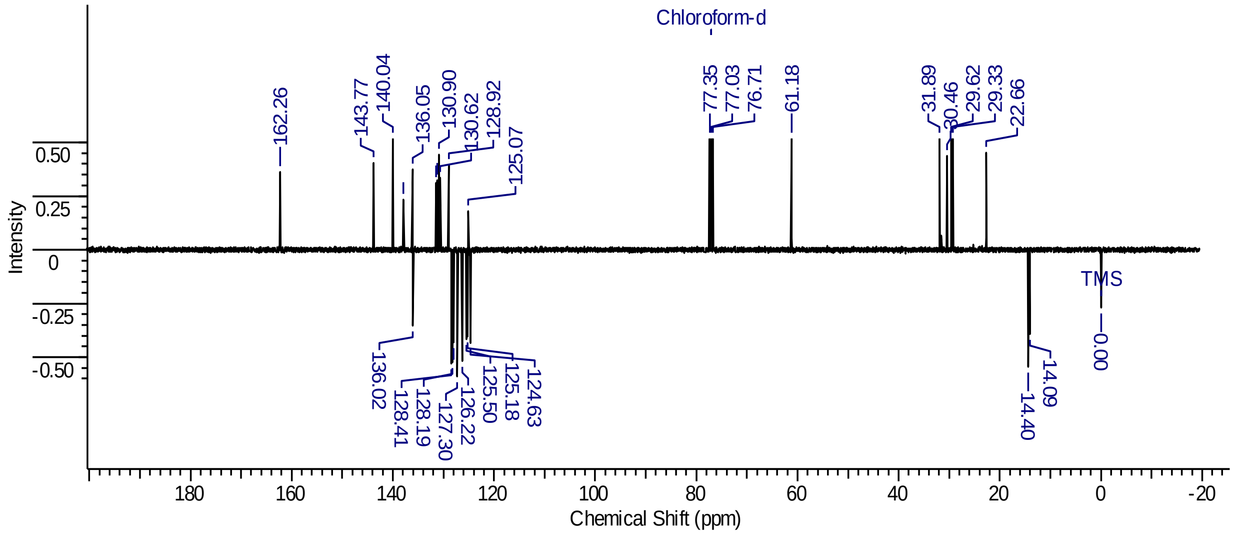

3-Decyl-5'-phenyl-2,2'-bithiophene-5-carboxylic acid (12d)

^{1}H NMR (400 MHz, $CDCl_3$)

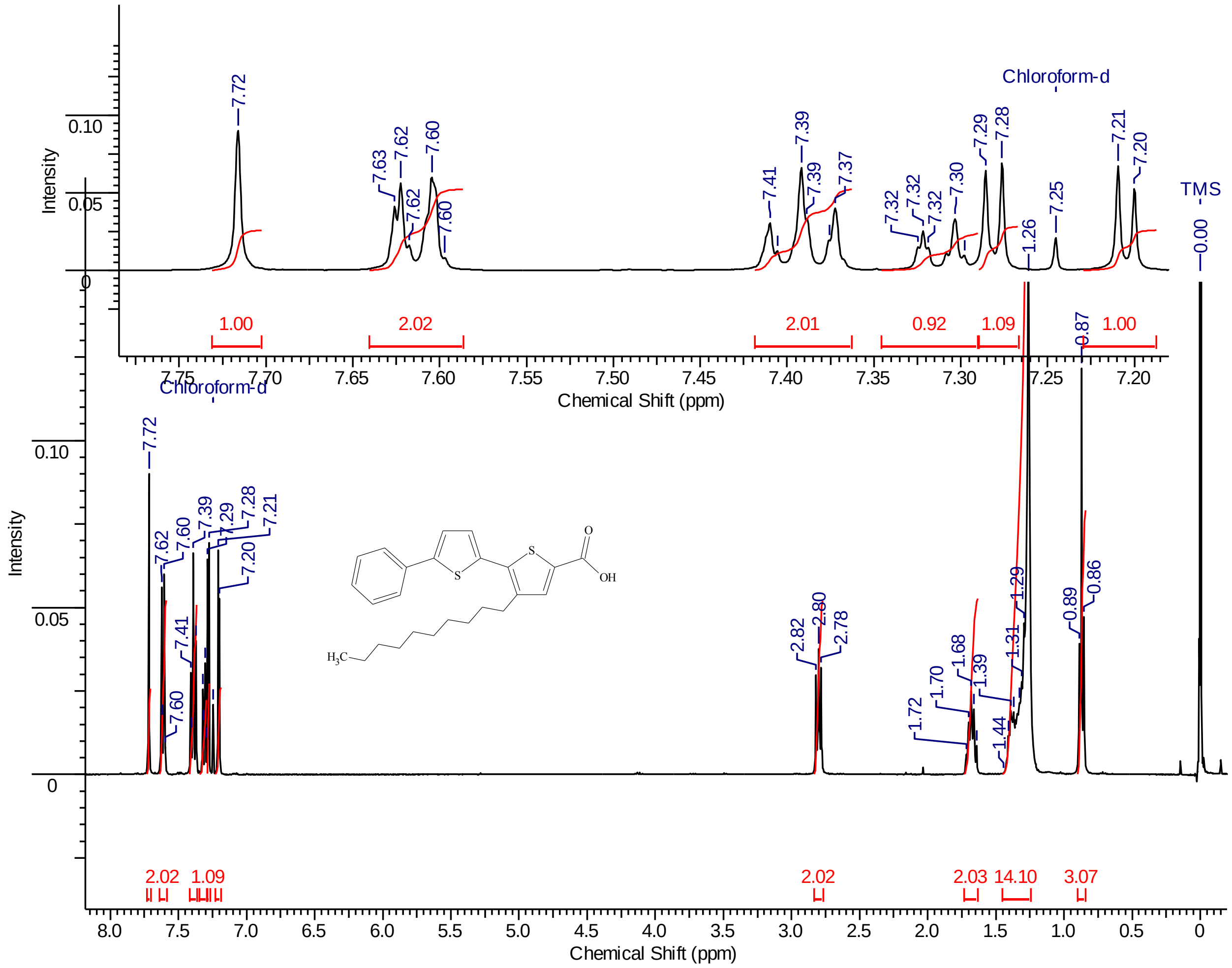

^{13}C NMR (100 MHz) **(12d)**

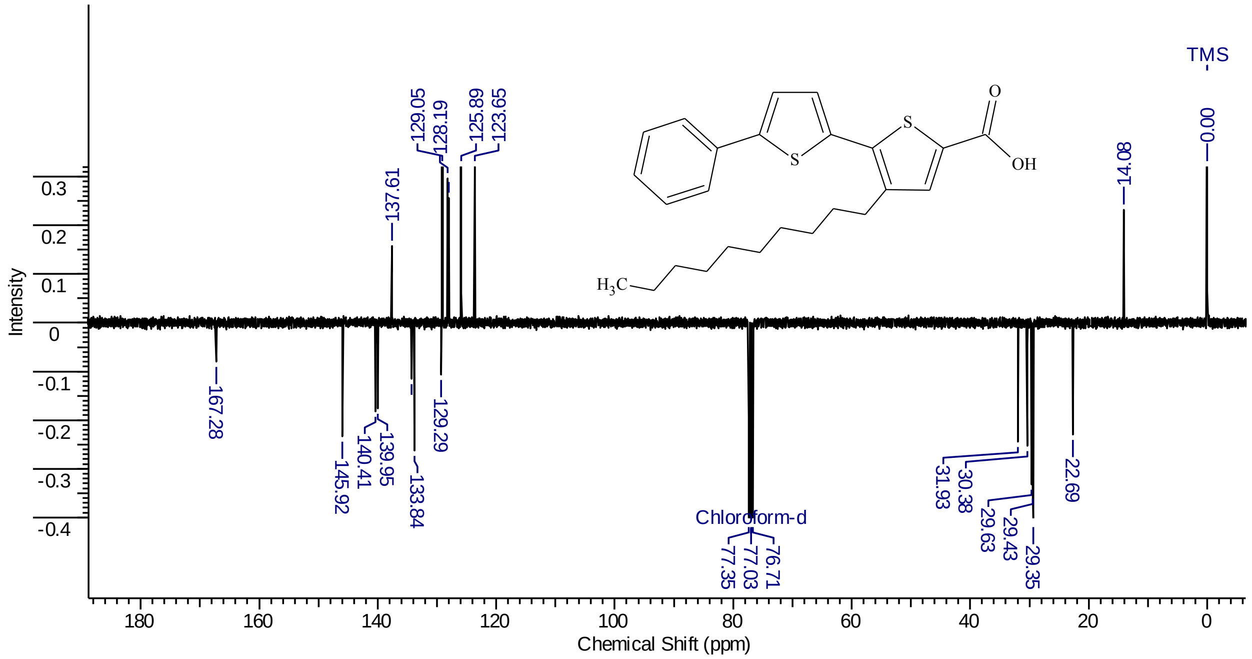

3-Decyl-5'-(naphthalen-1-yl)-2,2'-bithiophene-5-carboxylic acid (12e)

^{1}H NMR (400 MHz, $CDCl_3$)

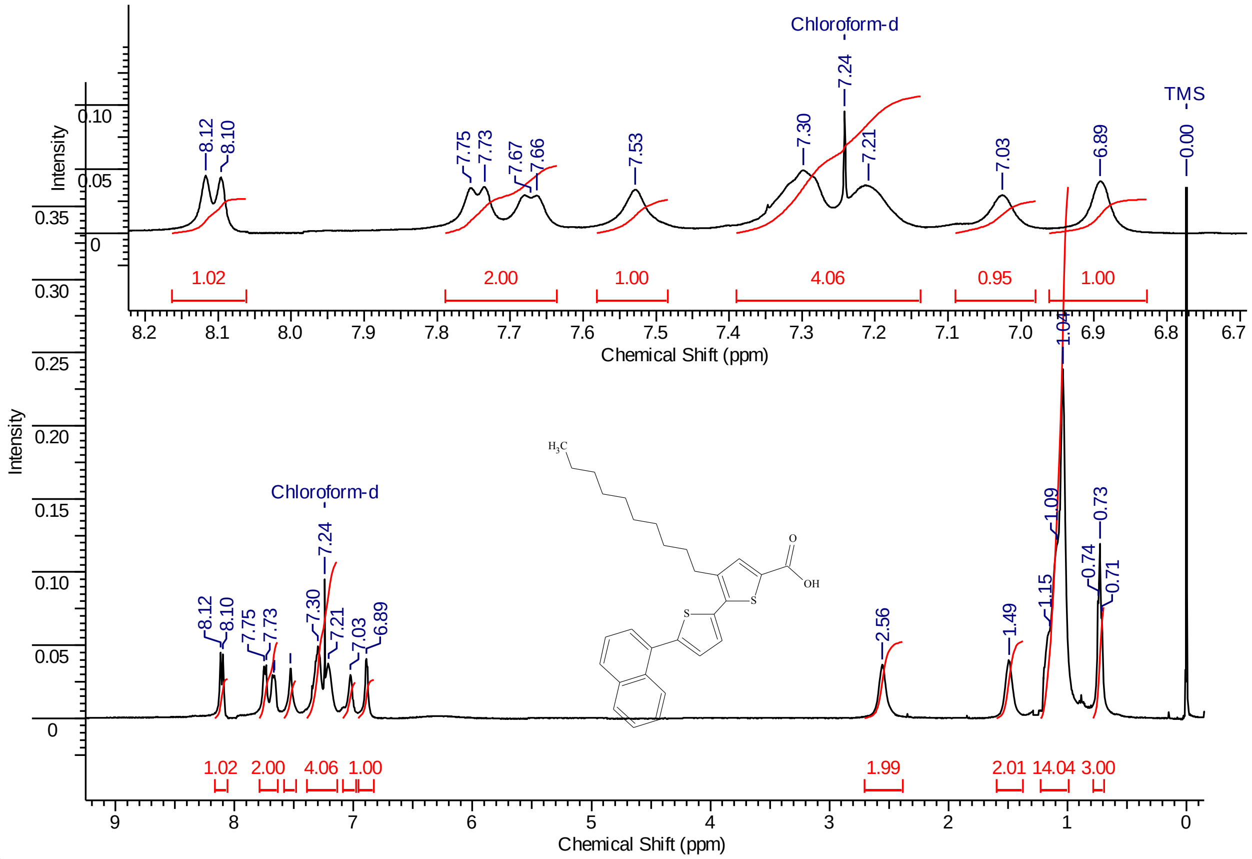

^{13}C NMR (100 MHz)

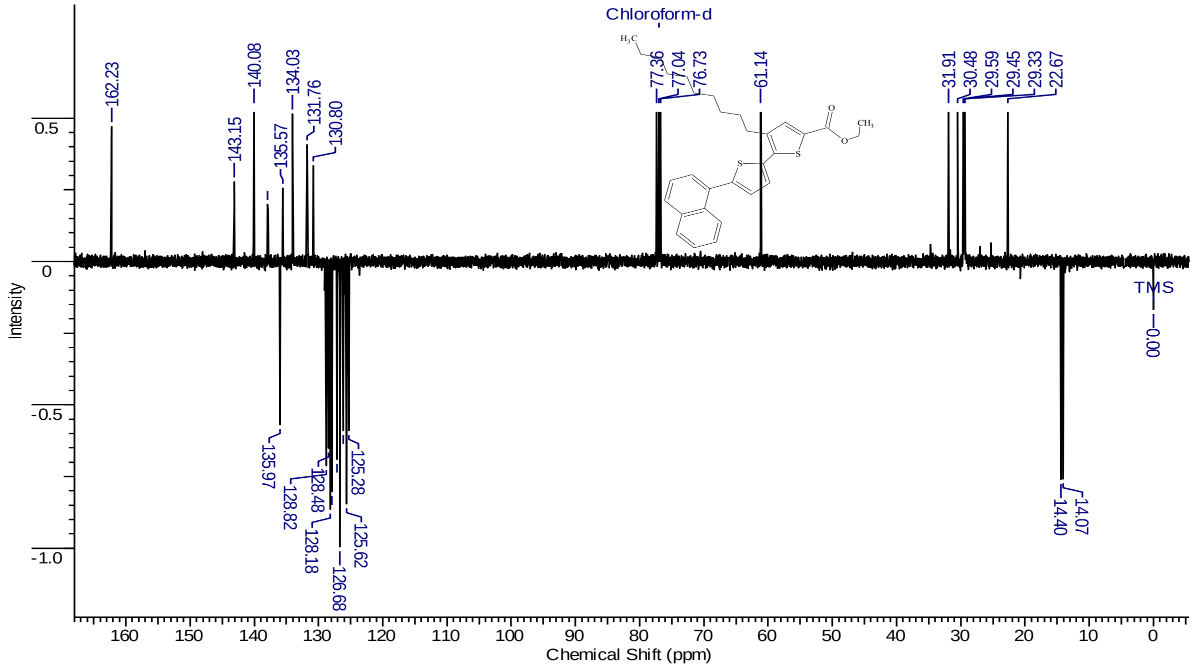

5'-(Anthracen-9-yl)-3-decyl-2,2'-bithiophene-5-carboxylic acid (12f)

^{1}H NMR (400 MHz, $CDCl_3$)

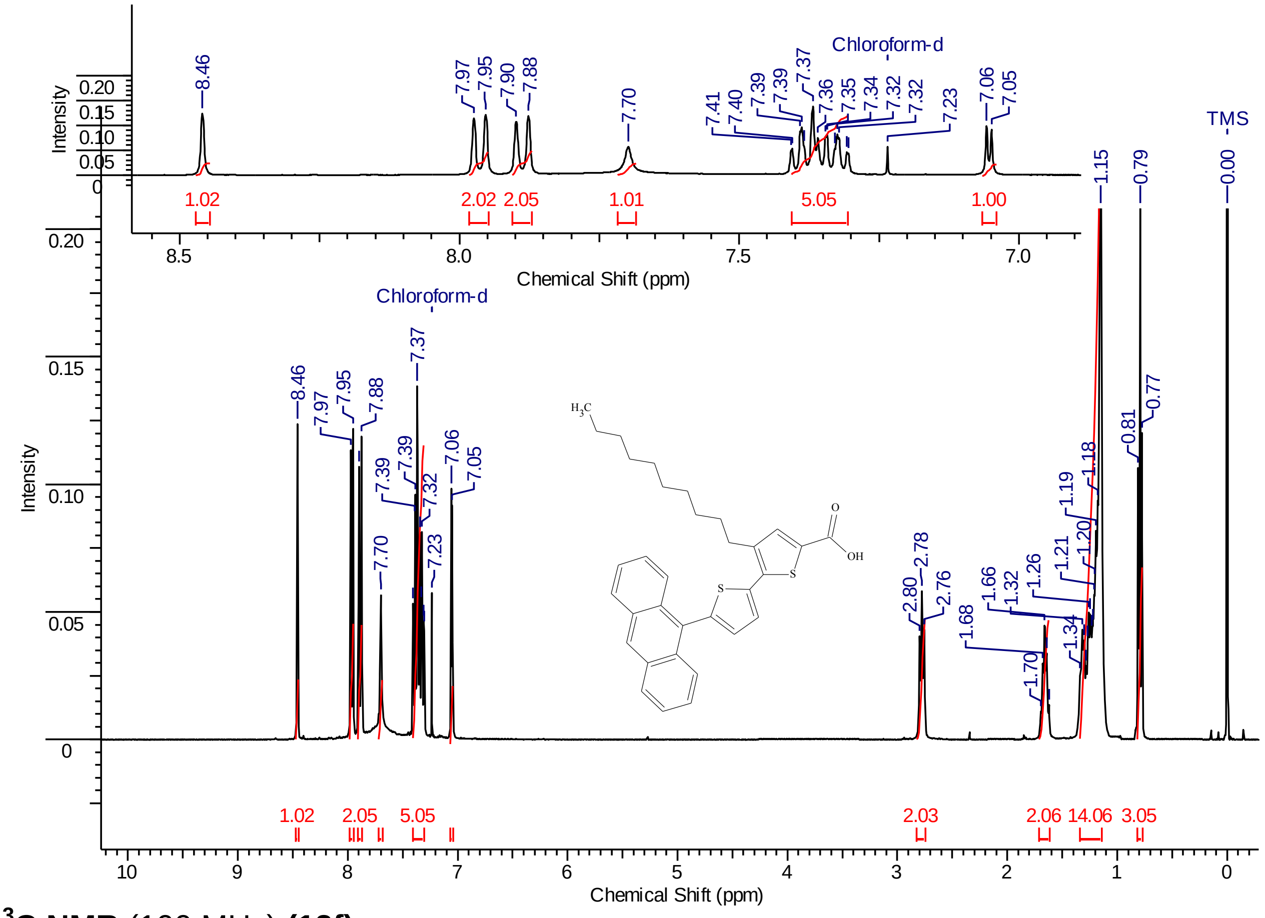

^{13}C NMR (100 MHz) **(12f)**

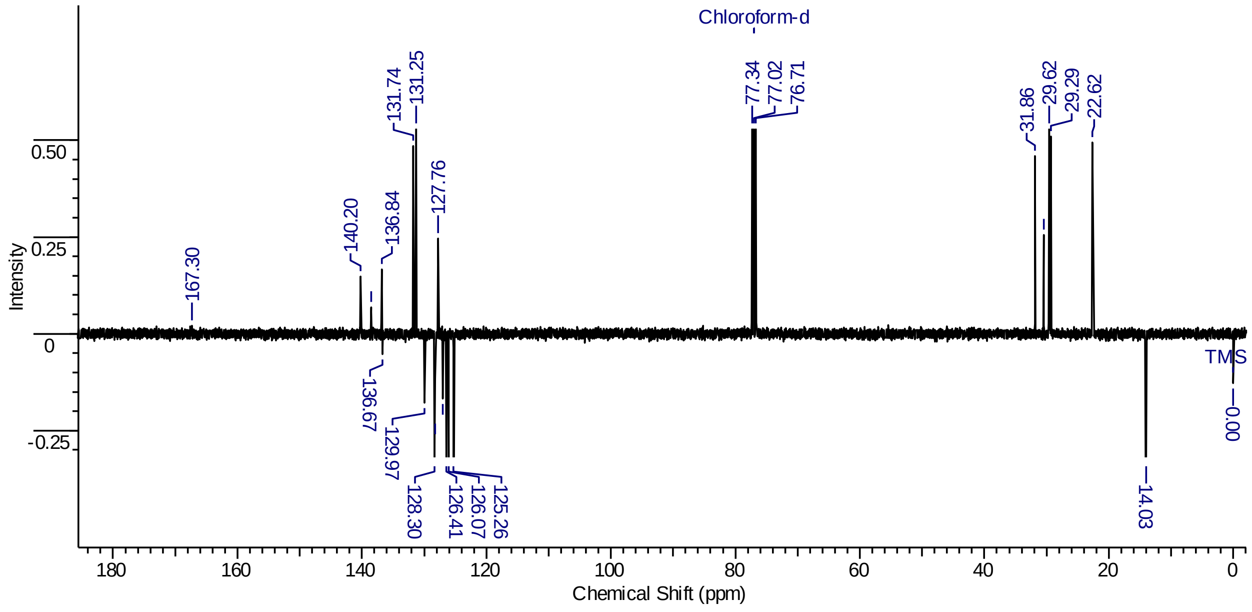

3-Decyl-5'-(pyren-1-yl)-2,2'-bithiophene-5-carboxylic acid (12g)

^{1}H NMR (400 MHz, $CDCl_3$)

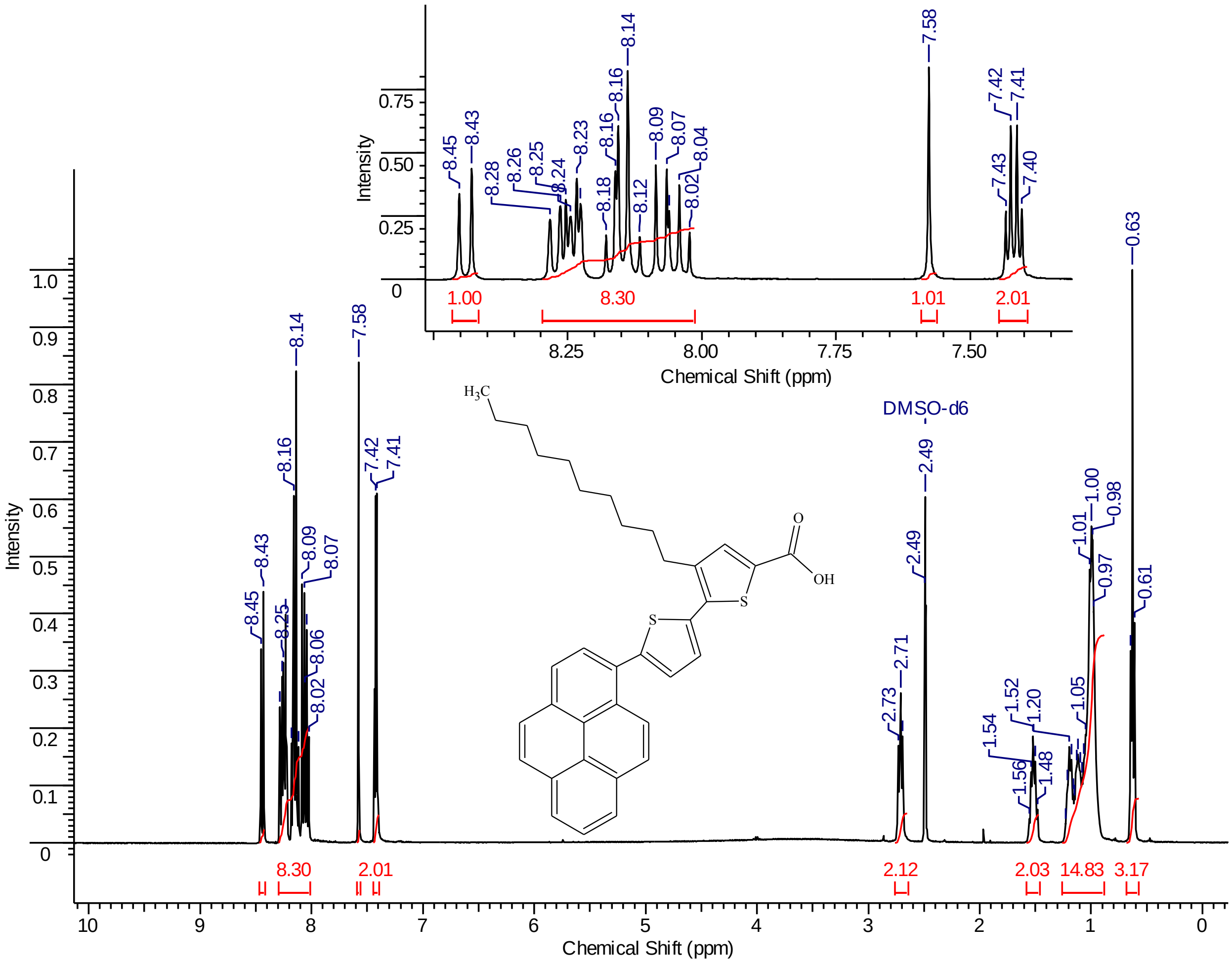

^{13}C NMR (100 MHz) **(12g)**

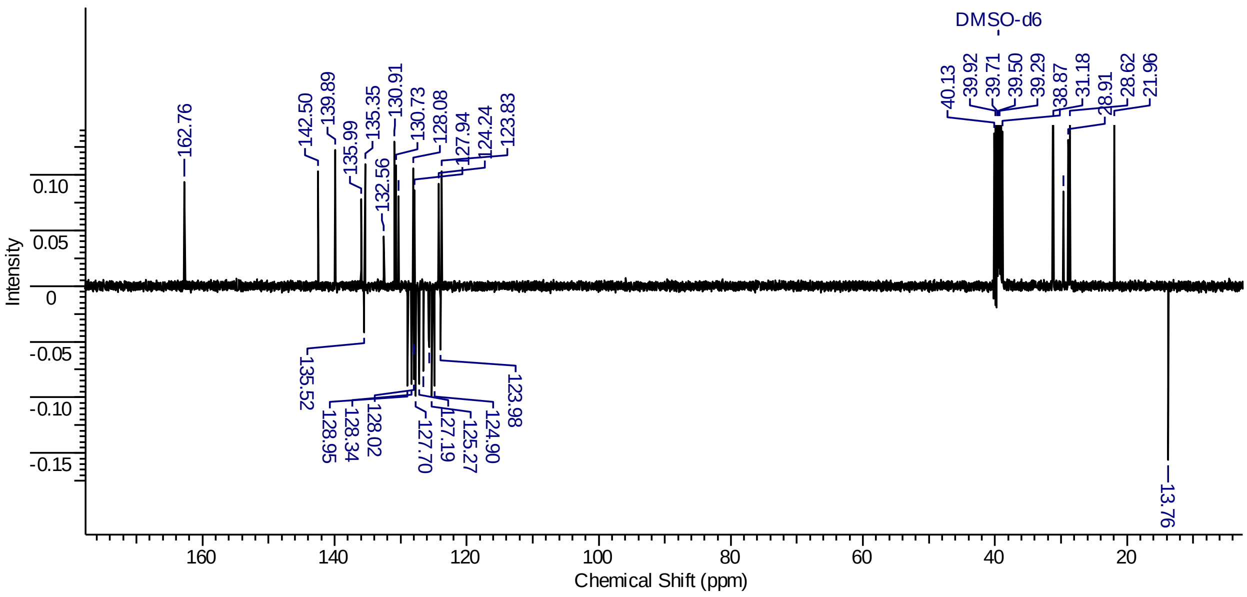

3-Decyl-2,2':5',2''-terthiophene-5-carbohydrazide (13c)

^{1}H NMR (400 MHz, $CDCl_3$)

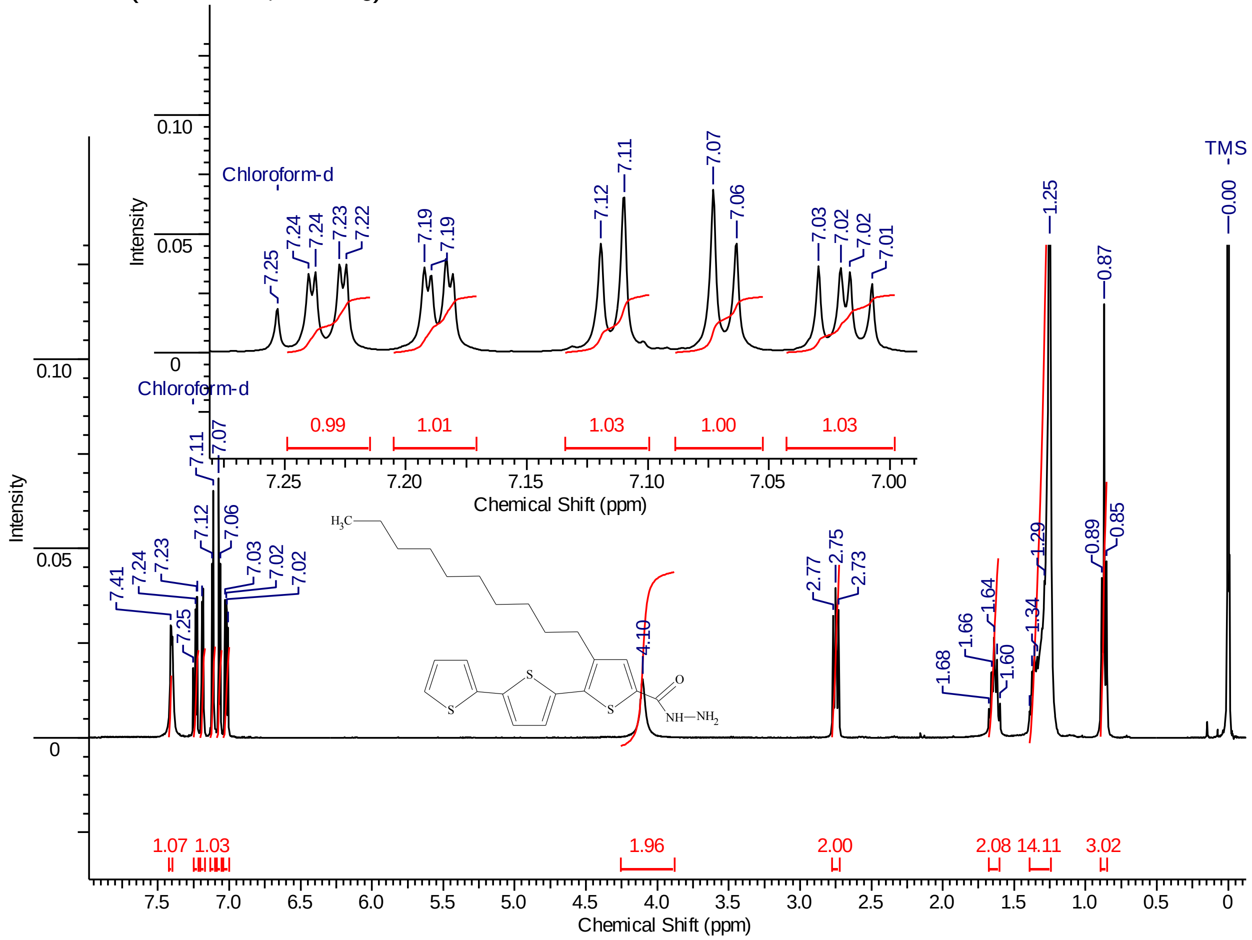

^{13}C NMR (100 MHz, δ, ppm) **(13c)**

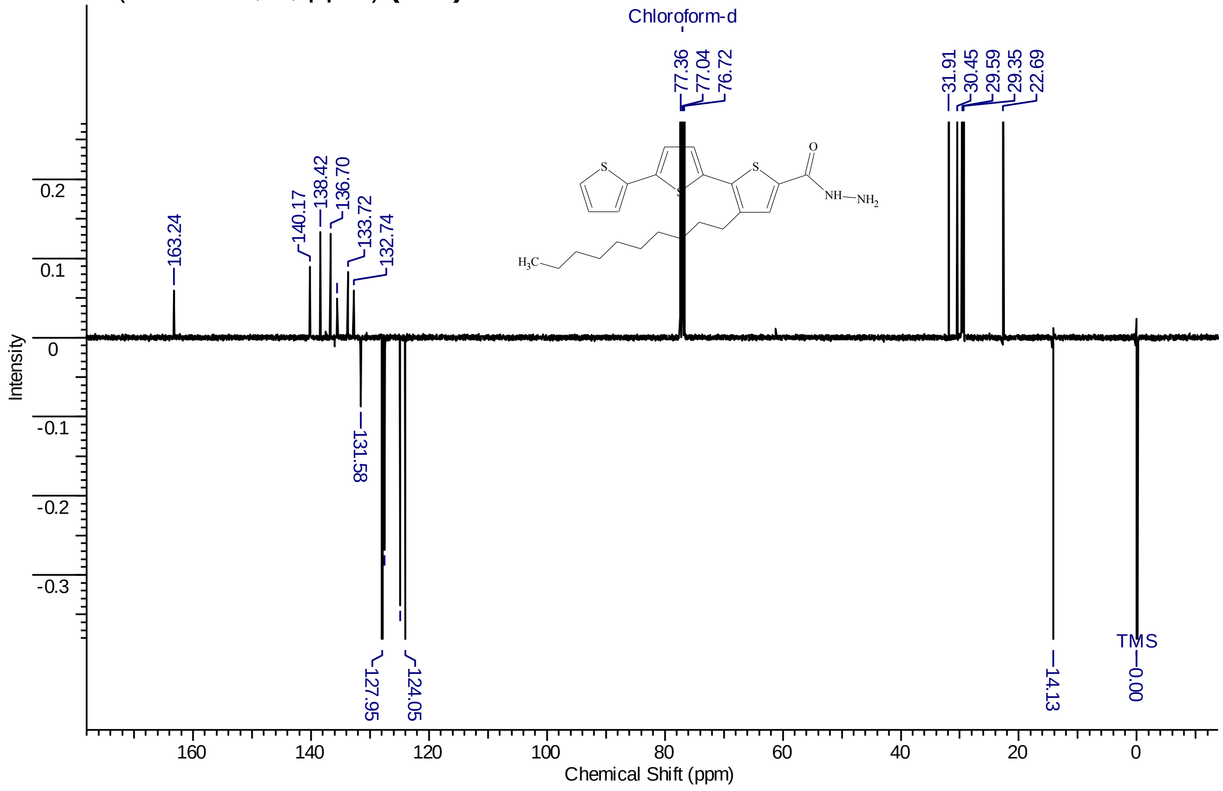

N,*N'*-Bis(5'-phenyl)-[3-decyl-2,2'-bithiophen-5-carbonyl]hydrazine (14d)

^{1}H NMR (400 MHz, $CDCl_3$)

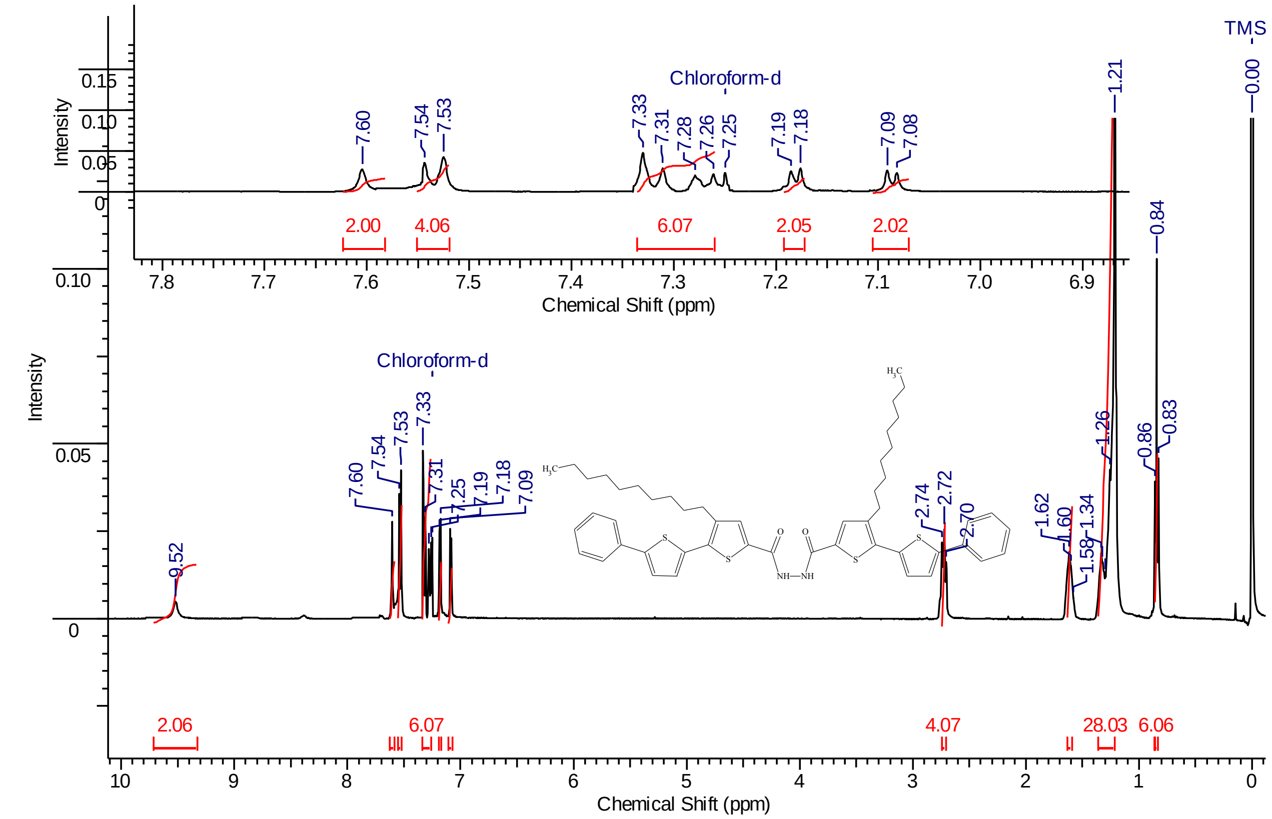

^{13}C NMR (100 MHz) **(14d)**

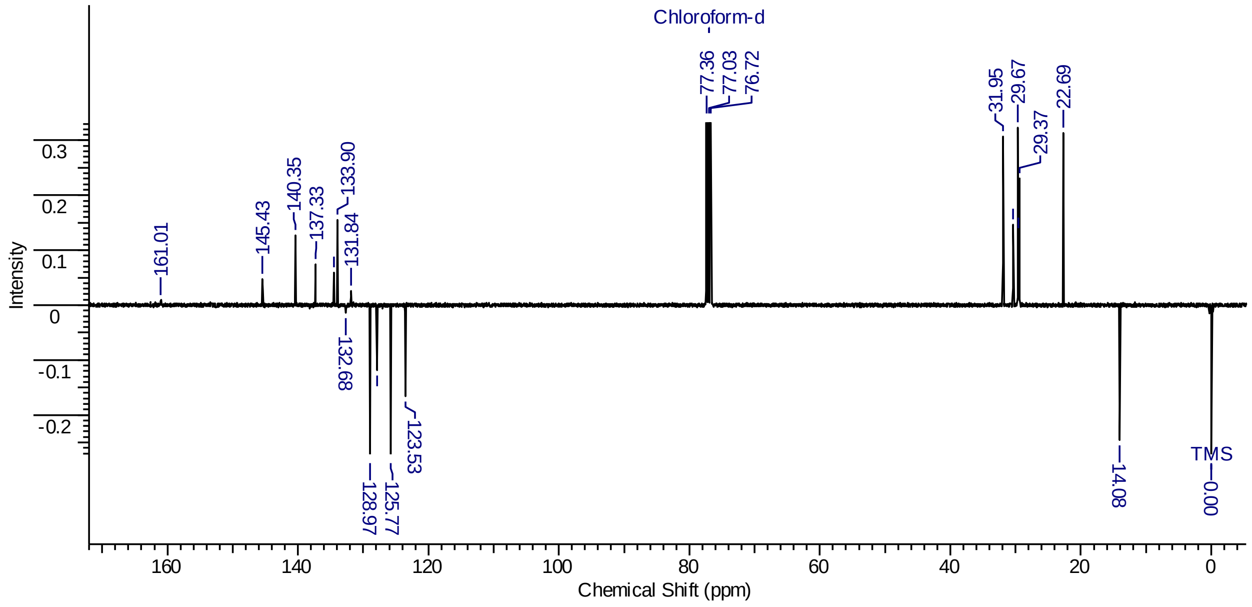

N,N'-Bis(5'-(naphthalen-1-yl)-[3-decyl-2,2'-bithiophen-5-carbonyl]hydrazine (14e)

^{1}H NMR (400 MHz, $CDCl_3$)

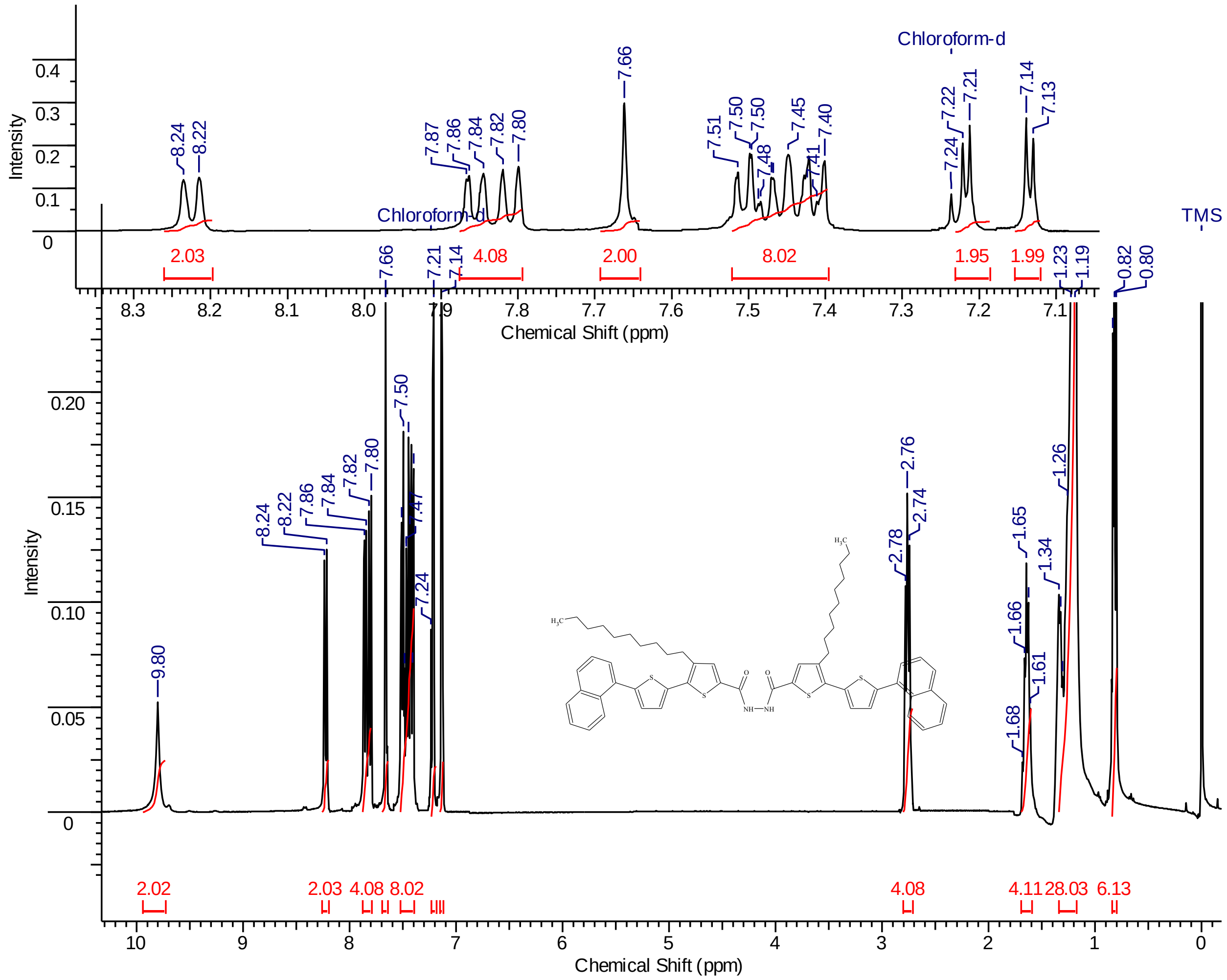

^{13}C NMR (100MHz) **(14e)**

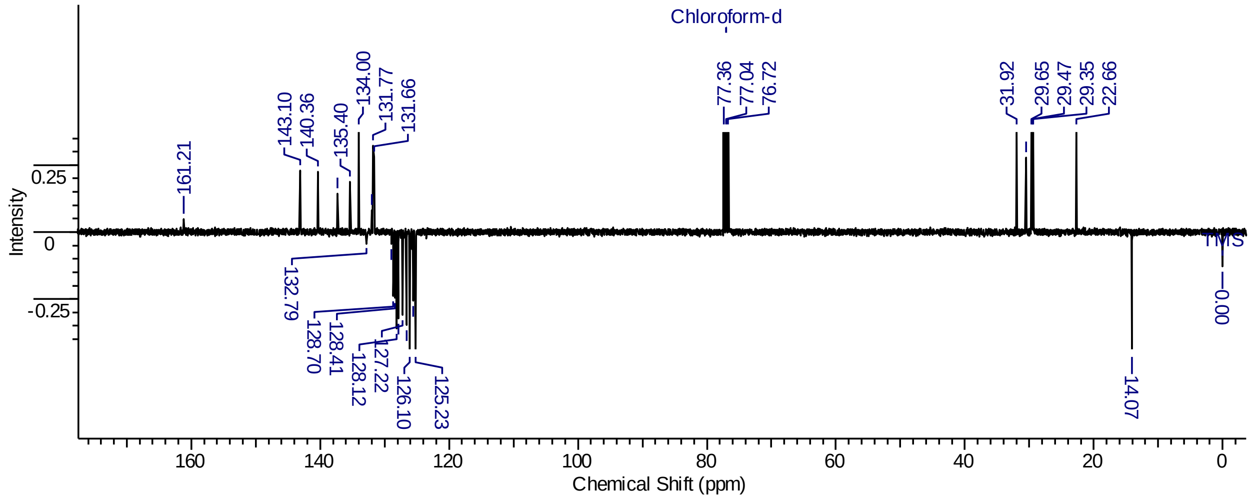

***N*,*N'*-Bis(5'-(naphthalen-1-yl)-[3-decyl-2,2'-bithiophen-5-carbonyl]hydrazine (14f)**

^{1}H NMR (400 MHz, DMSO-d_6, 70 °C)

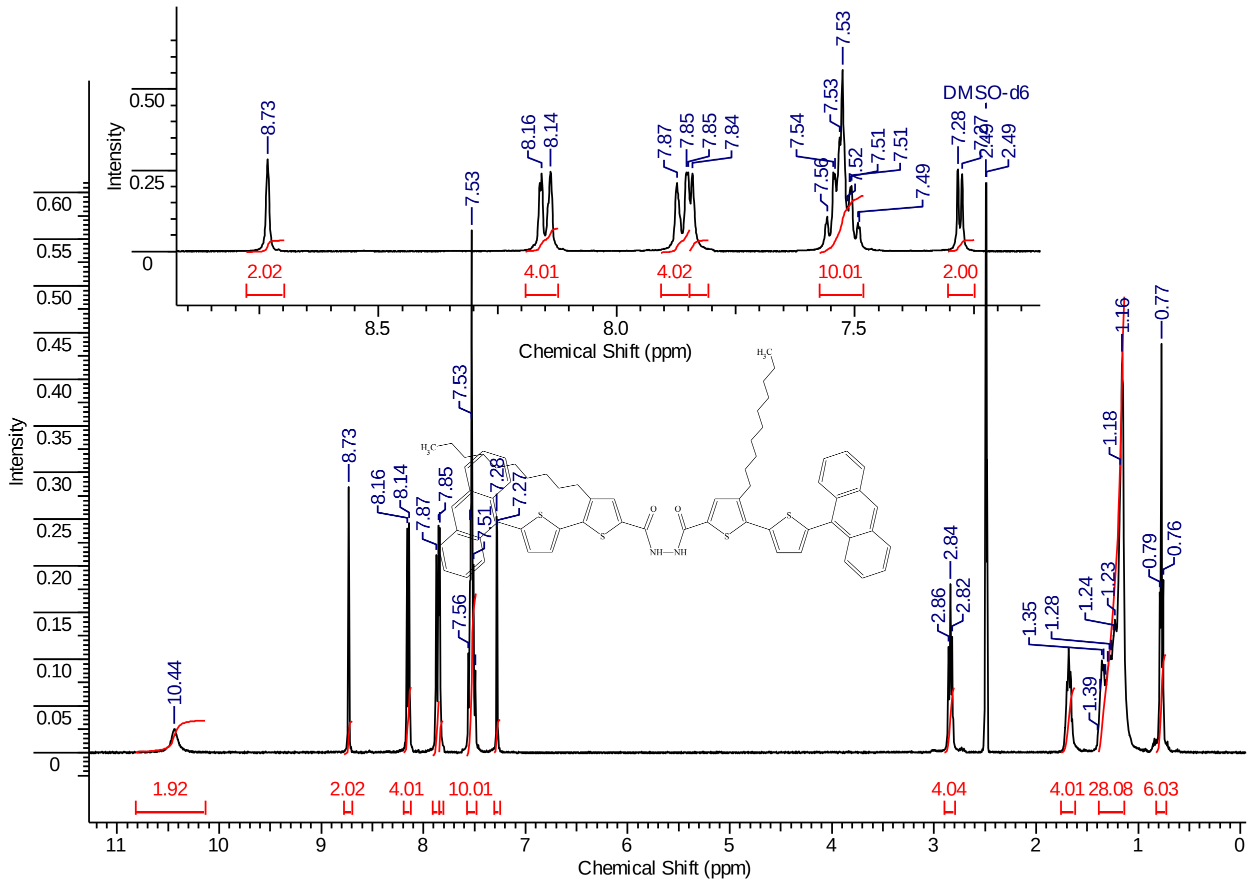

^{13}C NMR (100MHz) **(14f)**

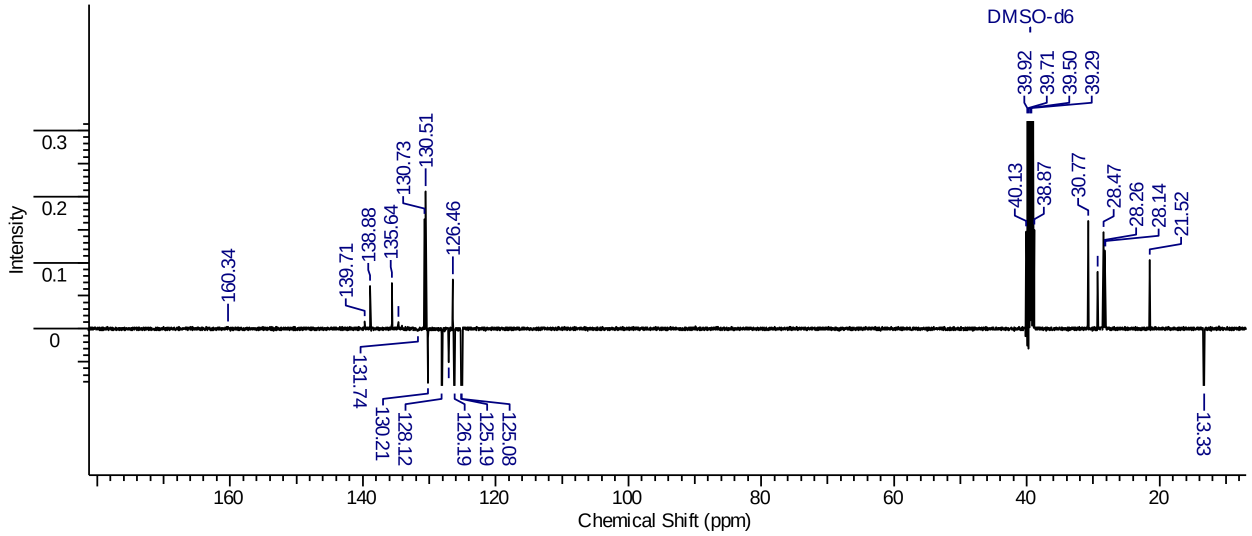

N,*N'*-Bis(5'-(pyren-1-yl)-[3-decyl-2,2'-bithiophen-5-carbonyl]hydrazine (14g)

^{1}H NMR (400 MHz, DMSO-d_6, 70 °C)

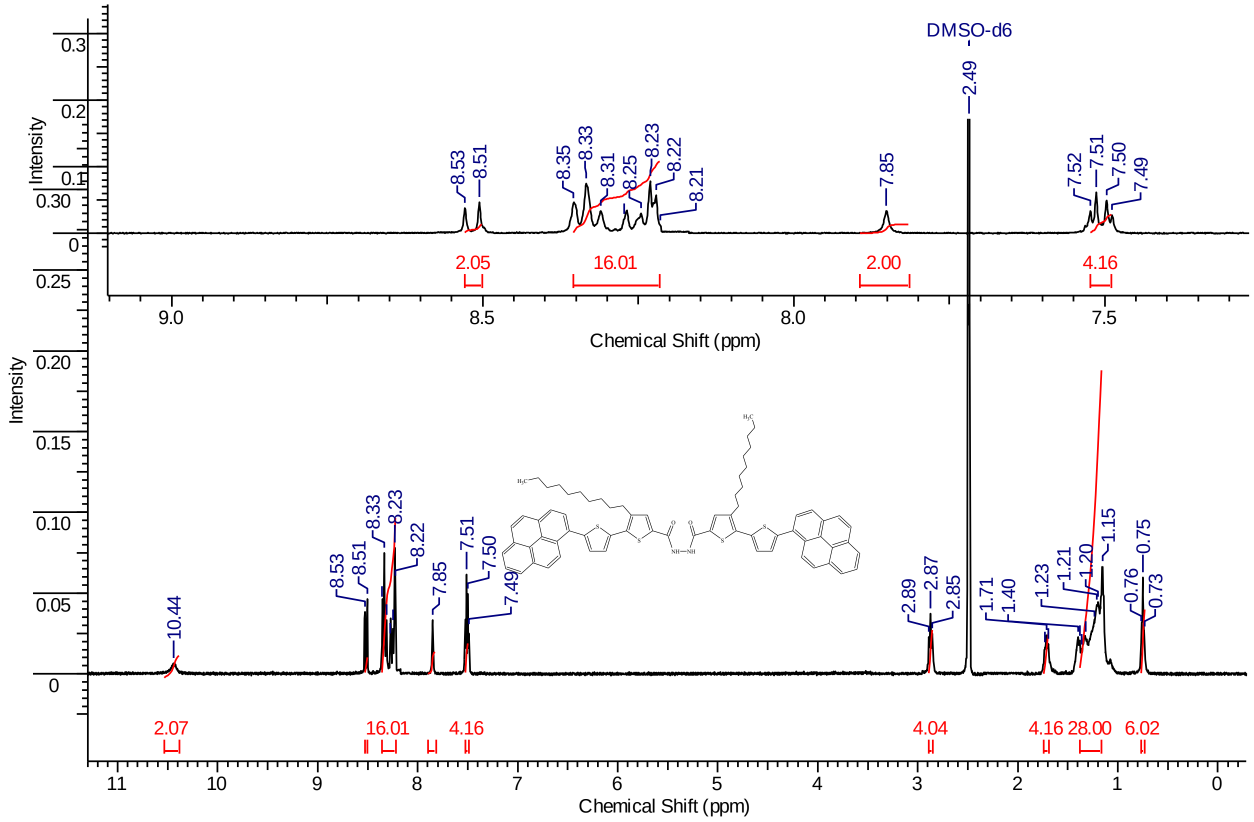

^{13}C NMR (100 MHz) **(14g)**

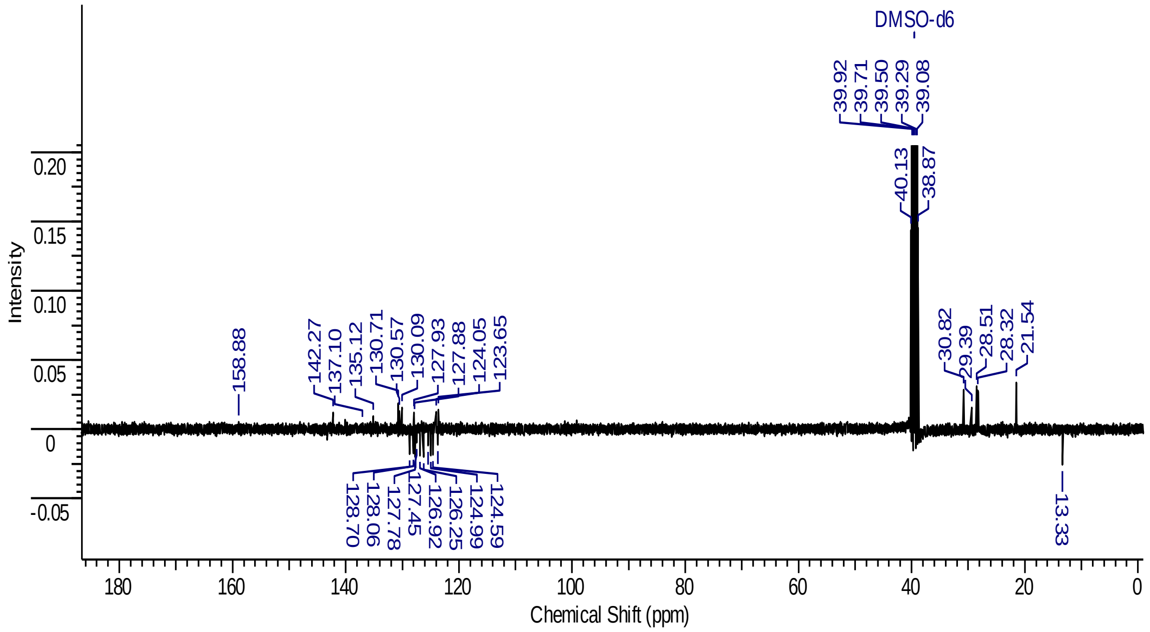

N,*N*'-Bis(3-decyl-2,2':5',2''-terthiophen-5-carbonyl)hydrazine (14c)

^{1}H NMR (400 MHz, DMSO-d_6)

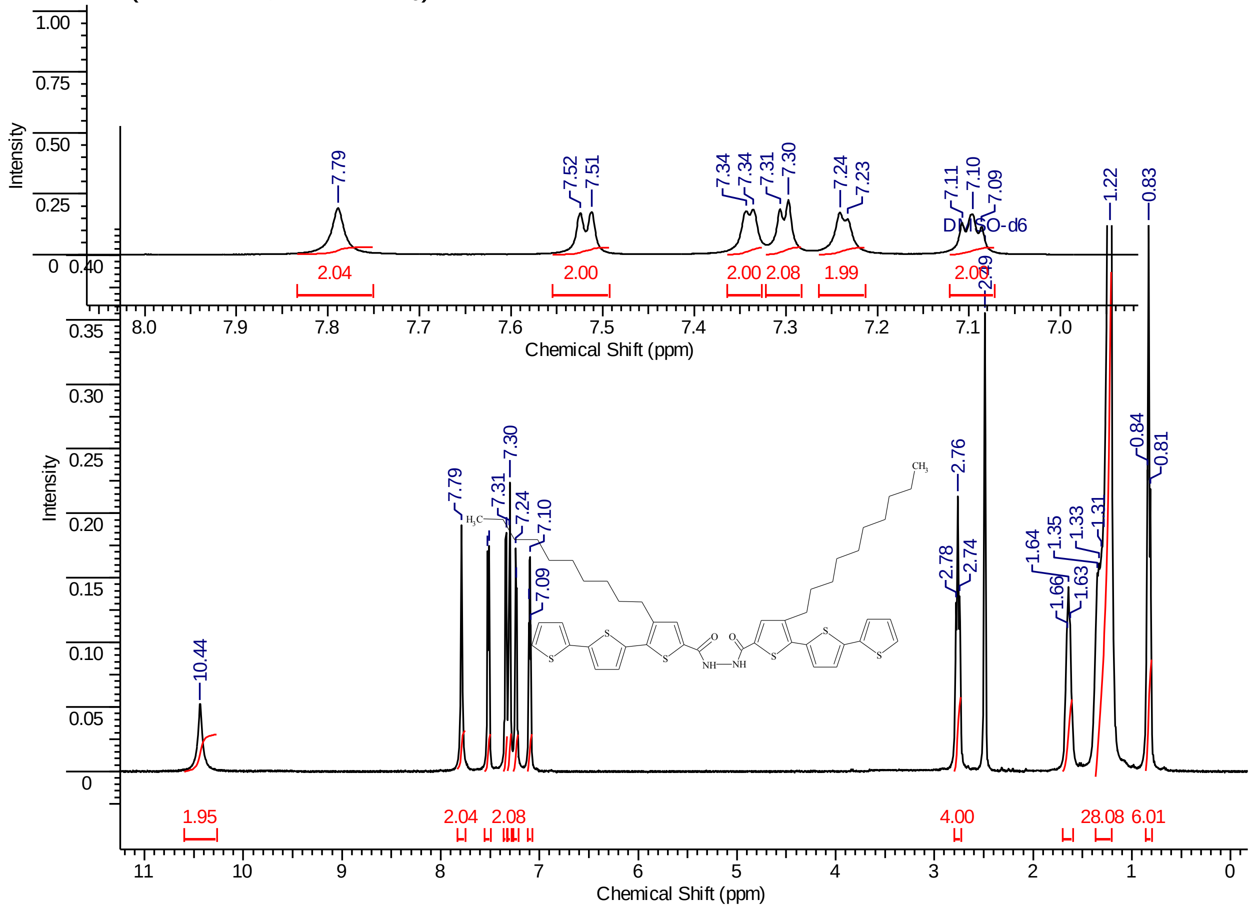

^{13}C NMR (100 MHz, δ, ppm) **(14c)**

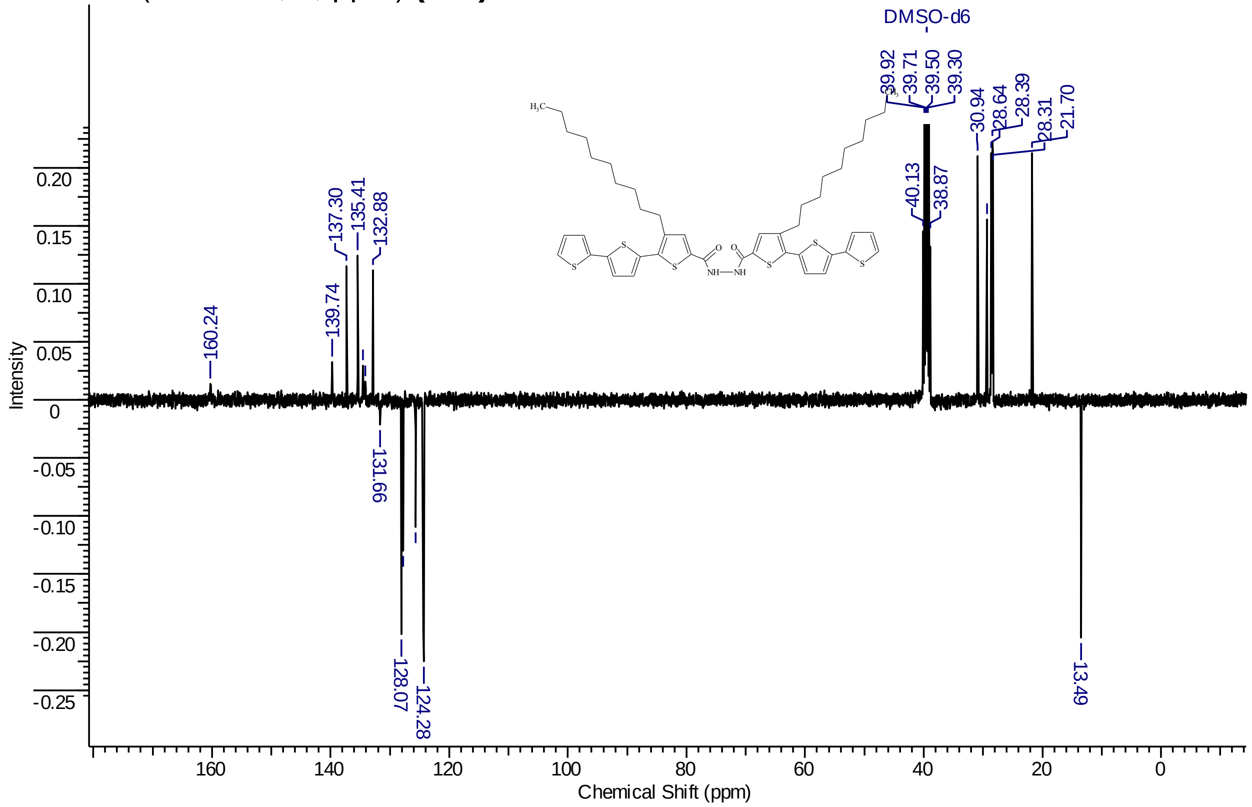

2,5-Bis(3-decyl-5'-phenyl-2,2'-bithiophen-5-yl)-1,3,4-oxadiazole (15d)

^{1}H NMR (400 MHz, $CDCl_3$)

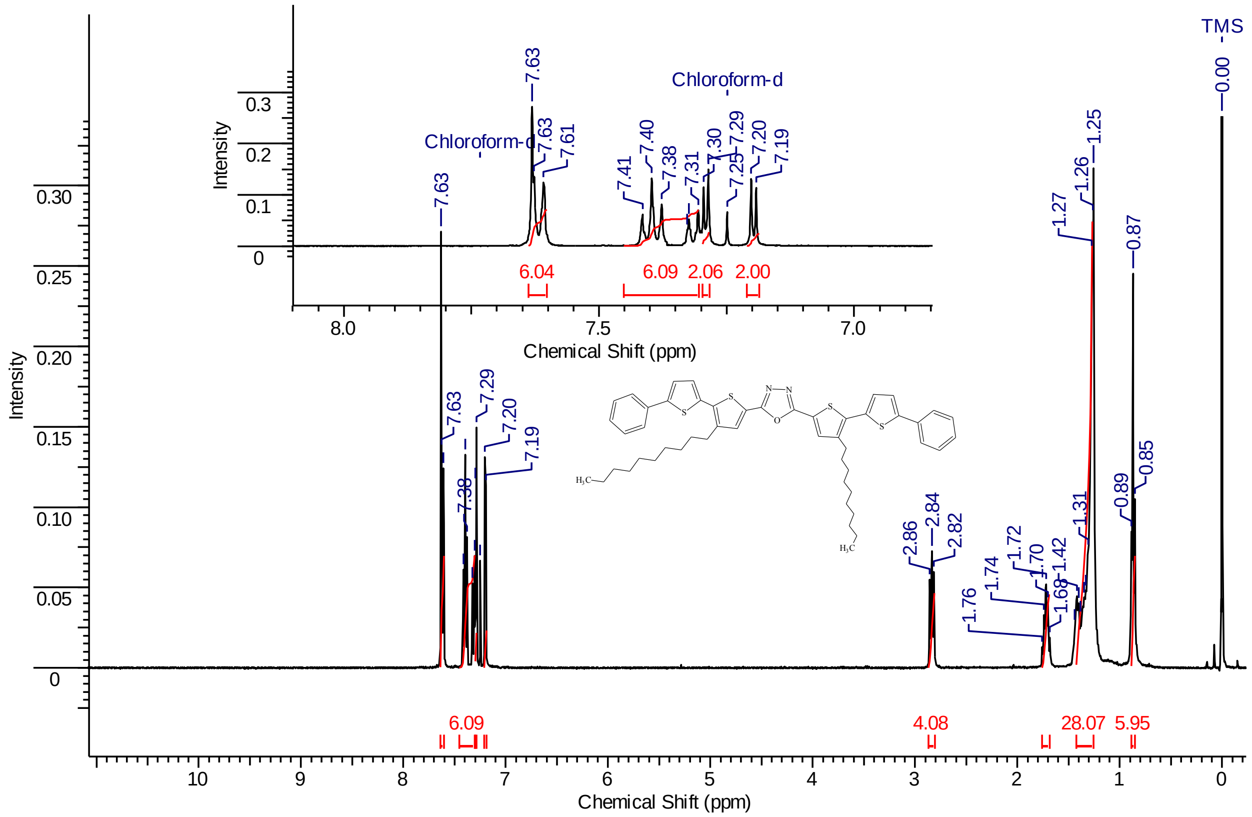

^{13}C NMR (100 MHz) **(15d)**

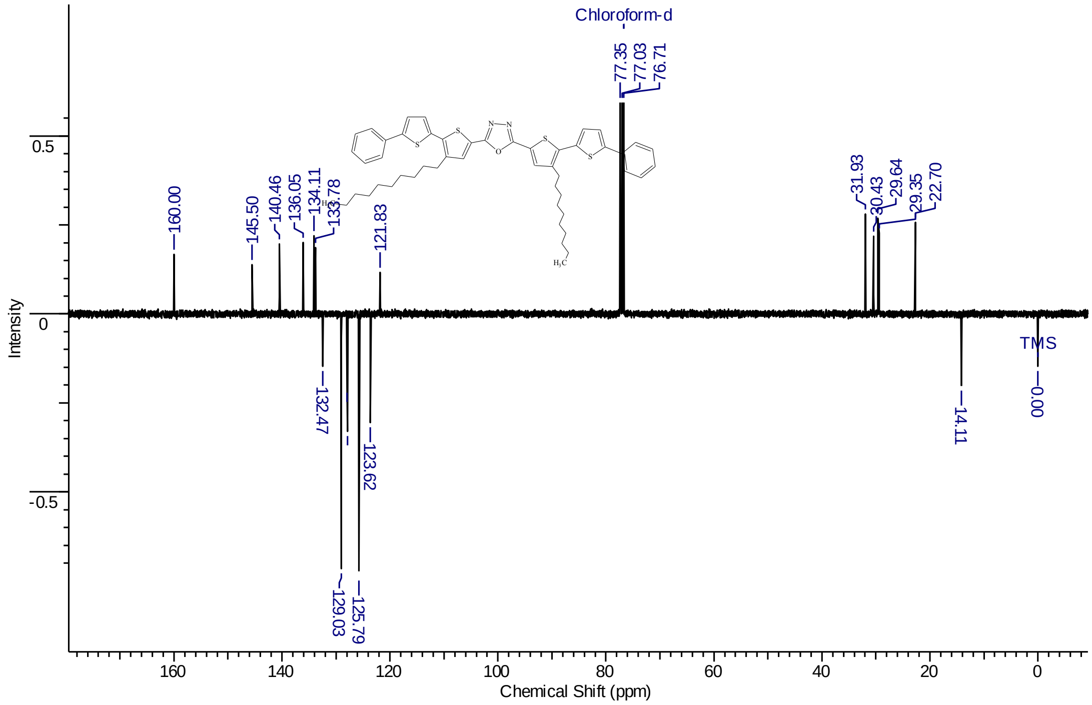

2,5-Bis(3-decyl-5'-(naphthalen-1-yl)-2,2'-bithiophen-5-yl)-1,3,4-oxadiazole (15e)

^{1}H NMR (400 MHz, $CDCl_3$)

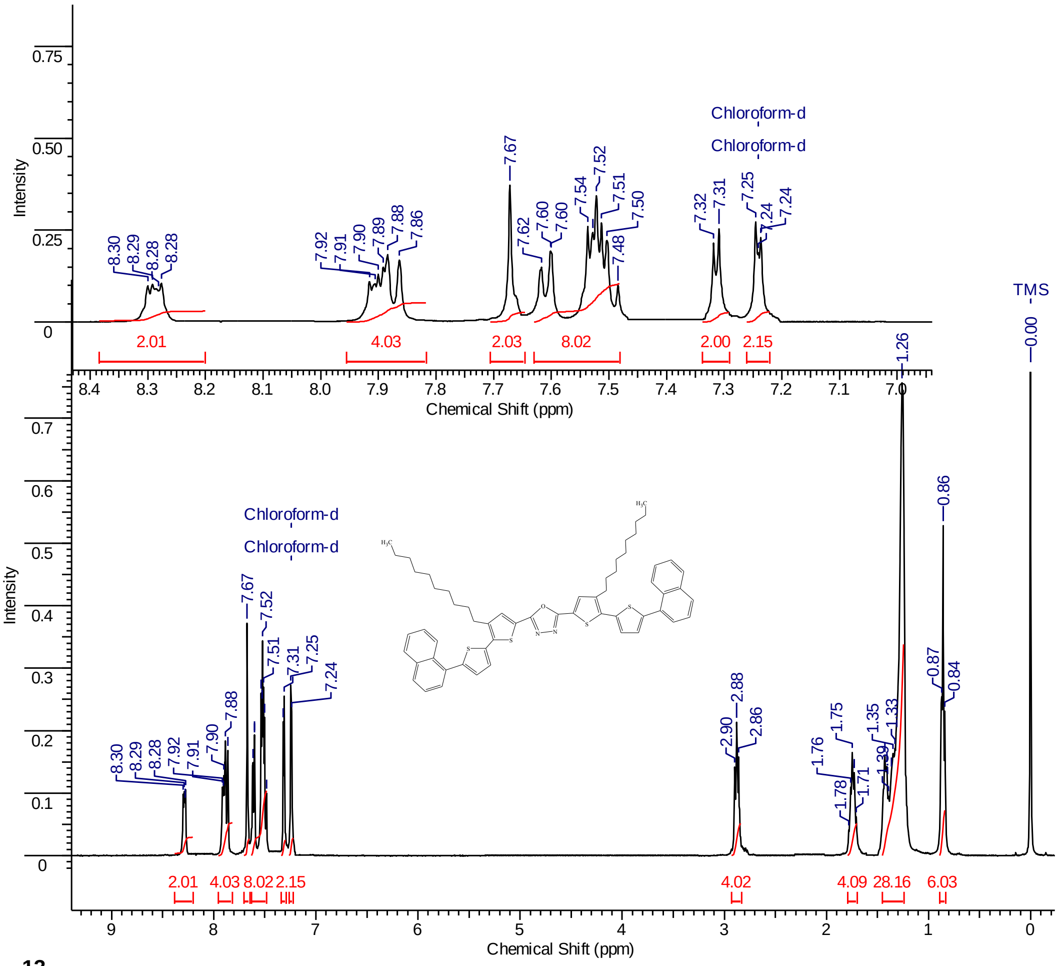

^{13}C NMR (100 MHz) (15e)

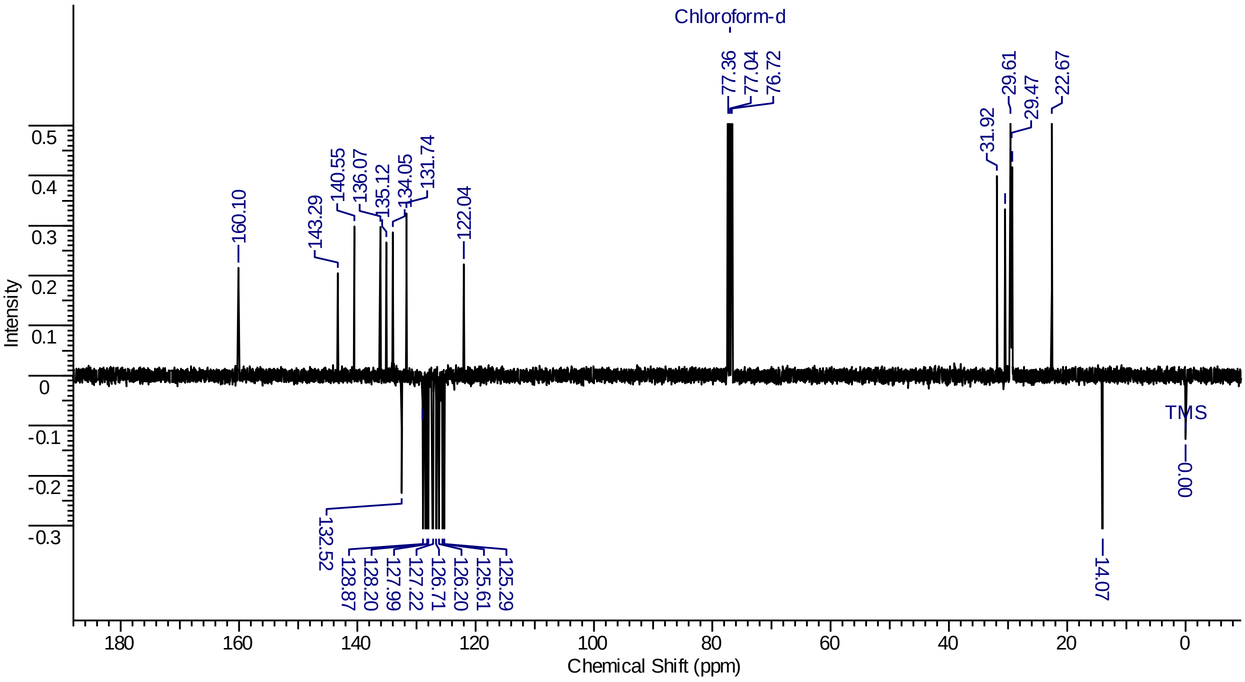

2,5-Bis(5'-(anthracen-9-yl)-3-decyl-2,2'-bithiophen-5-yl)-1,3,4-oxadiazole (15f)

^{1}H NMR (400 MHz, $CDCl_3$) **(15f)**

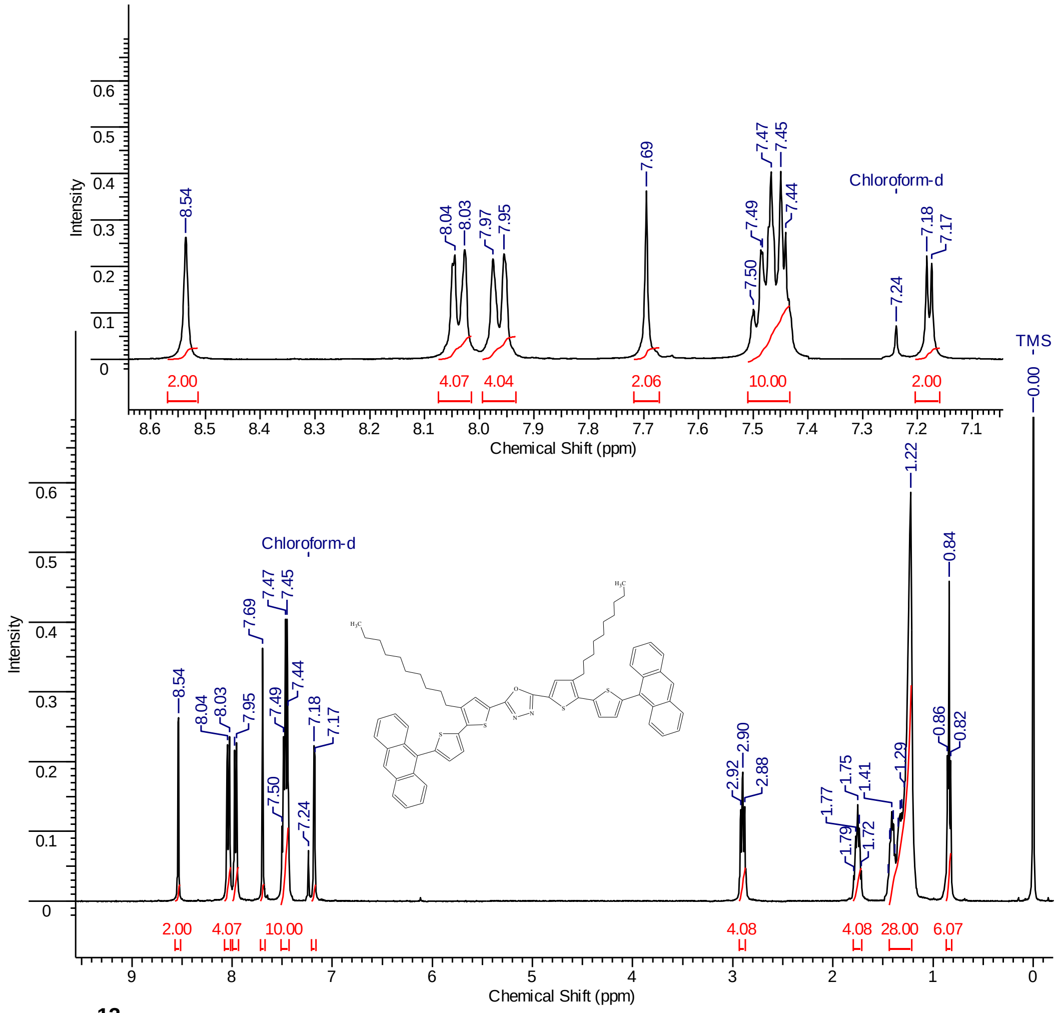

^{13}C NMR (100 MHz) **(15f)**

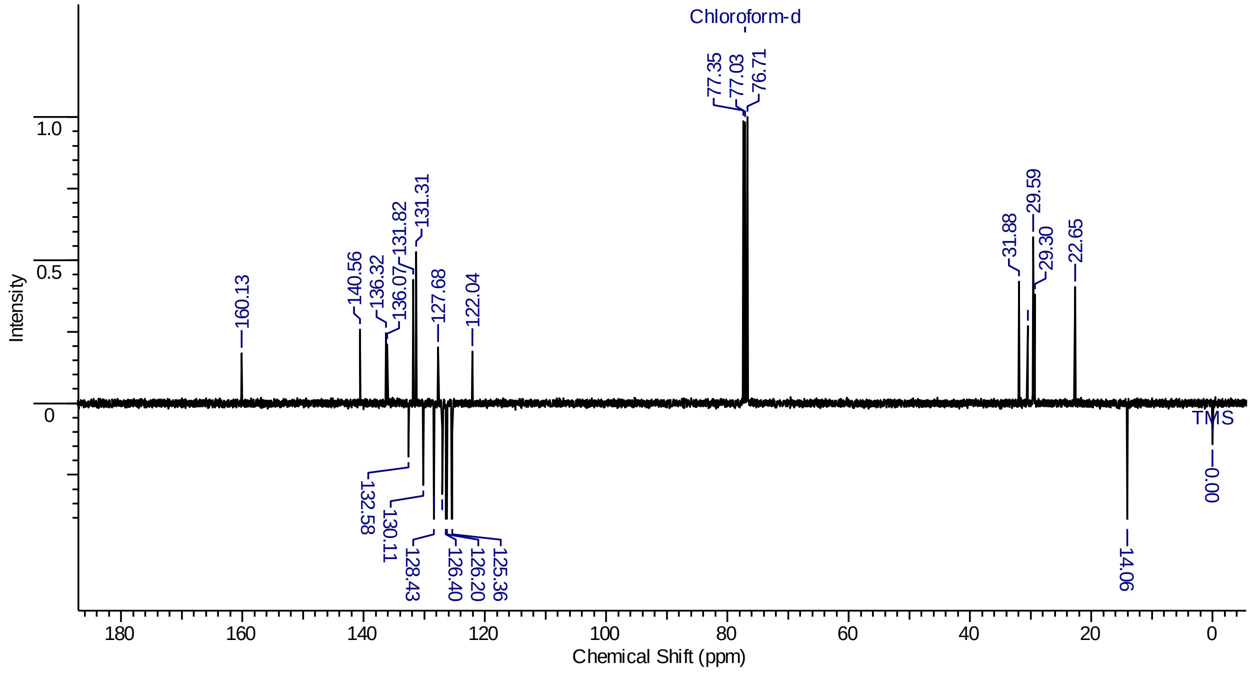

2,5-Bis(3-decyl-5'-(pyren-1-yl)-2,2'-bithiophen-5-yl)-1,3,4-oxadiazole (15g)

^{1}H NMR (400 MHz, $CDCl_3$)

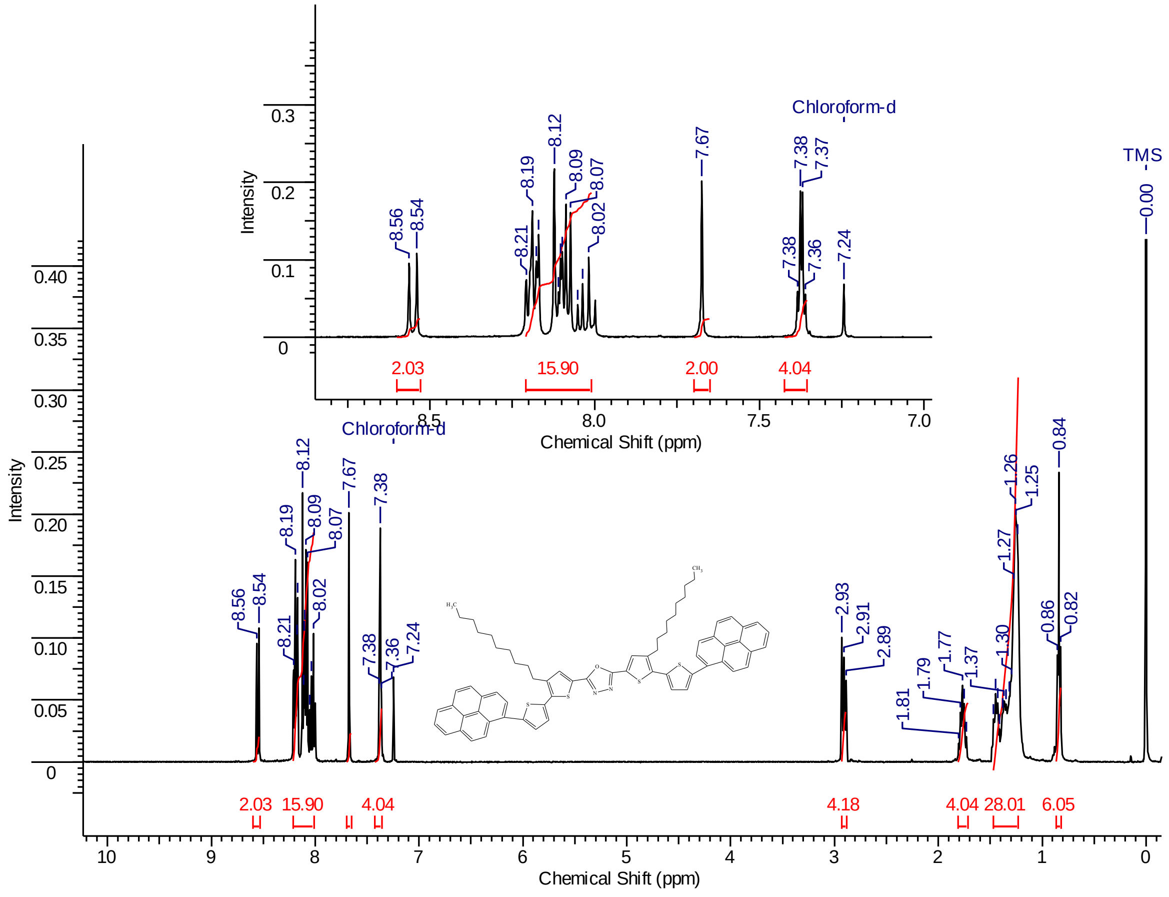

^{13}C NMR (100 MHz) **(15g)**

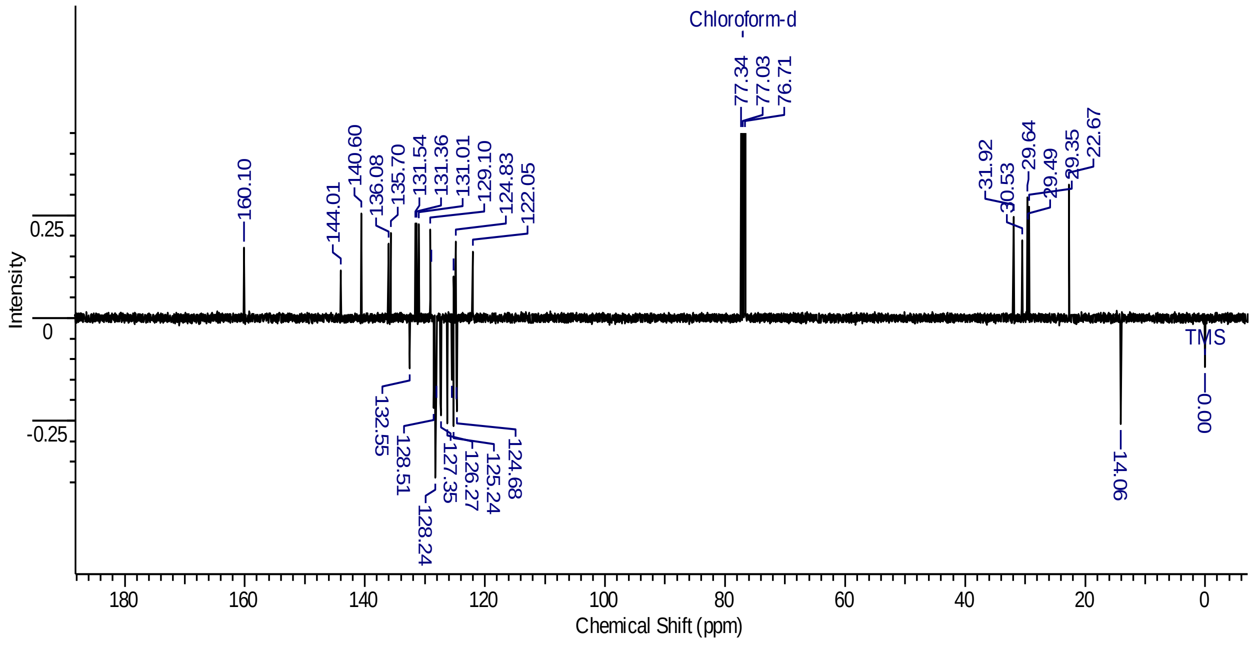

2,5-Bis(3-decyl-2,2':5',2''-terthiophen-5-yl)-1,3,4-oxadiazole (15c)

^{1}H NMR (400 MHz, $CDCl_3$)

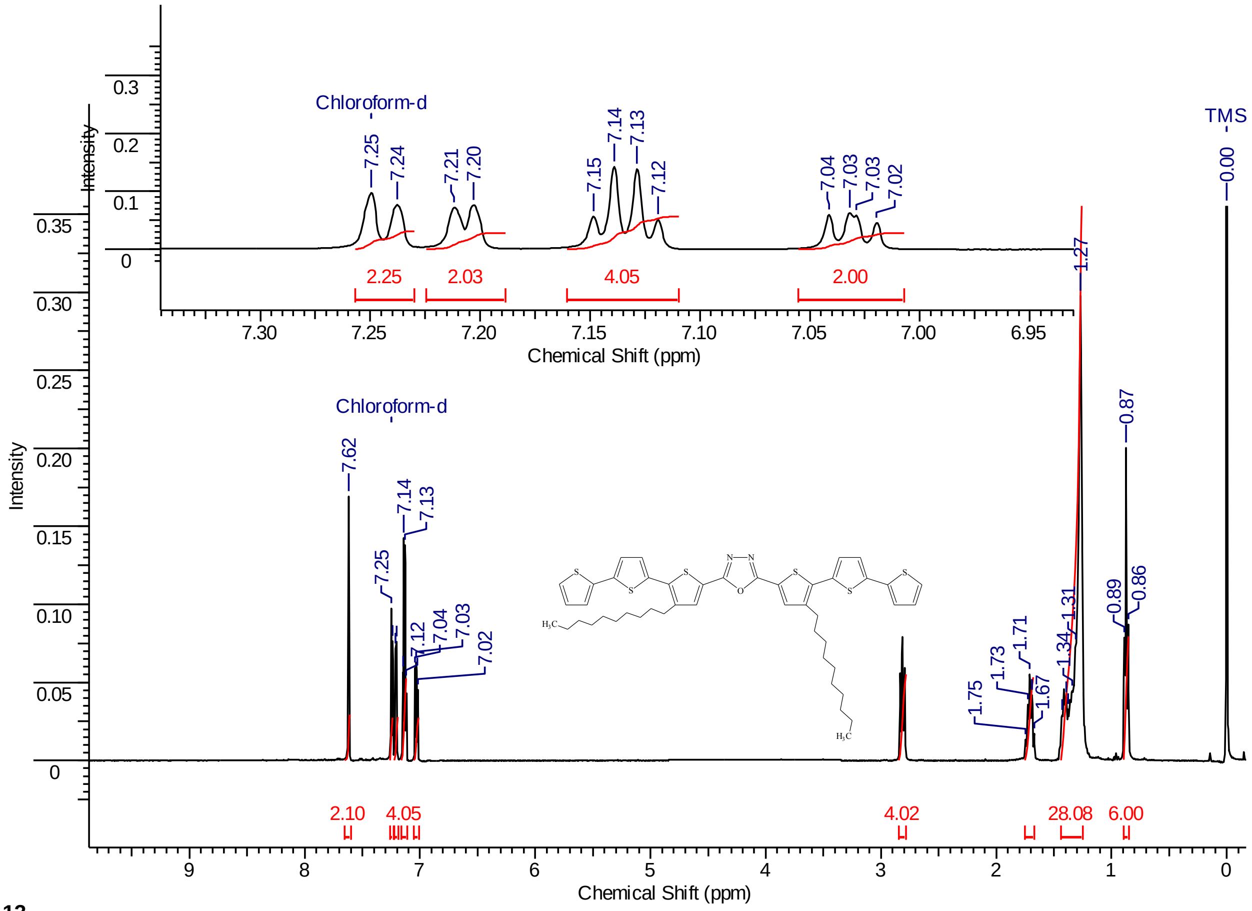

^{13}C NMR (100 MHz, δ, ppm) **(15c)**

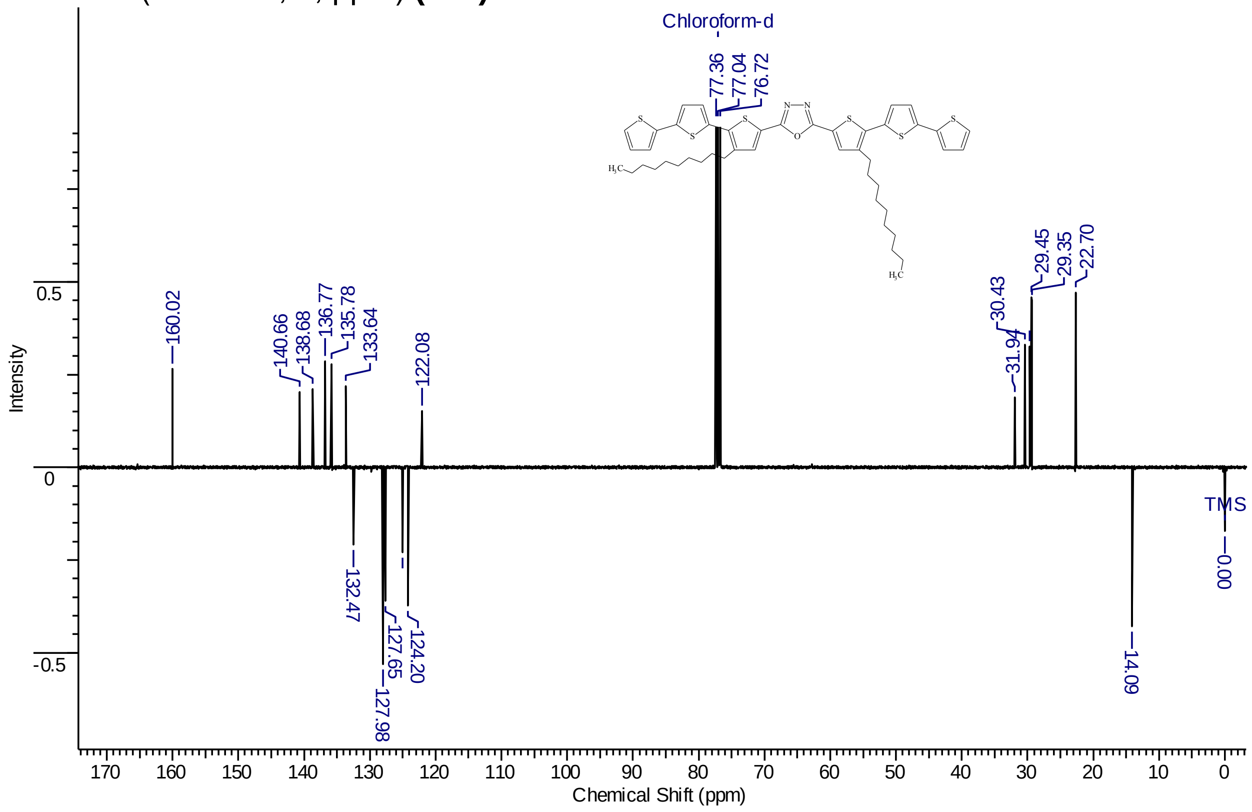

Supplement: File 1 — Experimental, computational and analytical data [file Beilstein_J_Org_Chem-13-313-s001.pdf]
